# Supplementary material for: Evaluation of routinely collected records for dementia outcomes in UK: a prospective cohort study
Source: BMJ Open. 2022 Jun 15;12(6):e060931. doi: 10.1136/bmjopen-2022-060931 (PMC9204445; doi:10.1136/bmjopen-2022-060931)
Supplement: Supplementary data [file bmjopen-2022-060931supp001.pdf]

Supplementary Information

Title: Evaluation of routinely collected records for dementia outcomes in United Kingdom (UK): a longitudinal study.

Authors: Shabina A Hayat, Robert Luben, Kay-Tee Khaw, Nick Wareham, Carol Brayne.

Methods

Dementia ascertainment and diagnostic codes in hospital, death records and mental healthcare data

Individuals were followed from the date of consent, until the earliest date of a dementia diagnosis or date of death in or after 1996 when formal follow up by health records began in EPIC-Norfolk. To maximise the outcome data, we included any-cause dementia, the sub types of dementia were not analysed separately. Participants with no reports of death or dementia were censored on 31st December 2018. Dementia from HES records, death certificate or the MHDS was defined as any of the ICD-10 codes as listed in Supplementary Table S1.

Dementia case ascertainment through GP records

The protocol was based on a 'clean up' exercise [1] recommended by clinical commissioning groups (NHS bodies that commission services for defined populations) to improve dementia diagnosis in general practices. [2] We employed Read codes (Supplementary Table S1) identified as most commonly used for a dementia diagnoses. [1] Twenty-six collaborating GP practices were invited to participate. Those practices that agreed to take part in this exercise were provided with a detailed protocol and a list of patients on their register who were also EPIC-Norfolk participants. Each practice ran searches to generate lists of these respondents to establish which had a dementia diagnosis code, or were prescribed dementia defining medication, Donepezil, Galantamine, Rivastigmine or Memantine as identified by the relevant Read code (Supplementary Table S1).

Data were returned via a secure transfer system. Practices were also asked to complete a simple questionnaire. The full protocol and sample questionnaire are available on request. Practices were advised to take advantage of this exercise by comparing results of the search against their Quality and Outcomes Framework (QOF) [3] dementia register to check for discrepancies to improve their dementia diagnosis rates. [1] QOF for dementia coding in primary care financially incentivises each recorded dementia diagnosis.

Hayat S, *et al. BMJ Open* 2022; 12:e060931. doi: 10.1136/bmjopen-2022-060931

In the second phase, cases identified from GP records were cross-checked with the NHS Digital data, and the level of agreement between the data sources was examined. GP records of individuals with a dementia diagnosis in secondary care but missing from the initial GP data extract were further reviewed by researchers in the third and final confirmation phase of this study. Individual records were reviewed by the researchers for any dementia read or ICD code or mention of dementia as free text. Initial case ascertainment from GPs was conducted between 2016-2017 and NHS Digital data were up to 31st March 2018, with case confirmation in GP records up to 20th December 2018, allowing few months after the follow-up period for the NHS Digital (secondary care) data.

Co-variates included in analyses

Education (the highest level attained) and social class were obtained from the baseline questionnaire. Education was categorised into three groups (i) No qualification (not completing school up to the age of 16), (ii) Completion of school up to the age of 16 or up to the age of 18 and finally (iii) those obtaining an education to graduate level (those who obtained a degree or equivalent) or above.

Social class was classified according to the Registrar General's occupation-based classification scheme into five main categories. [4] Social class I consists of professionals, class II includes managerial and technical occupations, class III is subdivided into non-manual and manual skilled workers (III non-manual and III manual), class IV consists of partly skilled workers, and class V comprises unskilled manual workers.

Hayat S, *et al. BMJ Open* 2022; 12:e060931. doi: 10.1136/bmjopen-2022-060931

Table S1: Read and ICD 10 codes used in EPIC-Norfolk to search for 'Definite' dementia cases

Read codes	ctv3_codes	Read Code Description	ICD Code	ICD Description
**Eu00**	**Eu00**	Dementia in Alzheimer's disease	**F00**	Dementia in Alzheimer's disease
			**G30**	Alzheimer's disease
**Eu000**		Dementia in Alzheimer's disease with early onset	**F00.0**	Dementia in Alzheimer's disease with early onset
			**G30.0**	
**Eu001**	**X0030**	Dementia in Alzheimer's disease with late onset	**F00.1**	Dementia in Alzheimer disease with late onset
			**G30.1**	
	**XaIKC**	Alzheimer's disease with late onset		
**Eu002**	**Eu002**	Dementia in Alzheimer's disease, atypical or mixed type	**F00.2**	Dementia in Alzheimer disease, atypical or mixed type
			**G30.8**	Other Alzheimer's disease
**F110**		Dementia in Alzheimer's disease, unspecified	**F00.9**	Dementia in Alzheimer's disease, unspecified
			**G30.9**	
**Eu01**	**XE1Xs**	Vascular dementia	**F01**	Vascular dementia
			**F01.0**	Vascular dementia of acute onset
**Eu011**	**Xa0IH**	Multi-infarct dementia	**F01.1**	Multi-infarct dementia
**Eu012**	**X003T**	Subcortical vascular dementia	**F01.2**	Subcortical vascular dementia
**Eu01y**	**Eu01y**	Other vascular dementia	**F01.8**	Other vascular dementia
**Eu01z**	**Eu01z**	Vascular dementia, unspecified	**F01.9**	Vascular dementia, unspecified
			**F02**	Dementia in other diseases classified elsewhere
**Eu020/F111**	**X0034/F111**	Picks Disease (Read)/Frontotemporal dementia includes Picks Disease and progressive isolated aphasia(CTv3)	**F02.0**	Dementia in Pick's disease
			**F02.1**	Dementia in Creutzfeldt-Jakob disease
			**F02.2**	Dementia in Huntington's disease
**Eu023**	**Eu023**	Dementia in Parkinson's disease	**F02.3**	Dementia in Parkinson's disease
			**F02.8**	Dementia in other specified diseases classified elsewhere

Table S1: Continued

**Eu02y**	**X0034**	Dementia in other diseases specified elsewhere (Read)/Frontotemporal dementia includes Pick's Disease and progressive isolated aphasia	**G31**	Other degenerative diseases of nervous system, not elsewhere classified
			**G31.0**	Frontotemporal dementia
**Eu025 /F116**	**X003A**<br>**XaKyY**	Lewy body dementia<br>Lewy body dementia	**G31.8**	Other specified degenerative diseases of nervous system<br>Grey-matter degeneration [Alpers]<br>Lewy body(ies)(dementia)(disease)<br>Subacute necrotizing encephalopathy [Leigh]
**Eu02z**	**XE1Z6**	Unspecified dementia	**F03**	Unspecified dementia
			**F05.1**	Delirium superimposed on dementia
	**X002w**	Dementia		
	**X00R2**	Senile dementia		
**E00..**	**E00..**	Senile/presenile dementia		
**1461**	**1461**	History of dementia		
**Eu107**<br>**Eu10711**<br>**E012**<br>**E0120**	**Xa25J**	Alcoholic dementia	**F10.7**	Residual and late-onset psychotic disorder: Includes Alcoholic dementia NOS<br>Chronic alcoholic brain syndrome<br>Dementia and other milder forms of persisting impairment of cognitive functions

Anti-Dementia Medications

- Donezepil (Aricept®Aricept Evess®) Read Code: dy1
- Galantamine (Reminyl®, Reminyl® XL) Read Code: dy3…
- Rivastigimine (Exelon) Read Code: dy2…
- Memantine hydrochloride Read Code: dB1..

Hayat S, *et al. BMJ Open* 2022; 12:e060931. doi: 10.1136/bmjopen-2022-060931

Table S2: Comparison of socio-demographic characteristics between sub-population (N=4668) and rest of EPIC-Norfolk cohort.

	**Cohort**		**GP Sub-cohort**		**P-Value**
	**N=25 777***		**N=4668**		
Socio-demographic					
**Mean (SD)**					
**Age at Baseline**	59.9	(9.6)	56.2	(8.0)	<0.001
**Age at dementia diagnosis**	83.7	(6.6)	84.4	(6.6)	0.7
**Sex, % women (n)**	54.6	(14 063)	57.4	(2681)	<0.001
**Education, % (n)**					
No qualifications	39.8	(10 240)	33.1	(1546)	
O/ A level Standard	48.6	(12 510)	51.6	(2407)	<0.001
Graduate Level	11.6	(2992)	15.2	(711)	
**Social Class, % (n)**					
Professional	6.4	(1616)	8.5	(388)	
Managerial	35.2	(8833)	35.5	(1627)	
Skilled Non-Manual	16.7	(4199)	15.2	(697)	<0.001
Skilled Manual	23.7	(5948)	23.2	(1062)	
Semi-Skilled	14.1	(3545)	13.0	(597)	
Non-Skilled	3.8	(945)	4.6	(211)	
Dementia, % (N)	10.1	(2609)	4.4	(209)	<0.001

* Using full cohort as the sub population was based on this denominator and not 25,639 from baseline

Hayat S, *et al. BMJ Open* 2022; 12:e060931. doi: 10.1136/bmjopen-2022-060931

Table S3: Summary of responses from 8 of the collaborating Practices providing GP data.

Response from Questionnaire sent to GP Practice	Number
Used both Read Code and Medication names in searches	7
Included additional dementia codes not listed in the 'Clean up exercise' protocol	4
Information available in records as coded and free text data (free text not provided for this study)	6 (0 Practices used free text alone)
Practices experiencing difficulty with using dementia Read Codes	6=No 1=Yes<br>1-No response
Does the Practice carry out In-house cognitive testing (Yes)	7
Reason for patient cognitive assessment/referral to memory clinic	7=Patient concern<br>6= GP Judgement<br>6= Opportunistic testing
How is a confirmed dementia diagnosis made	8= Following secondary referral<br>4= including GP judgement
How is information of a dementia diagnosis received by the Practice	7= Free text (As a letter)<br>1= Free text plus ICD code
How is information of a dementia diagnosis entered onto the local GP database	7= Read Code<br>1= (Free text, ICD and Read code)
Were all cases identified from searches already included on QOF* register	3=Yes<br>5=No
Was there a discrepancy between QOF* register and Read Code search	5=Yes<br>3=No

* Quality and Outcomes Framework

Dementia from HES records, death certificate or the MHDS was defined as any of the ICD-10 codes as listed in Supplementary Table S1.

References

1 Russell P, Banerjee S, Watt J, *et al.* Improving the identification of people with dementia in primary care: evaluation of the impact of primary care dementia coding guidance on identified prevalence. *BMJ Open* 2013;**3**:e004023. doi:10.1136/bmjopen-2013-004023

2 London Clinical Networks. "Coding clean-up ” Exercise Guidance to GPs to improve dementia coding , and raise diagnosis rates: A step by step approach. London, UK:

3 Mukadam N, Livingston G, Rantell K, *et al.* Diagnostic rates and treatment of dementia before and after launch of a national dementia policy: An observational study using English national databases. *BMJ Open* 2014;**4**:1–8. doi:10.1136/bmjopen-2013-004119

4 Shohaimi S, Welch A, Bingham S, *et al.* Residential area deprivation predicts fruit and vegetable consumption independently of individual educational level and occupational social class: a cross sectional population study in the Norfolk cohort of the European Prospective Investigation into Canc. *J Epidemiol Community Health* 2004;**58**:686–91. doi:10.1136/jech.2003.008490
