# Supplementary material for: Evaluation of routinely collected records for dementia outcomes in UK: a prospective cohort study
Source: BMJ Open. 2022 Jun 15;12(6):e060931. doi: 10.1136/bmjopen-2022-060931 (PMC9204445; doi:10.1136/bmjopen-2022-060931)
Supplement: Author's manuscript [file bmjopen-2022-060931.draft_revisions.pdf]

BMJ Open is committed to open peer review. As part of this commitment we make the peer review history of every article we publish publicly available.

When an article is published we post the peer reviewers' comments and the authors' responses online. We also post the versions of the paper that were used during peer review. These are the versions that the peer review comments apply to.

The versions of the paper that follow are the versions that were submitted during the peer review process. They are not the versions of record or the final published versions. They should not be cited or distributed as the published version of this manuscript.

BMJ Open is an open access journal and the full, final, typeset and author-corrected version of record of the manuscript is available on our site with no access controls, subscription charges or pay-per-view fees (http://bmjopen.bmj.com).

If you have any questions on BMJ Open's open peer review process please email info.bmjopen@bmj.com

BMJ Open

Strengths and limitations of routinely collected records for dementia outcomes in United Kingdom (UK). Examination of mortality and National Health Service (NHS) data sources to ascertain dementia in a British cohort.

Journal:	*BMJ Open*
Manuscript ID	bmjopen-2022-060931
Article Type:	Original research
Date Submitted by the Author:	10-Jan-2022
Complete List of Authors:	Hayat, Shabina; University of Cambridge, Department of Psychiatry<br>Luben, Robert ; NIHR Moorfields Biomedical Research Centre; MRC Epidemiology Unit<br>Khaw, Kay-Tee; University of Cambridge; MRC Epidemiology Unit<br>Wareham, Nicholas; MRC Epidemiology Unit<br>Brayne, Carol; University of Cambridge, Department of Psychiatry
Keywords:	Dementia < NEUROLOGY, PUBLIC HEALTH, EPIDEMIOLOGY

SCHOLARONE™
Manuscripts

I, the Submitting Author has the right to grant and does grant on behalf of all authors of the Work (as defined in the below author licence), an exclusive licence and/or a non-exclusive licence for contributions from authors who are: i) UK Crown employees; ii) where BMJ has agreed a CC-BY licence shall apply, and/or iii) in accordance with the terms applicable for US Federal Government officers or employees acting as part of their official duties; on a worldwide, perpetual, irrevocable, royalty-free basis to BMJ Publishing Group Ltd ("BMJ") its licensees and where the relevant Journal is co-owned by BMJ to the co-owners of the Journal, to publish the Work in this journal and any other BMJ products and to exploit all rights, as set out in our licence.

The Submitting Author accepts and understands that any supply made under these terms is made by BMJ to the Submitting Author unless you are acting as an employee on behalf of your employer or a postgraduate student of an affiliated institution which is paying any applicable article publishing charge ("APC") for Open Access articles. Where the Submitting Author wishes to make the Work available on an Open Access basis (and intends to pay the relevant APC), the terms of reuse of such Open Access shall be governed by a Creative Commons licence – details of these licences and which Creative Commons licence will apply to this Work are set out in our licence referred to above.

Other than as permitted in any relevant BMJ Author's Self Archiving Policies, I confirm this Work has not been accepted for publication elsewhere, is not being considered for publication elsewhere and does not duplicate material already published. I confirm all authors consent to publication of this Work and authorise the granting of this licence.

For peer review only

Strengths and limitations of routinely collected records for dementia outcomes in United Kingdom (UK). Examination of mortality and National Health Service (NHS) data sources to ascertain dementia in a British cohort.

Authors: Shabina A Hayat,[1] Robert Luben,[2, 3] Kay-Tee Khaw, Nick Wareham,[3] Carol Brayne.[1]

1. Department of Psychiatry, Cambridge Public Health, University of Cambridge, Cambridge, UK
2. NIHR Biomedical Research Centre at Moorfields Eye Hospital & UCL Institute of Ophthalmology, London, UK
3. MRC Epidemiology Unit, University of Cambridge, Cambridge, UK

Corresponding author:
Dr Shabina Hayat (Sah63@medschl.cam.ac.uk)
Department of Psychiatry, Cambridge Public Health
University of Cambridge,
Cambridge, UK

Word Count:

Abstract: 284 words

Main Text: 4076 words

For peer review only

Abstract

Objectives: To evaluate the characteristics of individuals recorded as having a dementia diagnosis in different routinely collected records and to examine the extent of overlap of dementia coding across data sources. Also, to present comparisons of secondary and primary care records providing value for researchers using routinely collected records for dementia outcome capture.

Study Design: A prospective cohort study

Setting and Participants: A cohort of 25,639 men and women in Norfolk, aged 40-79 years at recruitment (1993-1997) followed until 2018 linked to routinely collected to identify dementia cases. Data sources include mortality from death certification and National Health Service (NHS) hospital or secondary care records. Primary care records for a subset of the cohort were also reviewed.

Primary outcome measure: Diagnosis of Dementia (any-cause)

Results: Over two thousand participants (n= 2635 individuals) were found to have a dementia diagnosis recorded in one or more of the data sources examined. Limited concordance was observed across the secondary care data sources. We also observed discrepancies with primary care records for the sub-set and report on potential linkage-related selection bias

Conclusions: Use of different types of record linkage from varying parts of the UK's health system reveals differences in recorded dementia diagnosis, indicating that dementia can be identified to varying extents in different parts of the NHS system. However, there is considerable variation, and limited overlap in those identified. We present potential selection biases that might occur depending on whether cause of death, or primary and secondary care data sources are used. With the expansion of using routinely collected health data, researchers must be aware of these potential biases and inaccuracies, reporting carefully on the likely extent of limitations and challenges of the data sources they use.

For peer review only

Strengths and Limitations

- With no current 'gold standard' for dementia case ascertainment, this study explores the reliability of dementia diagnosis from a range of data sources in a cohort of 25,639 participants
- This study confirms that there is limited concordance for dementia across data sources
- The absence of a dementia diagnosis code was considered to be absence of the dementia, which is not necessarily the case
- In UK, record linkage with health care data is sufficient to identify dementia cases for epidemiologic analyses of risk factors, although these are less reliable for calculating incidence and prevalence rates

For peer review only - http://bmjopen.bmj.com/site/about/guidelines.xhtml

For peer review only

For peer review only - http://bmjopen.bmj.com/site/about/guidelines.xhtml

Introduction

Routinely collected health records are powerful tools that have been increasingly used for research in the last decade. [1] In the United Kingdom (UK), the potential value of using health care data led to the launch of the Farr Institute of Health Informatics in 2013, and its successor Health Data Research UK (HDR-UK) in 2018 with the vision to improve population health, address health inequalities, and to drive efficient service provision. [2] Linkage to medical records have also been increasingly used to examine dementia causes and outcomes. Accuracy of medical records, of whatever type, tends to be assumed in the literature. However there is no 'gold standard' for dementia case ascertainment . [3] Researchers have employed a range of methods to examine databases to find reported dementia diagnoses. Whilst the availability of data provides opportunities for powerful and efficient research, it also presents some challenges and limitations, in particular, in relation to dementia-related outcomes that merit further exploration.[3,4]

Epidemiological studies depend on accurate, reliable and relevant information from data sources. However, there is substantial heterogeneity in the methods for case-ascertainment in dementia literature, [5] with studies applying different criteria. Compared with population based surveys, dementia is still known to be underreported in primary health care records, with individuals having manifest signs of dementia but no record of a dementia diagnosis, although this 'gap' is being closed more recently. [6] Dementia ascertainment is complex in primary and secondary care settings [7,8] as well on death certification. [9] There are many influences on whether dementia is likely to be recorded in these settings. Variations in dementia reporting have been noted across regions in UK [10,11]. Data sources are prone to inconsistency, misclassification and are influenced by change in dementia practice and policy, [10] such as those summarised in Box 1 below.

In the UK, the National Health Service (NHS) holds data on all hospital admissions by condition, allowing dementia ascertainment from routine data sources. To date, mortality and Hospital Episode Statistics (HES) data have been the main sources of health records for epidemiological studies. [3,4,12] These data are coded using the International Classification of Diseases (ICD) which are mainly diagnostic codes. Using these sources alone seriously underestimates the number of dementia cases, as most dementia patients will not require hospitalisation for their dementia nor will they have dementia as a cause of death recorded on their death certificate. [12,13]

For peer review only - http://bmjopen.bmj.com/site/about/guidelines.xhtml

General practitioners (GP) maintain primary care records, which include information, not only for diagnoses, but also for administrative purposes, including details of specialist referrals such as to memory clinics. Diagnosis of dementia is usually initiated in a primary care setting, based on patient symptoms or caregivers' concerns. [14] GP records, in theory, should be a more complete source for dementia case ascertainment. The coding system used in primary care uses Read Codes, is separate from the ICD system used in secondary care and death registration. Codes for dementia, are numerous, complex and prone to coding error and misclassification. [7] Another data source for dementia, which seems to have had less use in research, is the national mental healthcare dataset (MHDS) which contain record-level data about individuals in contact with mental health services. The use of multiple systems across different settings and unfamiliar codes adds to the confusion of dementia case ascertainment. [7] The impact of incomplete or inaccurate recording of dementia can affect analysis and interpretation of results. [15]. Furthermore, databases are influenced by the point data are collected in the patient care pathway [4] and may not be representative of the full population of people living with dementia, thus affecting generalisability of the findings. [16]

The objective of this study was to describe the procedures for a better understanding of the characteristics of individuals recorded as having a dementia diagnosis in different health care records and examine the extent of overlap of dementia coding across data sources. We present novel insight into the MHDS and compare secondary and primary care records providing value for others relying on these types of data sources for outcome capture.

Ethics

EPIC-Norfolk has the permissions (Section 251 and where possible, explicit signed consent given by participants attending health examinations) to follow up through medical record linkage. Procedures for this study have been approved by NHS Research Ethics Committee (NRES REC Ref: 98CN01). The Regulatory approvals from the Confidentiality Advisory group (CAG) can be found at:

Section 251 Application number 059; https://www.hra.nhs.uk/planning-and-improving-research/application-summaries/confidentiality-advisory-group-registers/).

For the GP dementia case ascertainment and validation sub-study – we also obtained approval from Norfolk & Suffolk Primary & Community Care Research Office for access to GP Practices.

For peer review only - http://bmjopen.bmj.com/site/about/guidelines.xhtml

Patient and public involvement

The EPIC-Norfolk study actively promotes greater participant involvement in research. The EPIC-Norfolk Participant Advisory Panel (EPAP) is a consultative forum providing participant perspective on how research is prioritised, planned, conducted, communicated and used. EPAP is viewed as a partnership between EPIC-Norfolk researchers and participants. Further details on EPAP can be found here:

https://www.epic-norfolk.org.uk/for-participants/epap/

For peer review only

For peer review only - http://bmjopen.bmj.com/site/about/guidelines.xhtml

Box 1: Changes in policy for public and practitioners which may have increased awareness of dementia and recording of cases in health records

2006: Introduction of dementia to Quality of Framework (QOF) registers. QOF is an incentivising primary care scheme that rewards GP Practices for the provision of quality care and helps to standardise improvements in the delivery of primary medical services. [17] Participation in QOF is voluntary.

2009: The 2009 National Dementia Strategy was introduced to increase the public and professional awareness and understanding of dementia. Also, the dementia prevalence indicator to the Quality Outcomes Framework was introduced in primary care

2011: Changes to coding practice. For mortality coding, deaths previously coded with an underlying cause of unspecified cerebrovascular disease were reclassified as vascular dementia

2012: The Dementia Challenge was launched in March 2012 by Prime Minister, David Cameron. This programme superseded the national strategy from 2009- focus was on three main areas: bringing about improvements in health and care, creating dementia friendly communities and improving research

2013: The Dementia Commissioning for Quality and Innovation (CQUIN) was introduced (to encourage practitioners to identify those who potentially have dementia in a secondary care setting, most particularly applied to those over 75 years with unplanned hospital admissions

2014: Mortality coding revised - dementia coded as the underlying cause of death

2015: Prime Minister launched his Challenge on Dementia 2020, which set out to build on the achievements of the Prime Minister's Challenge on Dementia 2012-2015

Methods

Study Population and data collection

Participants were recruited to the European Prospective Investigation of Cancer (EPIC) in Norfolk. Details of this study have been previously described. [18] Briefly, 30,445 men and women (40–79 years) residents of Norfolk, England, were recruited to the study via their general practices. Of these 25,639 attended the baseline health examination between 1993 and 1997. Subsequent follow-ups have involved self-report of health and lifestyle postal questionnaires and further health examinations. [19] Virtually complete follow-up for cohort for mortality and hospital admissions in EPIC-Norfolk has been established via linkage to routinely collected National Health Service (NHS) databases in England (Hospital Episode Statistics, HES) and for mortality data for all participants using their unique NHS number and date of birth.

For peer review only - http://bmjopen.bmj.com/site/about/guidelines.xhtml

Dementia ascertainment and diagnostic codes in hospital and death records and mental healthcare data

Data linkage to the EPIC-Norfolk cohort for NHS and mortality data was carried out by NHS Digital, a statutory body in England, permitted to receive identifiable patient data for linkage. The linked hospital records contain coded diagnostic information for all inpatient and day-case admissions. [20] We also obtained national mental healthcare data, which contain record-level data about individuals in contact with mental health services including memory clinics.

Participants with incident dementia were defined as those free of dementia at the time of enrolment to the study and then identified with a dementia diagnosis recorded in medical records subsequently. Individuals were followed from the date of consent, until the earliest date of a dementia diagnosis or date of death in or after 1996 when formal follow up by health records began in EPIC-Norfolk. Dementia from HES records, death certificate or the MHDS was defined as any of the ICD-10 codes as listed in Supplementary Table S1. To maximise the outcome data, we included any-cause dementia, the sub types of dementia were not analysed separately. Participants with no reports of death or dementia were censored on 31st December 2018. Figure 1 is a diagrammatic representation of selection of participants and record linkage in the cohort.

Dementia case ascertainment through GP records

For a subset, we compared the NHS Digital datasets of secondary care to GP records. The protocol was based on a ‘clean up’ exercise [7] recommended by clinical commissioning groups (NHS bodies that commission services for defined populations) to improve dementia diagnosis in general practices. [21] We employed Read codes (Supplementary Table S1) identified as most commonly used for a dementia diagnoses. [7] Twenty-six collaborating GP practices were invited to participate. Those practices that agreed to take part in this exercise were provided with a detailed protocol and a list of patients on their register who were also EPIC-Norfolk participants. Each practice ran searches to generate lists of these respondents to establish which had a dementia diagnosis code, or were prescribed dementia defining medication, Donepezil, Galantamine, Rivastigmine or Memantine as identified by the relevant Read code (Supplementary Table S1).

Data were returned via a secure transfer system. Practices were also asked to complete a simple questionnaire. The full protocol and sample questionnaire are available on request. Practices were advised to take advantage of this exercise by comparing results of the search against their Quality of

For peer review only - http://bmjopen.bmj.com/site/about/guidelines.xhtml

Framework (QOF) [10] dementia register to check for discrepancies to improve their dementia diagnosis rates. [7]

In the second phase, cases identified from GP records were cross-checked with the NHS Digital data, and the level of agreement between the data sources was examined. Participants with a dementia diagnosis in secondary care, but not indicated in the GP data were further reviewed by researchers in the third and final confirmation phase. Initial case ascertainment from GPs was conducted between 2016-2017 and NHS Digital data were up to 31st March 2018, with case confirmation in GP records up to 20th December 2018.

Statistical Analyses

Definite dementia cases were defined as having one or more of the ICD-10, or (for GP records) Read code, for dementia. Age and sex-specific incidence rates for all-cause dementia and mortality rates were calculated for the entire cohort. The number and proportion of participants with a diagnosis of dementia from the three NHS Digital data sources separately and combined were examined. We also examined differences in socio-demographic characteristics (age at recruitment, sex, education and social class) of dementia cases across the three NHS Digital datasets. Education (the highest level attained) and social class were obtained from the baseline questionnaire. Education was categorised into three groups (i) No qualification (not completing school up to the age of 16), (ii) Completion of school up to the age of 16 or up to the age of 18 and finally (iii) those obtaining an education to graduate level (those who obtained a degree or equivalent) or above.

Social class was classified according to the Registrar General's occupation-based classification scheme into five main categories. [22] Social class I consists of professionals, class II includes managerial and technical occupations, class III is subdivided into non-manual and manual skilled workers (III non-manual and III manual), class IV consists of partly skilled workers, and class V comprises unskilled manual workers. We used Cox proportional-hazards models to compare the association of socio-demographic factors and risk of a dementia diagnosis across data sources, with mutual adjustment of co-variates in the model. Statistical analyses were performed using SPSS version 25.0 (IBM Corp., Armonk, NY, USA).

Figure 1: Flow diagram of selection of EPIC-Norfolk participants for record linkage

For peer review only - http://bmjopen.bmj.com/site/about/guidelines.xhtml

Figure 1: HERE

Results

There were 2635 cases of dementia identified from the cohort of 25,639 individuals at the censor date of 31st December 2018 after 25.8 years of follow-up. The youngest age of entry to the study at baseline was just below 40 and the oldest age of the participant at the censor date was 101 years. Out of the 2298 individuals with data on age of diagnosis, the minimum age of diagnosis was 54 years and maximum was 99 years. Figure 2 shows the relationship between age and dementia diagnosis. Table 1 shows the sex and age-specific cumulative incidence of dementia and deaths in the cohort. Increasing age was associated with increasing rates of dementia and death. This table reflects the higher mortality in men which results in higher absolute numbers and of dementia cases in women.

Figure 2: Scatter plot of age of diagnosis by age at baseline in the EPIC-Norfolk cohort

Figure 2: HERE

For peer review only - http://bmjopen.bmj.com/site/about/guidelines.xhtml

Table 1: Age and sex specific proportions of dementia and death in EPIC-Norfolk from 1996 until 31st December 2018 using all 3 secondary care data sources provided by NHS Digital for each age group by gender at baseline.

Age Band at baseline	Median age	Freq (N)	% with dementia recorded at any time in follow-up (N)	% Died (N)	P-Value
**Men (N=11 607)**					
<=59 Years	51.6	5915	3.2 (190)	17.4 (1028)	
60-64 Years	62.5	1848	12.3 (228)	46.7 (863)	
65-69 Years	67.5	1890	15.6 (294)	72.0 (1361)	<0.001
70-74 Years	72.5	1579	17.3 (273)	89.9(1420)	
>75 years	75.7	375	14.9 (56)	95.7 (359)	
**Women (N=14032)**					
<=59 Years	51.3	7656	3.1 (234)	11.9 (909)	
60-64 Years	62.6	2118	15.0 (318)	36.9 (782)	
65-69 Years	67.4	2103	23.2 (488)	55.4 (1166)	<0.001
70-74 Years	72.4	1766	27.5 (486)	80.4 (1419)	
>75 years	75.8	389	28.8 (112)	90.7 (353)	
**ALL (N=25 639)**					
<=59 Years	51.5	13571	3.0 (412)	14.3 (1937)	
60-64 Years	62.6	3966	13.5 (536)	41.5 (1645)	
65-69 Years	67.5	3993	19.3 (769)	63.3 (2527)	<0.001
70-74 Years	72.5	3345	22.8 (750)	84.7 (2839)	
>75 years	75.7	764	22.0 (168)	93.2 712)	

P values by Chi sq. for proportion of individuals with dementia in each age category. Results shown for men and women separately and combined (ALL)

On comparing characteristics across the different data sources, there were no differences in terms of sex and socio-demographic profiles (Table 2), other than those with dementia recorded from the mortality dataset were slightly older. Most recorded diagnoses of dementia were found in HES, followed by cause of death, with the least number identified from the MHDS. The distribution of dementia diagnoses across the three NHS Digital data sources is shown in Figure 3. This figure shows very little overlap. In terms of socio-demographic factors predicting presence of a recorded diagnosis of dementia, associations were similar across the three data sources (Table 3). Age was a stronger predictor in the mortality data and weakest in the mental health data. Having qualifications was associated with a lower risk of future recorded diagnosis of dementia, this was observed for mortality and HES data but not for the MHDS.

For peer review only

For peer review only - http://bmjopen.bmj.com/site/about/guidelines.xhtml

Figure 3: Distribution of cases (N=2635) across the three main data sources from NHS Digital followed up until 31st December 2018

Figure 3: HERE

For peer review only

For peer review only - http://bmjopen.bmj.com/site/about/guidelines.xhtml

Table 2: Comparison of characteristics of 'definite dementia' cases identified from the three data sources separately and combined.

	All 3 data sources HES/MHDS/Mortality	HES	MHDS	Mortality	P-value
	Definite dementia N=2635	Definite dementia N=2157	Definite dementia N=727	Definite dementia N=1276	
**Socio-demographic Mean (SD)**					
**Age at Baseline**	66.7 (6.5)	66.9 (6.3)	64.1 (6.5)	67.9 (5.9)	<0.001
**Age at Diagnosis**	83.8 (6.5)	83.7 (6.5)	82.7 (6.8)	84.6 (6.4)	<0.001
**Sex, % women (n)**	60.5 (1594)	59.8 (1290)	59.7 (434)	60.5 (772)	0.9
**Education, % (n)**					
No qualifications	48.4 (1274)	49.1 (1057)	45.3 (329)	50.4 (641)	
O/ A level Standard	42.6 (1120)	41.9 (903)	45.3 (329)	41.4 (527)	0.5
Graduate Level	9.0 (238)	9.0 (194)	9.4 (68)	8.2 (105)	
**Social Class, % (n)**					
Professional	6.3 (162)	6.3 (132)	5.6 (42)	6.0 (74)	
Managerial	33.9 (866)	33.6 (702)	32.0 (240)	34.0 (421)	
Skilled Non-Manual	20.5 (523)	20.5 (428)	21.1 (158)	21.7 (268)	
Skilled Manual	21.3 (544)	21.2 (442)	21.4 (160)	20.5 (253)	0.7
Semi-Skilled	14.2 (362)	14.1 (295)	17.2 (129)	13.6 (168)	
Non-Skilled	3.8 (98)	4.2 (88)	2.7 (20)	4.3 (53)	

Abbreviations: HES, Hospital Episode Statistics, MHDS, Mental Health Dataset, N, number; O/A, Ordinary or Advanced level; SD, Standard deviation

For peer review only

For peer review only - http://bmjopen.bmj.com/site/about/guidelines.xhtml

Table 3: Association of sociodemographic factors and dementia in the EPIC-Norfolk Cohort in the three data sources combined and individually, mutually adjusted for age, sex, education and social class.

	All 3 data sources (Dementia=2555)			HES only (Dementia=2087)			MHDS only (Dementia=715)			Mortality only (Dementia=1229)		
Factor	HR	95% CI	P-Value	HR	95% CI	P-Value	HR	95% CI	P-Value	HR	95% CI	P-Value
Age per 5 years	2.19	(2.13, 2.26)	<0.001	2.23	(2.16, 2.31)	<0.001	1.80	(1.71, 1.89)	<0.001	2.57	(2.46, 2.69)	<0.001
Sex (Men)	0.98	(0.91, 1.06)	0.6	1.01	(0.92, 1.10)	0.9	1.03	(0.88, 1.20)	0.7	1.08	(0.96, 1.21)	0.2
Education (Any qualifications)	0.87	(0.80, 0.95)	0.002	0.87	(0.79, 0.95)	0.002	0.91	(0.78, 1.07)	0.2	0.84	(0.74, 0.94)	0.003
Social Class (Non-manual)	0.99	(0.91, 1.08)	0.8	0.98	(0.89, 1.07)	0.7	0.93	(0.79, 1.09)	0.4	1.03	(0.91, 1.16)	0.7

Abbreviations: CI, Confidence Interval; HES, Hospital Episode Statistics, HR, Hazard ratio; MHDS, Mental Health Dataset

For peer review only

The MHDS consisted of mainly administrative data such as mental health reviews, care programmes and pathways that include contacts with mental health care professionals (both in hospitals and in outpatient memory clinics and the community) as well as diagnostics and treatment codes. The service-level breakdown of the mental health data was not applicable here, as there was little additional diagnostic information. The latest release in 2017-2018 appeared to be the most complex, covering mental healthcare more comprehensively and containing diagnostic ICD10 codes that had been limited in the previous years.

For the sub-study involving validation with GP records, out of the 26 practices that were contacted, 14 agreed to participate, 6 declined and 6 did not respond. Of the 14 that agreed, 8 practices completed the questionnaire and 9 provided data. There were no criteria for selecting a practice for this validation, other than they were practices that were collaborating with the study. There was a mix of rural and city practices, classified as urban or town and fringe areas, [23] although the majority of the practices ultimately submitting data were city-based. Different types of staff carried out the linkage with their records, ranging from practice managers, IT managers, research nurses to a health care assistant.

In this sub-population, 4.4% (209 of the 4668 participants) were dementia cases identified from HES, mental health data, mortality and the GP records compared to 10.1% (2635 dementia cases out of 25,635 participants) in the rest of the cohort. The individuals of the sub-study registered with the responding practices were younger, more likely to be women, with higher education and social class when compared to the rest of the cohort (Supplementary Table S2). Of the 209 respondents with a recorded diagnosis of dementia, 57 were found in both secondary and primary care data and 87 were in primary care records only. In this small study, almost all the practices were using Read codes and all reported their dementia cases to be confirmed through secondary referral to memory clinics. Five out of the eight practices reported that some dementia cases were not previously identified and so were missing on their QOF registers. The summarised responses to the questionnaire from GPs are given in Supplementary Information (Table S3). Participants who were prescribed one of the four dementia drugs (N=57), also had a Read code of a definite dementia diagnosis. There were no cases identified from drugs alone.

There were 65 participants who had a definite dementia diagnosis in the secondary care records, but not in their GP records. For most of these (51 cases), the reason for absence from GP systems was because the participant had died, and the dementia diagnoses had come from mortality records. For

the remaining (N=14), 3 participants had Mild Cognitive Impairment (MCI) or probable dementia and for 10 participants, we were unable to confirm diagnosis. This could be because they had died recently, or had moved to another practice, in which case their records would no longer be available to the GP. One patient had no indication of any dementia or dementia related condition in their record. Of the 57 cases that were in both the secondary and primary data sources, 30 cases were obtained from the initial GP data extract, with 27 missed by the practices. These cases were ascertained with further review of individual records by the researchers and would have been missed if the extra confirmation phase had not been a part of the study design. Distribution of dementia cases across primary and secondary care datasets are shown in Figure 4.

Figure 4: Distribution of cases (N=209) identified from primary and secondary care in sub-population of EPIC-Norfolk participants (N=4668).

Figure 4: HERE

For peer review only

Discussion

Medical records allow virtually complete follow-up for dementia research, however there are limitations in terms of accuracy, completeness and underestimation of cases across data sources. [3,4,8,24,25] This study confirms, that although there is some concordance for dementia across data sources, this is rather limited. Hospital records yielded the greatest number of cases, followed by death certification with the least number identified from the MHDS.

Limitations and strengths

As in other studies, [3,12,26] we interpreted the absence of a dementia diagnosis code as absence of the dementia; this is clearly not the case. Even though using medical records is less sensitive to an in-study algorithmic approach to dementia diagnosis, specificity in all these data sources is likely to be high, as a clinical diagnosis, particularly in primary care records is usually made after referral to a specialist. [27] Another weakness is that we did not inspect medical records for the entire cohort, and so likely to have missed cases, reducing sensitivity. Furthermore, we would have missed milder cases as we only included definite dementia diagnosis, other than when confirming cases, where we searched for milder impairment. Misclassification will have some impact on associations, although, the level of importance of this will depend on the purpose of the study. Under-ascertainment is inevitable and should be considered when making prevalence estimations.

Another limitation is that we did not account for the competing risk of death, which will be high in this ageing population. However, the proportion of deaths by age in men and women were presented. Individuals reaching the end of life with other comorbidities may die before any diagnosis of dementia. [28] To account for the competing risk for death, death must be a discrete event from dementia, and given that we included dementia from mortality records, this overlap does not allow for competing risk of death to be estimated here. However, in the Cox regression models, these individuals would have been censored, and once censored, these individuals are no longer at risk.

Using simple socio-demographic factors to examine associations with future risk of a definite dementia diagnosis, we observed small differences, across the main two data sources, HES and mortality, with more difference observed in the MHDS. The lower risk of dementia diagnosis for those with qualifications observed for mortality and HES data but not for the MHDS, could be due the smaller numbers in the MHDS, or reflecting that there was no difference in terms of education as to who accesses mental health services. For the socio-demographic factors examined here, we had very little missing data, however for more complex factors where there is likely to be more missing data, there

For peer review only

is the potential for these differences to be greater. Such factors and unrecorded dementia cases in a data sources could yield different study results. Studies have highlighted regional variation in rates of diagnosis and reliability of existing data. [3] We have demonstrated that this heterogeneity exists even in a single geographical region.

We were also able to draw on the relatively new MHDS from NHS Digital, as yet not widely used in research or described in detail. All activity relating to patients of any age who receive care for a suspected or diagnosed mental health. learning disability, or neurodevelopmental conditions is within scope of this dataset. This is a further source of clinical and operational data in the NHS that can be used for purposes other than direct patient care, including research. Although the MHDS covers mental healthcare more comprehensively than the other sources, it is limited as most of the clinical information is not coded, but recorded in text, thus, not lending itself so easily large-scale analyses. [9]

We found MHDS to be complex, with information on individuals (from referral to final discharge) on contact with secondary mental health services. Each subsequent release of the mental health data was wider in scope than the previous version. The data in the final year of follow-up gave the greatest number of dementia cases. The sharp increase in diagnosis codes shows how important understanding the policy and practice context is to assess the influences and potential biases inherent in use of routine data sources. Additional reasons include changes in coding practices in mental health services, or the way these data are extracted to include diagnoses as well as service codes. The MHDS has far greater potential to provide more complete estimates of diagnosed dementia in the population in the UK. This dataset will need further reviewing for future work.

It is currently not possible to examine primary care records via databases such as the Clinical Practice Research Datalink (CPRD) for this cohort. As we had to rely on GP practices to extract the data, we were restricted, for practical reasons, to link to a subset only. The protocol was time and labour intensive. We did not observe the same level of concordance with hospital records as shown in other larger cohort studies. [12,26] Some of this discrepancy was due to patients having died as GP records for people who die are moved swiftly to NHS archives. This study also identified lack of technical capacity as a potential source for under-ascertainment with almost half the cases were initially missed by the practice. This further reveals the scale of the missed outcomes if using currently available routine records given that known cases that are not reflected in extraction of routine records This will

For peer review only

influence studies using databases such as CPRD. [29] These findings the importance of extending further training and knowledge of dementia coding in primary care.

As with the UK Biobank study, [26] we also found significant proportion of dementia cases in primary care records, that had not been found in hospital or mortality records. In EPIC-Norfolk this figure was 42% (87 out of the 209 dementia cases identified) compared to 52% reported in UK Biobank. The lower proportion in our study reflects the older age of our cohort, and so more likely to appear in hospital and mortality records than the participants from UK Biobank. UK Biobank included participants from Scotland where data quality is high and centralisation of data, linkable health service datasets make data more accessible to research. This position is not currently shared by the other countries of UK, although may change in the near future with the establishment of HDRUK. [2] Another large cohort study, the Million Women Study, found NHS hospital admission data to agree with primary care records, [12] This study only consisted of women, and also like UK Biobank, participants were younger.

The study in the sub-population of the EPIC-Norfolk cohort with GP records has highlighted several key issues. The first is the potential level of 'missingness,' [30] if we had relied on the initial data extract from practices, we would have missed just under half the dementia cases, even though the diagnosis was present in the health records. Secondly, it is clear from the characteristics and dementia incidence that this sub-population was different from the overall cohort. Although, there were no specific criteria set in approaching practices, the practices that responded were mainly city practices, and the patient-base in these practices was younger, more educated and of higher social class. It is likely that the more research active practices responded. This bias is clearly reflected in the dementia rates, which was 10% in the EPIC-Norfolk cohort overall, compared to 4% in the sub-set from the nine GP practices taking part. Databases such as CPRD are extremely powerful data sources, but are not representative of all practices in the UK based on geography and size. [30] HES and mortality also rely on data provided by practices. Our findings show the extent to which practice selection impact on dementia case ascertainment and thus on measured rates. Therefore, these data should be used and interpreted with caution.

Recently there have been changes to coding within primary care with the introduction of new codes SNOMED CT. [31] This new coding system will replace Read codes and eventually also be used in secondary care providing clarity and consistency. However, the implementation of this will take several years and the impact of this is unknown. There is also potential in the mental health data that

For peer review only

includes other service-related codes that could be utilised for further insight into the level of cognitive impairment in the community. There are several administrative codes that relate to low, moderate and severe cognitive impairment. This information could be used to supplement the diagnostic information and could be useful in ascertaining milder forms of impairment and dementia.

The study suggests that in UK, record linkage with hospital admission, mortality and primary care data is sufficient to identify dementia cases for epidemiologic analyses of risk factors. The reliability of these data for incidence and prevalence rates is more challenging due to variability in ascertainment and diagnostic criteria which may differ over time and in different populations. Using a single data source would clearly underestimate dementia outcomes, and so drawing from multiple sources is the best approach to maximize dementia ascertainment from routinely collected health records. [3,16] Researchers must be fully aware of the strengths and limitations of the data sources they use, identifying the potential sources of bias [32] and be transparent in reporting on how these reflect on the accuracy of their findings.

Acknowledgments:

The authors would like to thank all study participants, general practitioners and the EPIC Norfolk study team for their contribution to this work. We would also like to thank Nichola Dalzell who played a huge part in the data collection phase of the study.

Funding

The EPIC-Norfolk study (DOI 10.22025/2019.10.105.00004) has received funding from the Medical Research Council (MR/N003284/1 MC-UU_12015/1 and MC_UU_00006/1), Cancer Research UK (C864/A14136) and NIHR https://www.nihr.ac.uk (Ref: NF-SI-0616-10090 to [CB]).

Competing Interests: None

References

1 Casey JA, Schwartz BS, Stewart WF, *et al.* Using Electronic Health Records for Population Health Research: A Review of Methods and Applications. *Annu Rev Public Health* 2016;**37**:61–81. doi:10.1146/annurev-publhealth-032315-021353

2 Ford E, Boyd A, Bowles JKF, *et al.* Our data, our society, our health: A vision for inclusive and transparent health data science in the United Kingdom and beyond. *Learn Heal Syst* 2019;**3**:e10191. doi:10.1002/lrh2.10191

3 Sibbett RA, Russ TC, Deary IJ, *et al.* Dementia ascertainment using existing data in UK longitudinal and cohort studies: a systematic review of methodology. *BMC Psychiatry* 2017;**17**:239. doi:10.1186/s12888-017-1401-4

4 Wilkinson T, Ly A, Schnier C, *et al.* Identifying dementia cases with routinely collected health data: A systematic review. *Alzheimer's Dement* 2018;**14**:1038–51. doi:10.1016/J.JALZ.2018.02.016

5 Global Burden of Diseases (GBD) 2016 Dementia Collaborators. Global, regional, and national burden of Alzheimer's disease and other dementias, 1990-2016 a systematic analysis for the Global Burden of Disease Study 2016. *Lancet Neurol* 2019;**18**:88–106. doi:10.1016/S1474-4422(18)30403-4

6 Aldus CF, Arthur A, Dennington-Price A, *et al.* Undiagnosed dementia in primary care: a record linkage study. *Heal Serv Deliv Res* 2020;**8**:1–108. doi:10.3310/hsdr08200

7 Russell P, Banerjee S, Watt J, *et al.* Improving the identification of people with dementia in primary care: evaluation of the impact of primary care dementia coding guidance on identified prevalence. *BMJ Open* 2013;**3**:e004023. doi:10.1136/bmjopen-2013-004023

8 Sommerlad A, Perera G, Singh-Manoux A, *et al.* Accuracy of general hospital dementia diagnoses in England: Sensitivity, specificity, and predictors of diagnostic accuracy 2008–2016. *Alzheimer's Dement* 2018;**14**:933–43. doi:10.1016/j.jalz.2018.02.012

9 Perera G, Broadbent M, Callard F, *et al.* Cohort profile of the South London and Maudsley NHS Foundation Trust Biomedical Research Centre (SLaM BRC) Case Register: current status and recent enhancement of an Electronic Mental Health Record-derived data resource. *BMJ Open* 2016;**6**:e008721. doi:10.1136/bmjopen-2015-008721

10 Mukadam N, Livingston G, Rantell K, *et al.* Diagnostic rates and treatment of dementia before and after launch of a national dementia policy: An observational study using English national databases. *BMJ Open* 2014;**4**:1–8. doi:10.1136/bmjopen-2013-004119

11 Fox M, Fox C, Cruickshank W, *et al.* Understanding the dementia diagnosis gap in Norfolk and Suffolk: A survey of general practitioners. *Qual Prim Care* 2014;**22**:101–7.http://primarycare.imedpub.com/understanding-the-dementia-diagnosis-gap-in-norfolk-and-suffolk-a-survey-of-general-practitioners.pdf

12 Brown A, Kirichek O, Balkwill A, *et al.* Comparison of dementia recorded in routinely collected hospital admission data in England with dementia recorded in primary care. *Emerg Themes Epidemiol* 2016;**13**:1–9. doi:10.1186/s12982-016-0053-z

13 Romero JP, Benito-León J, Louis ED, *et al.* Under reporting of dementia deaths on death certificates: A systematic review of population-based cohort studies. *J Alzheimer's Dis* 2014;**41**:213–21. doi:10.3233/JAD-132765

14 Brayne C, Fox C. Dementia Screening in Primary Care. *Public Health* 2007;**298**:2409–11.

15 Weuve J, Proust-Lima C, Power MC, *et al.* Guidelines for reporting methodological challenges and evaluating potential bias in dementia research. 2015;**11**:1098–109. doi:10.1016/j.jalz.2015.06.1885

16 Davis KAS, Mueller C, Ashworth M, *et al.* What gets recorded, counts: dementia recording in primary care compared with a specialist database. *Age Ageing* 2021;**50**:2206–13. doi:10.1093/ageing/afab164

17 NHS England » Update on Quality Outcomes Framework changes for 2021/22. https://www.england.nhs.uk/publication/update-on-quality-outcomes-framework-changes-for-2021-22/ (accessed 15 Oct 2021).

18 Day N, Oakes S, Luben R, *et al.* EPIC-Norfolk: study design and characteristics of the cohort. European Prospective Investigation of Cancer. *Br J Cancer* 1999;**80**:95–103.

19 Hayat SA, Luben R, Keevil VL, *et al.* Cohort profile: A prospective cohort study of objective physical and cognitive capability and visual health in an ageing population of men and women in Norfolk (EPIC-Norfolk 3). *Int J Epidemiol* 2014;**43**:1063–72. doi:10.1093/ije/dyt086

20 Luben R, Hayat S, Wareham N, *et al.* Predicting admissions and time spent in hospital over a decade in a population-based record linkage study: the EPIC-Norfolk cohort. *BMJ Open* 2016;**6**. doi:10.1136/bmjopen-2015-009461

21 London Clinical Networks. "Coding clean-up ” Exercise Guidance to GPs to improve dementia coding , and raise diagnosis rates: A step by step approach. London, UK:

22 Shohaimi S, Welch A, Bingham S, *et al.* Residential area deprivation predicts fruit and vegetable consumption independently of individual educational level and occupational social class: a cross sectional population study in the Norfolk cohort of the European Prospective Investigation into Canc. *J Epidemiol Community Health* 2004;**58**:686–91. doi:10.1136/jech.2003.008490

23 British Household Panel Survey, waves 1-17 (1991-2008) User Documentation: Rural-Urban Indicator (URIND) Rural-Urban Indicator. 2008. http://doc.ukdataservice.ac.uk/doc/6032/mrdoc/pdf/6032userguide.pdf

24 Gao L, Calloway R, Zhao E, *et al.* Accuracy of death certification of dementia in population-based samples of older people: Analysis over time. *Age Ageing* 2018;**47**:589–94. doi:10.1093/ageing/afy068

25 Crowther GJE, Bennett MI, Holmes JD. How well are the diagnosis and symptoms of dementia recorded in older patients admitted to hospital? *Age Ageing* 2017;**46**:112–8. doi:10.1093/ageing/afw169

26 Wilkinson T, Schnier C, Bush K, *et al.* Identifying dementia outcomes in UK Biobank: a validation study of primary care, hospital admissions and mortality data. *Eur J Epidemiol* 2019;**34**:557–65. doi:10.1007/s10654-019-00499-1

27 Ford E, Greenslade N, Paudyal P, *et al.* Predicting dementia from primary care records: A systematic review and meta-analysis. Published Online First: 2018. doi:10.1371/journal.pone.0194735

28 Brayne C, Gao L, Dewey M, *et al.* Dementia before death in ageing societies - The promise of prevention and the reality. *PLoS Med* 2006;**3**:1922–30. doi:10.1371/journal.pmed.0030397

29 Donegan K, Fox N, Black N, *et al.* Trends in diagnosis and treatment for people with dementia in the UK from 2005 to 2015: a longitudinal retrospective cohort study. *Lancet Public Heal* 2017;**2**:e149–56. doi:10.1016/S2468-2667(17)30031-2

For peer review only

30 Herrett E, Gallagher AM, Bhaskaran K, *et al.* Data Resource Profile: Clinical Practice Research Datalink (CPRD). *Int J Epidemiol* 2015;**44**:827–36. doi:10.1093/ije/dyv098

31 NHS Digital. SNOMED CT: A clinical vocabulary for electronic health records. https://digital.nhs.uk/services/terminology-and-classifications/snomed-ct

32 Bohensky MA, Jolley D, Sundararajan V, *et al.* Data Linkage: A powerful research tool with potential problems. *BMC Health Serv Res* 2010;**10**:1–7. doi:10.1186/1472-6963-10-346

For peer review only

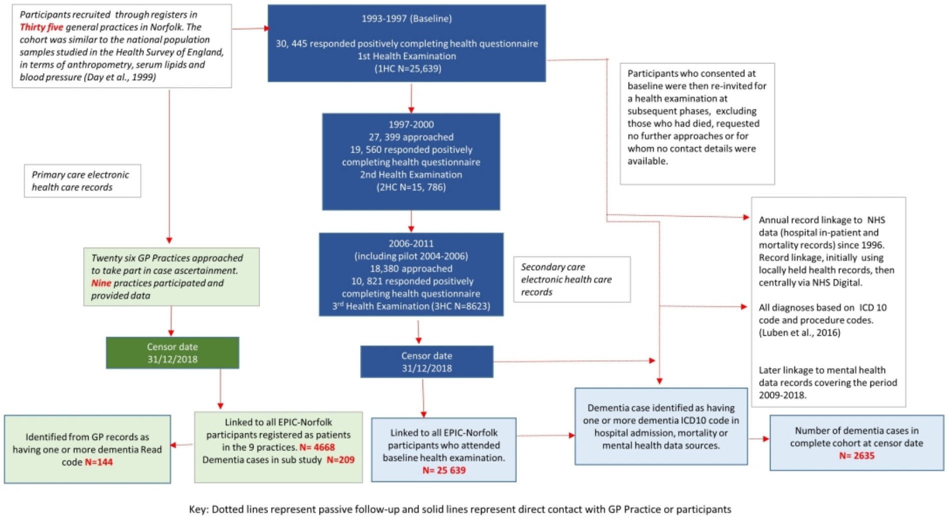

Figure 1: Flow diagram of selection of EPIC-Norfolk participants for record linkage

338x190mm (300 x 300 DPI)

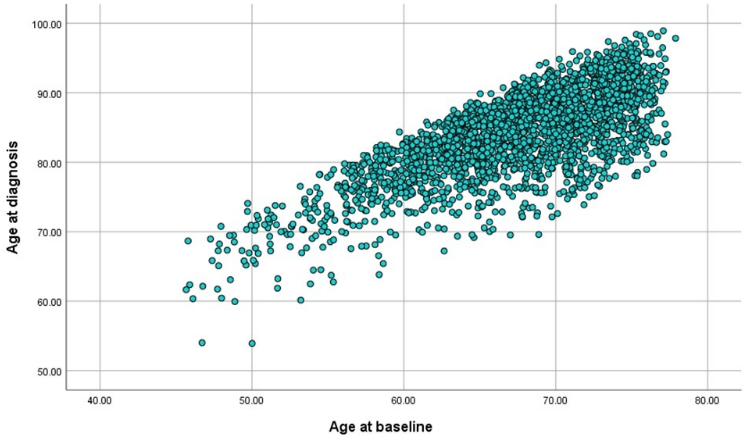

Scatter plot of age of diagnosis by age at baseline in the EPIC-Norfolk cohort

338x190mm (96 x 96 DPI)

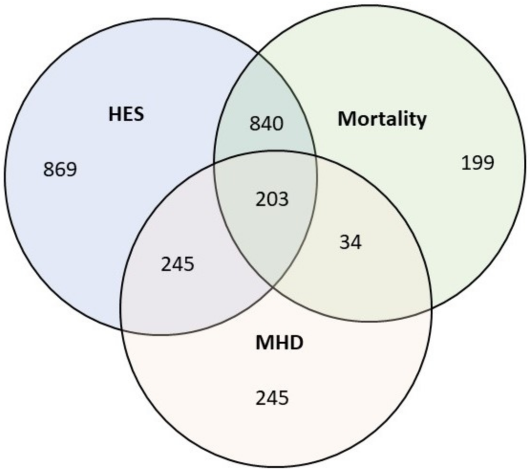

Figure 3: Distribution of cases (N=2635) across the three main data sources from NHS Digital fol-lowed up until 31st December 2018

277x177mm (96 x 96 DPI)

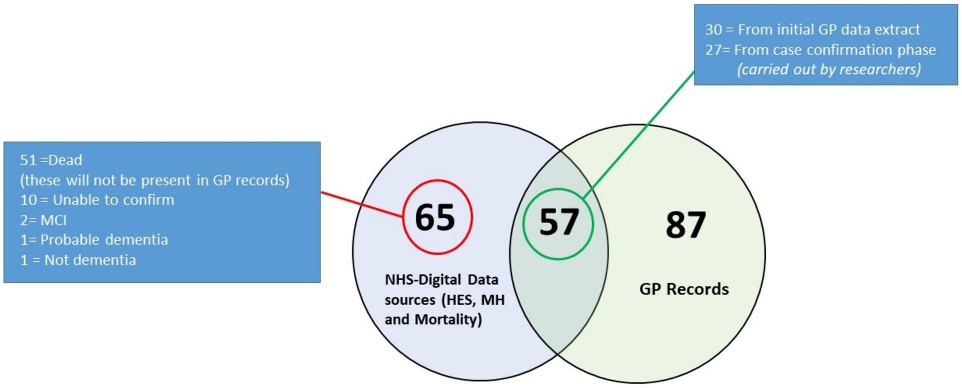

Figure 4: Distribution of cases (N=209) identified from primary and secondary care in sub-population of EPIC-Norfolk participants (N=4668).

338x190mm (96 x 96 DPI)

Supplementary Tables

Dementia outcomes from linkage to medical health records. An examination of mortality and National Health Service (NHS) data sources to ascertain dementia in a British cohort.

Table S1: Read and ICD 10 codes used in EPIC-Norfolk to search for 'Definite' dementia cases

Read codes	ctv3_codes	Read Code Description	ICD Code	ICD Description
**Eu00**	**Eu00**	Dementia in Alzheimer's disease	**F00**<br>**G30**	Dementia in Alzheimer's disease<br>Alzheimer's disease
**Eu000**		Dementia in Alzheimer's disease with early onset	**F00.0**<br>**G30.0**	Dementia in Alzheimer's disease with early onset
**Eu001**	**X0030**	Dementia in Alzheimer's disease with late onset	**F00.1**<br>**G30.1**	Dementia in Alzheimer disease with late onset
	**XaIKC**	Alzheimer's disease with late onset		
**Eu002**	**Eu002**	Dementia in Alzheimer's disease, atypical or mixed type	**F00.2**	Dementia in Alzheimer disease, atypical or mixed type
			**G30.8**	Other Alzheimer's disease
**F110**		Dementia in Alzheimer's disease, unspecified	**F00.9**<br>**G30.9**	Dementia in Alzheimer's disease, unspecified
**Eu01**	**XE1Xs**	Vascular dementia	**F01**	Vascular dementia
			**F01.0**	Vascular dementia of acute onset
**Eu011**	**Xa0IH**	Multi-infarct dementia	**F01.1**	Multi-infarct dementia
**Eu012**	**X003T**	Subcortical vascular dementia	**F01.2**	Subcortical vascular dementia
**Eu01y**	**Eu01y**	Other vascular dementia	**F01.8**	Other vascular dementia
**Eu01z**	**Eu01z**	Vascular dementia, unspecified	**F01.9**	Vascular dementia, unspecified
			**F02**	Dementia in other diseases classified elsewhere
**Eu020**	**X0034**	Picks Disease (Read)/Frontotemporal dementia includes Picks Disease and progressive isolated aphasia(CTv3)	**F02.0**	Dementia in Pick's disease
			**F02.1**	Dementia in Creutzfeldt-Jakob disease
			**F02.2**	Dementia in Huntington's disease
**Eu023**	**Eu023**	Dementia in Parkinson's disease	**F02.3**	Dementia in Parkinson's disease
			**F02.8**	Dementia in other specified diseases classified elsewhere

For peer review only - http://bmjopen.bmj.com/site/about/guidelines.xhtml

Table S1: Continued

Read codes	ctv3_codes	Read Code Description	ICD Code	ICD Description
**Eu02y**	**X0034**	Dementia in other diseases specified elsewhere (Read)/Frontotemporal dementia includes Pick's Disease and progressive isolated aphasia	**G31**	Other degenerative diseases of nervous system, not elsewhere classified
			**G31.0**	Frontotemporal dementia
**Eu025**	**X003A**	Lewy body dementia	**G31.8**	Other specified degenerative diseases of nervous system<br>Grey-matter degeneration [Alpers]<br>Lewy body(ies)(dementia)(disease)<br>Subacute necrotizing encephalopathy [Leigh]
	**XaKyY**	Lewy body dementia		
**Eu02z**	**XE1Z6**	Unspecified dementia	**F03**	Unspecified dementia
			**F05.1**	Delirium superimposed on dementia
	**X002w**	Dementia		
	**X00R2**	Senile dementia		
**1461**	**1461**	History of dementia		
**Eu107**<br>**Eu10711**<br>**E012**<br>**E0120**	**Xa25J**	Alcoholic dementia	**F10.7**	Residual and late-onset psychotic disorder: Includes Alcoholic dementia Not otherwise specified<br>Chronic alcoholic brain syndrome<br>Dementia and other milder forms of persisting impairment of cognitive functions

Anti-Dementia Medications

- Donezepil (Aricept®Aricept Evess®) Read Code: dy1
- Galantamine (Reminyl®, Reminyl® XL) Read Code: dy3…
- Rivastigimine (Exelon) Read Code: dy2…
- Memantine hydrochloride Read Code: dB1..

For peer review only - http://bmjopen.bmj.com/site/about/guidelines.xhtml

Table S2: Comparison of socio-demographic characteristics between sub-population (N=4668) and rest of EPIC-Norfolk cohort.

	Cohort		GP Sub-cohort		P-Value
	N=25 777*		N=4668		
Socio-demographic					
**Mean (SD)**					
**Age at Baseline**	59.9	(9.6)	56.2	(8.0)	<0.001
**Age at dementia diagnosis**	83.7	(6.6)	84.4	(6.6)	0.7
**Sex, % women (n)**	54.6	(14 063)	57.4	(2681)	<0.001
**Education, % (n)**					
No qualifications	39.8	(10 240)	33.1	(1546)	
O/ A level Standard	48.6	(12 510)	51.6	(2407)	<0.001
Graduate Level	11.6	(2992)	15.2	(711)	
**Social Class, % (n)**					
Professional	6.4	(1616)	8.5	(388)	
Managerial	35.2	(8833)	35.5	(1627)	
Skilled Non-Manual	16.7	(4199)	15.2	(697)	<0.001
Skilled Manual	23.7	(5948)	23.2	(1062)	
Semi-Skilled	14.1	(3545)	13.0	(597)	
Non-Skilled	3.8	(945)	4.6	(211)	
Dementia, % (N)	10.1	(2609)	4.4	(209)	<0.001

* Using full cohort (30,445) who consented, but did not attend a health examination as the sub population was based on this denominator and not 25,639 (attended the first health examination) at baseline

For peer review only - http://bmjopen.bmj.com/site/about/guidelines.xhtml

Table S3: Summary of responses from 8 of the collaborating Practices providing GP data.

Response from Questionnaire sent to GP Practice	Number
Used both Read Code and Medication names in searches	7
Included additional dementia codes not listed in the 'Clean up exercise' protocol	4
Information available in records as coded and free text data (free text not provided for this study)	6 (0 Practices used free text alone)
Practices experiencing difficulty with using dementia Read Codes	6=No 1=Yes<br>1-No response
Does the Practice carry out In-house cognitive testing (Yes)	7
Reason for patient cognitive assessment/referral to memory clinic	7=Patient concern<br>6= GP Judgement<br>6= Opportunistic testing
How is a confirmed dementia diagnosis made	8= Following secondary referral<br>4= including GP judgement
How is information of a dementia diagnosis received by the Practice	7= Free text (As a letter)<br>1= Free text plus ICD code
How is information of a dementia diagnosis entered onto the local GP database	7= Read Code<br>1= (Free text, ICD and Read code)
Were all cases identified from searches already included on QOF* register	3=Yes<br>5=No
Was there a discrepancy between QOF register and Read Code search	5=Yes<br>3=No

*QOF: Quality of Framework

For peer review only

The RECORD statement – checklist of items, extended from the STROBE statement, that should be reported in observational studies using routinely collected health data.

	Item No.	STROBE items	Location in manuscript where items are reported	RECORD items	Location in manuscript where items are reported
**Title and abstract**					
	1	(a) Indicate the study's design with a commonly used term in the title or the abstract (b) Provide in the abstract an informative and balanced summary of what was done and what was found		RECORD 1.1: The type of data used should be specified in the title or abstract. When possible, the name of the databases used should be included.<br><br>RECORD 1.2: If applicable, the geographic region and timeframe within which the study took place should be reported in the title or abstract.<br><br>RECORD 1.3: If linkage between databases was conducted for the study, this should be clearly stated in the title or abstract.	Title and abstract<br><br>Title and abstract<br><br>Title and abstract
**Introduction**					
Background rationale	2	Explain the scientific background and rationale for the investigation being reported			Pages 3-4
Objectives	3	State specific objectives, including any prespecified hypotheses			Page 4
**Methods**					
Study Design	4	Present key elements of study design early in the paper			Page 6
Setting	5	Describe the setting, locations, and relevant dates, including periods of recruitment, exposure, follow-up, and data collection			Pages 6-8

For peer review only

Participants	6	*(a) Cohort study* - Give the eligibility criteria, and the sources and methods of selection of participants. Describe methods of follow-up<br>*Case-control study* - Give the eligibility criteria, and the sources and methods of case ascertainment and control selection. Give the rationale for the choice of cases and controls<br>*Cross-sectional study* - Give the eligibility criteria, and the sources and methods of selection of participants<br><br>*(b) Cohort study* - For matched studies, give matching criteria and number of exposed and unexposed<br>*Case-control study* - For matched studies, give matching criteria and the number of controls per case		RECORD 6.1: The methods of study population selection (such as codes or algorithms used to identify subjects) should be listed in detail. If this is not possible, an explanation should be provided.<br><br>RECORD 6.2: Any validation studies of the codes or algorithms used to select the population should be referenced. If validation was conducted for this study and not published elsewhere, detailed methods and results should be provided.<br><br>RECORD 6.3: If the study involved linkage of databases, consider use of a flow diagram or other graphical display to demonstrate the data linkage process, including the number of individuals with linked data at each stage.	Page 6/Figure 1<br><br>Page 7<br><br>Figure 1
Variables	7	Clearly define all outcomes, exposures, predictors, potential confounders, and effect modifiers. Give diagnostic criteria, if applicable.		RECORD 7.1: A complete list of codes and algorithms used to classify exposures, outcomes, confounders, and effect modifiers should be provided. If these cannot be reported, an explanation should be provided.	Supplementary Table 1/Page 8
Data sources/ measurement	8	For each variable of interest, give sources of data and details of methods of assessment (measurement).<br>Describe comparability of assessment methods if there is more than one group			Pages 6-8

For peer review only

Bias	9	Describe any efforts to address potential sources of bias			Pages 17-18
Study size	10	Explain how the study size was arrived at			Page 7 Figure 1
Quantitative variables	11	Explain how quantitative variables were handled in the analyses. If applicable, describe which groupings were chosen, and why			Page 8
Statistical methods	12	(a) Describe all statistical methods, including those used to control for confounding<br>(b) Describe any methods used to examine subgroups and interactions<br>(c) Explain how missing data were addressed<br>(d) *Cohort study* - If applicable, explain how loss to follow-up was addressed<br>*Case-control study* - If applicable, explain how matching of cases and controls was addressed<br>*Cross-sectional study* - If applicable, describe analytical methods taking account of sampling strategy<br>(e) Describe any sensitivity analyses			Page 8<br><br><br><br>Page 7
Data access and cleaning methods		..		RECORD 12.1: Authors should describe the extent to which the investigators had access to the database population used to create the study population.	Pages 7-8

For peer review only

				RECORD 12.2: Authors should provide information on the data cleaning methods used in the study.	Pages 7-8
Linkage		..		RECORD 12.3: State whether the study included person-level, institutional-level, or other data linkage across two or more databases. The methods of linkage and methods of linkage quality evaluation should be provided.	Pages 7-8
**Results**					
Participants	13	(a) Report the numbers of individuals at each stage of the study (*e.g.*, numbers potentially eligible, examined for eligibility, confirmed eligible, included in the study, completing follow-up, and analysed)<br>(b) Give reasons for non-participation at each stage.<br>(c) Consider use of a flow diagram		RECORD 13.1: Describe in detail the selection of the persons included in the study (*i.e.,* study population selection) including filtering based on data quality, data availability and linkage. The selection of included persons can be described in the text and/or by means of the study flow diagram.	Figure 1<br>Pages 9-10<br>Supplementary Table 2
Descriptive data	14	(a) Give characteristics of study participants (*e.g.*, demographic, clinical, social) and information on exposures and potential confounders<br>(b) Indicate the number of participants with missing data for each variable of interest<br>(c) *Cohort study* - summarise follow-up time (*e.g.*, average and total amount)			Tables 1 and 2
Outcome data	15	*Cohort study* - Report numbers of outcome events or summary measures over time<br>*Case-control study* - Report numbers in each exposure			Pages 9-10

For peer review only

		category, or summary measures of exposure<br>*Cross-sectional study* - Report numbers of outcome events or summary measures			
Main results	16	(a) Give unadjusted estimates and, if applicable, confounder-adjusted estimates and their precision (e.g., 95% confidence interval). Make clear which confounders were adjusted for and why they were included<br>(b) Report category boundaries when continuous variables were categorized<br>(c) If relevant, consider translating estimates of relative risk into absolute risk for a meaningful time period			Table 3
Other analyses	17	Report other analyses done—e.g., analyses of subgroups and interactions, and sensitivity analyses			Pages 10-12
**Discussion**					
Key results	18	Summarise key results with reference to study objectives			Page 15
Limitations	19	Discuss limitations of the study, taking into account sources of potential bias or imprecision. Discuss both direction and magnitude of any potential bias		RECORD 19.1: Discuss the implications of using data that were not created or collected to answer the specific research question(s). Include discussion of misclassification bias, unmeasured confounding, missing data, and changing eligibility over time, as they pertain to the study being reported.	Page 15
Interpretation	20	Give a cautious overall interpretation of results considering objectives,			Pages 16-19

		limitations, multiplicity of analyses, results from similar studies, and other relevant evidence			
Generalisability	21	Discuss the generalisability (external validity) of the study results			Page 19
**Other Information**					
Funding	22	Give the source of funding and the role of the funders for the present study and, if applicable, for the original study on which the present article is based			Page 19
Accessibility of protocol, raw data, and programming code		..		RECORD 22.1: Authors should provide information on how to access any supplemental information such as the study protocol, raw data, or programming code.	Page 7

*Reference: Benchimol EI, Smeeth L, Guttmann A, Harron K, Moher D, Petersen I, Sørensen HT, von Elm E, Langan SM, the RECORD Working Committee. The REporting of studies Conducted using Observational Routinely-collected health Data (RECORD) Statement. *PLoS Medicine* 2015; in press.

*Checklist is protected under Creative Commons Attribution (CC BY) license.

For peer review only

BMJ Open

Evaluation of routinely collected records for dementia outcomes in United Kingdom (UK): a longitudinal study

Journal:	*BMJ Open*
Manuscript ID	bmjopen-2022-060931.R1
Article Type:	Original research
Date Submitted by the Author:	27-Apr-2022
Complete List of Authors:	Hayat, Shabina; University of Cambridge, Department of Psychiatry<br>Luben, Robert ; NIHR Moorfields Biomedical Research Centre; MRC Epidemiology Unit<br>Khaw, Kay-Tee; University of Cambridge; MRC Epidemiology Unit<br>Wareham, Nicholas; MRC Epidemiology Unit<br>Brayne, Carol; University of Cambridge, Department of Psychiatry
**Primary Subject Heading**:	Epidemiology
Secondary Subject Heading:	Public health
Keywords:	Dementia < NEUROLOGY, PUBLIC HEALTH, EPIDEMIOLOGY

SCHOLARONE™ Manuscripts

I, the Submitting Author has the right to grant and does grant on behalf of all authors of the Work (as defined in the below author licence), an exclusive licence and/or a non-exclusive licence for contributions from authors who are: i) UK Crown employees; ii) where BMJ has agreed a CC-BY licence shall apply, and/or iii) in accordance with the terms applicable for US Federal Government officers or employees acting as part of their official duties; on a worldwide, perpetual, irrevocable, royalty-free basis to BMJ Publishing Group Ltd ("BMJ") its licensees and where the relevant Journal is co-owned by BMJ to the co-owners of the Journal, to publish the Work in this journal and any other BMJ products and to exploit all rights, as set out in our licence.

The Submitting Author accepts and understands that any supply made under these terms is made by BMJ to the Submitting Author unless you are acting as an employee on behalf of your employer or a postgraduate student of an affiliated institution which is paying any applicable article publishing charge ("APC") for Open Access articles. Where the Submitting Author wishes to make the Work available on an Open Access basis (and intends to pay the relevant APC), the terms of reuse of such Open Access shall be governed by a Creative Commons licence – details of these licences and which Creative Commons licence will apply to this Work are set out in our licence referred to above.

Other than as permitted in any relevant BMJ Author's Self Archiving Policies, I confirm this Work has not been accepted for publication elsewhere, is not being considered for publication elsewhere and does not duplicate material already published. I confirm all authors consent to publication of this Work and authorise the granting of this licence.

For peer review only

Evaluation of routinely collected records for dementia outcomes in United Kingdom (UK):

a longitudinal study.

Authors: Shabina A Hayat,[1,2] Robert Luben, [3] Kay-Tee Khaw, [4] Nick Wareham,[44] Carol Brayne. [1]

1. Department of Psychiatry, Cambridge Public Health, University of Cambridge, Cambridge, UK
2. Department of Behavioural Science and Health, Institute of Epidemiology and Health Care, University College London, UK
3. NIHR Biomedical Research Centre at Moorfields Eye Hospital & UCL Institute of Ophthalmology, London, UK
4. MRC Epidemiology Unit, University of Cambridge, Cambridge, UK

Corresponding author:

Dr Shabina Hayat (Sah63@medschl.cam.ac.uk)
Department of Psychiatry, Cambridge Public Health
University of Cambridge,
Cambridge, UK

Current address:
Department of Behavioural Science and Health
Institute of Epidemiology and Health Care, University College London
Alternative e mail: Shabina.hayat@ucl.ac.uk

Word Count:

Abstract: 284 words

Main Text: 4026 words

For peer review only - http://bmjopen.bmj.com/site/about/guidelines.xhtml

Abstract

Objectives: To evaluate the characteristics of individuals recorded as having a dementia diagnosis in different routinely collected records and to examine the extent of overlap of dementia coding across data sources. Also, to present comparisons of secondary and primary care records providing value for researchers using routinely collected records for dementia outcome capture.

Study Design: A prospective cohort study

Setting and Participants: A cohort of 25,639 men and women in Norfolk, aged 40-79 years at recruitment (1993-1997) followed until 2018 linked to routinely collected to identify dementia cases. Data sources include mortality from death certification and National Health Service (NHS) hospital or secondary care records. Primary care records for a subset of the cohort were also reviewed.

Primary outcome measure: Diagnosis of Dementia (any-cause)

Results: Over two thousand participants (n= 2635 individuals) were found to have a dementia diagnosis recorded in one or more of the data sources examined. Limited concordance was observed across the secondary care data sources. We also observed discrepancies with primary care records for the sub-set and report on potential linkage-related selection bias

Conclusions: Use of different types of record linkage from varying parts of the UK's health system reveals differences in recorded dementia diagnosis, indicating that dementia can be identified to varying extents in different parts of the NHS system. However, there is considerable variation, and limited overlap in those identified. We present potential selection biases that might occur depending on whether cause of death, or primary and secondary care data sources are used. With the expansion of using routinely collected health data, researchers must be aware of these potential biases and inaccuracies, reporting carefully on the likely extent of limitations and challenges of the data sources they use.

Strengths and Limitations

- This cohort study explores the reliability of dementia diagnosis from different data sources
- This study has virtually complete follow-up of the cohort for over 25 years
- Exploring the complexity of dementia ascertainment in primary and secondary care settings
- An in-depth examination and new insight into the national mental healthcare dataset

For peer review only

Introduction

Routinely collected health records are powerful tools that have been increasingly used for research in the last decade. [1] Epidemiological studies depend on accurate, reliable, and relevant information from data sources. However, there is substantial heterogeneity in the methods for case-ascertainment in dementia literature, [2] with studies applying different criteria, with no 'gold standard'. [3] Accuracy of medical records, of whatever type, tends to be assumed in the literature. Compared with population based surveys, dementia is still known to be underreported in primary health care records, with individuals having manifest signs of dementia but no record of a dementia diagnosis, although this 'gap' is being closed more recently. [4] Dementia ascertainment is complex in primary and secondary care settings [5,6] as well on death certification. [7] There are many influences on whether dementia is likely to be recorded in these settings. Variations in dementia reporting have been noted across regions in UK [8,9].Data sources are prone to inconsistency, misclassification and are influenced by change in dementia practice and policy, [8] such as those summarised in Box 1 below.

In the UK, the National Health Service (NHS) holds data on all hospital admissions by condition, allowing dementia ascertainment from routine data sources. To date, mortality and Hospital Episode Statistics (HES) data have been the main sources of health records for epidemiological studies. [3,10,11] These data are coded using the International Classification of Diseases (ICD) which are mainly diagnostic codes. Using these sources alone seriously underestimates the number of dementia cases, as most dementia patients will not require hospitalisation for their dementia nor will they have dementia as a cause of death recorded on their death certificate. [10,12]

General practitioners (GP) maintain primary care records, which include information, not only for diagnoses, but also for administrative purposes, including details of specialist referrals such as to memory clinics. Diagnosis of dementia is usually initiated in a primary care setting, based on patient symptoms or caregivers' concerns. [13] GP records, in theory, should be a more complete source for dementia case ascertainment. The coding system used in primary care uses Read Codes, is separate from the ICD system used in secondary care and death registration. Codes for dementia, are numerous, complex and prone to coding error and misclassification. [5] Another data source for dementia, which seems to have had less use in research, is the national mental healthcare dataset (MHDS) which contain record-level data about individuals in contact with mental health services. The use of multiple systems across different settings and unfamiliar codes adds to the confusion of

For peer review only

dementia case ascertainment. [5] The impact of incomplete or inaccurate recording of dementia can affect analysis and interpretation of results. [14]. Furthermore, databases are influenced by the point data are collected in the patient care pathway [11] and may not be representative of the full population of people living with dementia, thus affecting generalisability of findings. [15]

The objective of this study was to describe the procedures for a better understanding of the characteristics of individuals recorded as having a dementia diagnosis in different health care records and examine the extent of overlap of dementia coding across data sources. We present novel insight into the MHDS and compare secondary and primary care records providing value for others relying on these types of data sources for outcome capture.

Ethics

EPIC-Norfolk has the permissions (Section 251 and where possible, explicit signed consent given by participants attending health examinations) to follow up through medical record linkage. Procedures for this study have been approved by NHS Research Ethics Committee (NRES REC Ref: 98CN01). The Regulatory approvals from the Confidentiality Advisory group (CAG) can be found at:

Section 251 Application number 059; https://www.hra.nhs.uk/planning-and-improving-research/application-summaries/confidentiality-advisory-group-registers/).

For the GP dementia case ascertainment and validation sub-study – we also obtained approval from Norfolk & Suffolk Primary & Community Care Research Office for access to GP Practices.

Patient and public involvement

The EPIC-Norfolk study actively promotes greater participant involvement in research. The EPIC-Norfolk Participant Advisory Panel (EPAP) is a consultative forum providing participant perspective on how research is prioritised, planned, conducted, communicated, and used. EPAP is viewed as a partnership between EPIC-Norfolk researchers and participants. Further details on EPAP can be found here:

https://www.epic-norfolk.org.uk/for-participants/epap/

For peer review only - http://bmjopen.bmj.com/site/about/guidelines.xhtml

Box 1: Changes in policy for public and practitioners which may have increased awareness of dementia and recording of cases in health records

2006: Introduction of dementia to Quality and Outcomes Framework (QOF) registers. QOF is an incentivising primary care scheme that rewards GP Practices for the provision of quality care and helps to standardise improvements in the delivery of primary medical services. [16] Participation in QOF is voluntary.

2009: The 2009 National Dementia Strategy was introduced to increase the public and professional awareness and understanding of dementia. Also, the dementia prevalence indicator to the Quality Outcomes Framework was introduced in primary care

2011: Changes to coding practice. For mortality coding, deaths previously coded with an underlying cause of unspecified cerebrovascular disease were reclassified as vascular dementia

2012: The Dementia Challenge was launched in March 2012 by Prime Minister, David Cameron. This programme superseded the national strategy from 2009- focus was on three main areas: bringing about improvements in health and care, creating dementia friendly communities and improving research

2013: The Dementia Commissioning for Quality and Innovation (CQUIN) was introduced (to encourage practitioners to identify those who potentially have dementia in a secondary care setting, most particularly applied to those over 75 years with unplanned hospital admissions

2014: Mortality coding revised - dementia coded as the underlying cause of death

2015: Prime Minister launched his Challenge on Dementia 2020, which set out to build on the achievements of the Prime Minister's Challenge on Dementia 2012-2015

For peer review only

Methods

Study Population and data collection

Participants were recruited to the European Prospective Investigation of Cancer (EPIC) in Norfolk. Details of this study have been previously described. [17] Briefly, 30,445 men and women (40–79 years) residents of Norfolk, England, were recruited to the study via their general practices. Of these 25,639 attended the baseline health examination between 1993 and 1997. Subsequent follow-ups have involved self-report of health and lifestyle postal questionnaires and further health examinations. [18] Virtually complete follow-up for cohort for mortality and hospital admissions in EPIC-Norfolk has been established via linkage to routinely collected National Health Service (NHS) databases in England (Hospital Episode Statistics, (HES) and for mortality data for all participants using their unique NHS number and date of birth.

For peer review only - http://bmjopen.bmj.com/site/about/guidelines.xhtml

Dementia ascertainment and diagnostic codes in hospital and death records and mental healthcare data

Data linkage to the EPIC-Norfolk cohort for NHS and mortality data was carried out by NHS Digital, a statutory body in England, permitted to receive identifiable patient data for linkage. The linked hospital records contain coded diagnostic information for all inpatient and day-case admissions. [19] We also obtained national mental healthcare data (MHDS), which contain record-level data about individuals in contact with mental health services including memory clinics.

Participants with incident dementia were defined as those free of dementia at the time of enrolment to the study and then identified with a dementia diagnosis recorded in medical records subsequently. For a subset, we compared the NHS Digital datasets of secondary care and mortality to GP records. GP records for individuals with a dementia diagnosis in secondary care but missing from data provided by practices were further reviewed by researchers in the third and final confirmation phase for any indication of a dementia code or mention in free text. Further details of the protocol and dementia codes provided in the Supplementary Information and Supplementary Table S1. QOF for dementia coding in primary care financially incentivises each recorded dementia diagnosis. Figure 1 is a diagrammatic representation of selection of participants and record linkage in the cohort.

Statistical Analyses

Definite dementia cases were defined as having one or more of the ICD-10, or (for GP records) Read code, for dementia. Age and sex-specific incidence rates for all-cause dementia and mortality rates were calculated for the entire cohort. The number and proportion of participants with a diagnosis of dementia from the three NHS Digital data sources separately and combined were examined. We also examined differences in socio-demographic characteristics (age at recruitment, sex, education, and social class) of dementia cases across the three NHS Digital datasets. Details on co-variates are provided in the Supplementary Information. We used Cox proportional-hazards models to compare the association of socio-demographic factors and risk of a dementia diagnosis across data sources, with mutual adjustment of co-variates in the model. Statistical analyses were performed using SPSS version 25.0 (IBM Corp., Armonk, NY, USA).

Figure 1: Flow diagram of selection of EPIC-Norfolk participants for record linkage

Figure 1: HERE

For peer review only - http://bmjopen.bmj.com/site/about/guidelines.xhtml

Results

There were 2635 cases of dementia identified from the cohort of 25,639 individuals at the censor date of 31st December 2018 after 25.8 years of follow-up. The youngest age of entry to the study at baseline was just below 40 and the oldest age of the participant at the censor date was 101 years. Out of the 2298 individuals with data on age of diagnosis, the minimum age of diagnosis was 54 years and maximum was 99 years. Figure 2 shows the relationship between age and dementia diagnosis from mid to later life. Table 1 shows the sex and age-specific cumulative incidence of dementia and deaths in the cohort. Increasing age was associated with increasing rates of dementia and death. This table reflects the higher mortality in men which results in higher absolute numbers and of dementia cases in women.

Figure 2: Scatter plot of age of diagnosis by age at baseline in the EPIC-Norfolk cohort

Figure 2: HERE

For peer review only

Table 1: Age and sex specific proportions of dementia and death in EPIC-Norfolk from 1996 until 31st December 2018 using all 3 secondary care data sources provided by NHS Digital for each age group by gender at baseline.

Age Band at baseline	Median age	Freq (N)	% with dementia recorded at any time in follow-up (N)	% Died (N)	P-Value
**Men (N=11 607)**					
<=59 Years	51.6	5915	3.2 (190)	17.4 (1028)	
60-64 Years	62.5	1848	12.3 (228)	46.7 (863)	
65-69 Years	67.5	1890	15.6 (294)	72.0 (1361)	<0.001
70-74 Years	72.5	1579	17.3 (273)	89.9(1420)	
>75 years	75.7	375	14.9 (56)	95.7 (359)	
**Women (N=14032)**					
<=59 Years	51.3	7656	2.9 (222)	11.9 (909)	
60-64 Years	62.6	2118	14.5 (308)	36.9 (782)	
65-69 Years	67.4	2103	22.6 (475)	55.4 (1166)	<0.001
70-74 Years	72.4	1766	27.0 (477)	80.4 (1419)	
>75 years	75.8	389	28.8 (112)	90.7 (353)	
**ALL (N=25 639)**					
<=59 Years	51.5	13571	3.0 (412)	14.3 (1937)	
60-64 Years	62.6	3966	13.5 (536)	41.5 (1645)	
65-69 Years	67.5	3993	19.3 (769)	63.3 (2527)	<0.001
70-74 Years	72.5	3345	22.8 (750)	84.7 (2839)	
>75 years	75.7	764	22.0 (168)	93.2 712)	

P values by Chi sq. for proportion of individuals with dementia in each age category. Results shown for men and women separately and combined (ALL)

On comparing characteristics across the different data sources, there were no major differences in terms of sex and socio-demographic profiles (Table 2). Those with dementia recorded from the mortality dataset were slightly older and in MHDS were younger. Most recorded diagnoses of dementia were found in HES, followed by mortality records, with the least number identified from the MHDS. The distribution of dementia diagnoses across the three NHS Digital data sources is shown in Figure 3. This figure shows very little overlap. Where there was concordance in cases for the MHDS and HES data sources (N=448), 71% (319) had a date of diagnosis in the MH dataset before the date of diagnosis in HES, (median (IQR) 0.7 years (0.3, 1.7 years) with 26% (116) with an earlier date in HES and (median (IQR) 0.6 years (0.2, 1.9 years). Only 3% (13) had the same date recorded in both.

For peer review only - http://bmjopen.bmj.com/site/about/guidelines.xhtml

Associations for socio -demographic factors and the presence of a recorded diagnosis of dementia, were similar across the three data sources (Table 3). Age was a stronger predictor in the mortality data and weakest in the mental health data. Having qualifications was associated with a lower risk of future recorded diagnosis of dementia, this was observed for mortality and HES data but not for the MHDS.

Figure 3: Distribution of cases (N=2635) across the three main data sources from NHS Digital followed up until 31st December 2018

Figure 3: HERE

For peer review only

Table 2: Comparison of characteristics of 'definite dementia' cases identified from the three data sources separately and combined.

	All 3 data sources HES/MHDS/Mortality		HES		MHDS		Mortality		P-value
	Definite dementia N=2635		Definite dementia N=2157		Definite dementia N=727		Definite dementia N=1276		
**Socio-demographic Mean (SD)**									
**Age at Baseline**	66.7	(6.5)	66.9	(6.3)	64.1	(6.5)	67.9	(5.9)	<0.001
**Age at Diagnosis**	83.8	(6.5)	83.7	(6.5)	82.7	(6.8)	84.6	(6.4)	<0.001
**Sex, % women (n)**	60.5	(1594)	59.8	(1290)	59.7	(434)	60.5	(772)	0.9
**Education, % (n)**									
No qualifications	48.4	(1274)	49.1	(1057)	45.3	(329)	50.4	(641)	
O/ A level Standard	42.6	(1120)	41.9	(903)	45.3	(329)	41.4	(527)	0.5
Graduate Level	9.0	(238)	9.0	(194)	9.4	(68)	8.2	(105)	
**Social Class, % (n)**									
Professional	6.3	(162)	6.3	(132)	5.6	(42)	6.0	(74)	
Managerial	33.9	(866)	33.6	(702)	32.0	(240)	34.0	(421)	
Skilled Non-Manual	20.5	(523)	20.5	(428)	21.1	(158)	21.7	(268)	
Skilled Manual	21.3	(544)	21.2	(442)	21.4	(160)	20.5	(253)	0.7
Semi-Skilled	14.2	(362)	14.1	(295)	17.2	(129)	13.6	(168)	
Non-Skilled	3.8	(98)	4.2	(88)	2.7	(20)	4.3	(53)	

Abbreviations: HES, Hospital Episode Statistics, MHDS, Mental Health Dataset, N, number; O/A, Ordinary or Advanced level; SD, Standard deviation

For peer review only - http://bmjopen.bmj.com/site/about/guidelines.xhtml

Table 3: Association of sociodemographic factors and dementia in the EPIC-Norfolk Cohort in the three data sources combined and individually, mutually adjusted for age, sex, education, and social class.

	All 3 data sources (Dementia=2555)			HES only (Dementia=2087)			MHDS only (Dementia=715)			Mortality only (Dementia=1229)		
Factor	HR	95% CI	P-Value	HR	95% CI	P-Value	HR	95% CI	P-Value	HR	95% CI	P-Value
Age per 5 years	2.19	(2.13, 2.26)	<0.001	2.23	(2.16, 2.31)	<0.001	1.80	(1.71, 1.89)	<0.001	2.57	(2.46, 2.69)	<0.001
Sex (Men)	0.98	(0.91, 1.06)	0.6	1.01	(0.92, 1.10)	0.9	1.03	(0.88, 1.20)	0.7	1.08	(0.96, 1.21)	0.2
Education (Any qualifications)	0.87	(0.80, 0.95)	0.002	0.87	(0.79, 0.95)	0.002	0.91	(0.78, 1.07)	0.2	0.84	(0.74, 0.94)	0.003
Social Class (Non-manual)	0.99	(0.91, 1.08)	0.8	0.98	(0.89, 1.07)	0.7	0.93	(0.79, 1.09)	0.4	1.03	(0.91, 1.16)	0.7

Abbreviations: CI, Confidence Interval; HES, Hospital Episode Statistics, HR, Hazard ratio; MHDS, Mental Health Dataset

For peer review only

The MHDS consisted of mainly administrative data such as mental health reviews, care programmes and pathways that include contacts with mental health care professionals (both in hospitals and in outpatient memory clinics and the community) as well as diagnostics and treatment codes. The service-level breakdown of the mental health data was not applicable here, as there was little additional diagnostic information. The latest release in 2017-2018 appeared to be the most complex, covering mental healthcare more comprehensively and containing diagnostic ICD10 codes that had been limited in the previous years.

For the sub-study involving validation with GP records, out of the 26 practices that were contacted, 14 agreed to participate, 6 declined and 6 did not respond. Of the 14 that agreed, 8 practices completed the questionnaire and 9 provided data. There were no criteria for selecting a practice for this validation, other than they were practices that were collaborating with the study. There was a mix of rural and city practices, classified as urban or town and fringe areas, [20] although the majority of the practices ultimately submitting data were city-based. Different types of staff carried out the linkage with their records, ranging from practice managers, IT managers, research nurses to a health care assistant.

In this sub-population, 4.4% (209 of the 4668 participants) were dementia cases identified from HES, mental health data, mortality and the GP records compared to 10.1% (2635) dementia cases out of 25,635 participants in the rest of the cohort. The individuals of the sub-study registered with the responding practices were younger, more likely to be women, with higher education and social class when compared to the rest of the cohort (Supplementary Table S2). Of the 209 respondents with a recorded diagnosis of dementia, 57 were found in both secondary and primary care data and 87 were in primary care records only. In this small study, almost all the practices were using Read codes and all reported their dementia cases to be confirmed through secondary referral to memory clinics. Five out of the eight practices reported that some dementia cases were not previously identified and so were missing on their QOF registers. The summarised responses to the questionnaire from GPs are given in Supplementary Information (Table S3). Participants who were prescribed one of the four dementia drugs (N=57), also had a Read code of a definite dementia diagnosis. There were no cases identified from drugs alone.

There were 65 participants who had a definite dementia diagnosis in the secondary care records, but not in their GP records. For most of these (51 cases), the reason for absence from GP systems was because the participant had died, and the dementia diagnoses had come from mortality records. For

For peer review only - http://bmjopen.bmj.com/site/about/guidelines.xhtml

the remaining (N=14), 3 participants had Mild Cognitive Impairment (MCI) or probable dementia and for 10 participants, we were unable to confirm diagnosis. This could be because they had died recently, or had moved to another practice, in which case their records would no longer be available to the GP. One patient had no indication of any dementia or dementia related condition in their record. Of the 57 cases that were in both the secondary and primary data sources, 30 cases were obtained from the initial GP data extract, with 27 missed by the practices. These cases were ascertained with further review of individual records by the researchers and would have been missed if the extra confirmation phase had not been a part of the study design. Distribution of dementia cases across primary and secondary care datasets are shown in Figure 4.

Figure 4: Distribution of cases (N=209) identified from primary and secondary care in sub-population of EPIC-Norfolk participants (N=4668).

Figure 4: HERE

For peer review only

For peer review only - http://bmjopen.bmj.com/site/about/guidelines.xhtml

Discussion

Medical records allow virtually complete follow-up for dementia research, however there are limitations in terms of accuracy, completeness and underestimation of cases across data sources. [3,6,11,21,22] This study confirms, that although there is some concordance for dementia across data sources, this is rather limited. Hospital records yielded the greatest number of cases, followed by death certification with the least number identified from the MHDS.

Limitations and strengths

As in other studies, [3,10,23] we interpreted the absence of a dementia diagnosis code as absence of the dementia; this is clearly not the case. Even though using medical records is less sensitive to an in-study algorithmic approach to dementia diagnosis, specificity in all these data sources is likely to be high, as a clinical diagnosis, particularly in primary care records is usually made after referral to a specialist. [24] Another weakness is that we did not inspect medical records for the entire cohort, and so likely to have missed cases, reducing sensitivity. Furthermore, we would have missed milder cases as we only included definite dementia diagnosis, other than when confirming cases, where we did search for milder impairment.

To maximise our outcome and minimising risk of misclassification, we did not analyse types of dementia separately. Misclassification is common, and the emphasis on clinically diagnosed Alzheimer's disease (AD) in the majority of individuals hides the fact that most people with dementia, namely those aged 80 and above have mixed pathologies in their brains. Those who die without dementia also have increasing expression of AD pathologies [25,26]. Treating dementia as a single entity at a population level is appropriate as many risk factors are shared. Misclassification may have some impact on associations for younger onset dementias, where the level of importance of this will depend on the purpose of the study. Using a broader classification also improves external validity. [27] Under-ascertainment is inevitable and should be considered when making prevalence estimations.

Another limitation is that we did not account for the competing risk of death, which will be high in this ageing population. However, the proportion of deaths by age in men and women were presented. Individuals reaching the end of life with other comorbidities may die before any diagnosis of dementia. [28] To account for the competing risk for death, death must be a discrete event from dementia, and given that we included dementia from mortality records, this overlap does not allow for competing risk of death to be estimated here. However, in the Cox regression models, these individuals would have been censored, and once censored, these individuals are no longer at risk.

For peer review only - http://bmjopen.bmj.com/site/about/guidelines.xhtml

Using simple socio-demographic factors to examine associations with future risk of a definite dementia diagnosis, we demonstrate small differences, across the main two data sources, HES and mortality, with more difference observed in the MHDS. The age differences observed across the three data sources is as anticipated, as younger individuals with memory concerns are more likely to be referred to memory clinics compared to older frailer individuals who are more likely to be identified through hospitals.

The lower risk of dementia diagnosis for those with qualifications observed for mortality and HES data but not for the MHDS, could be due the smaller numbers in the MHDS, or reflecting that there was no difference in terms of education as to who accesses mental health services. For the socio-demographic factors examined here, we had few missing data, however for more complex factors where there is likely to be more missing data, the potential for these differences to be greater. Such factors and unrecorded dementia cases in a data source could yield different study results. Studies have highlighted regional variation in rates of diagnosis and reliability of existing data. [3] We have demonstrated that this heterogeneity exists even in a single geographical region.

We were also able to draw on the relatively new MHDS from NHS Digital, as yet not widely used in research or described in detail. Where there was concordance in cases for the MHDS and HES data, we found that for most cases, the date of diagnosis was earlier in MHDS by just a few months. This is as expected, as the MHDS will be from referrals made from GP, where diagnosis of dementia is usually initiated by family or individuals themselves. [13]. All activity relating to patients receiving care for a suspected or diagnosed mental health, learning disability, or neurodevelopmental conditions is within scope of this dataset. This is a further source of clinical and operational data in the NHS that can be used for purposes other than direct patient care. Although the MHDS covers mental healthcare more comprehensively than the other sources, it is limited as most of the clinical information is not coded, but recorded in text, thus, not lending itself so easily large-scale analyses. [7]

We found MHDS to be complex, with information on individuals (from referral to final discharge) on contact with secondary mental health services. Each subsequent annual release of the mental health data was wider in scope than the previous version. The data in the final year of follow-up gave the greatest number of dementia cases by ICD code. The sharp increase in diagnosis codes shows how important understanding the policy and practice context is to assess the influences and potential biases inherent in use of routine data sources. Additional reasons include changes in coding practices in mental health services, or the way these data are extracted to include diagnoses as well as service

For peer review only - http://bmjopen.bmj.com/site/about/guidelines.xhtml

codes. The MHDS has far greater potential to provide more complete estimates of diagnosed dementia in the population in the UK. This dataset will need further reviewing for future work.

It is currently not possible to examine primary care records via databases such as the Clinical Practice Research Datalink (CPRD) for this cohort. As we had to rely on GP practices to extract the data, we were restricted, for practical reasons, to link to a subset only. The protocol was time and labour intensive. We did not observe the same level of concordance with hospital records as shown in other larger cohort studies. [10,23] Some of this discrepancy was due to patients having died as GP records for people who die are moved swiftly to NHS archives. Due to financial incentivisation, there has been an increase in dementia diagnosis in primary care, although concerns of overdiagnosis have been raised and should be monitored carefully. [4] Diagnosis of dementia is usually initiated in a primary care setting and could be considered to be the most complete single source of dementia case ascertainment, but underdiagnosis still exists [15,29,30] for various reasons including reluctance of GPs to diagnose dementia. [30]

As with the UK Biobank study, [23] we also found significant proportion of dementia cases in primary care records, that had not been found in hospital or mortality records. In EPIC-Norfolk, this figure was 42% (87 out of the 209 dementia cases identified) compared to 52% reported in UK Biobank. The lower proportion in our study reflects the older age of our cohort, and so more likely to appear in hospital and mortality records than the participants from UK Biobank. UK Biobank included participants from Scotland where data quality is high and centralisation of data, linkable health service datasets make data more accessible to research. This position is not currently shared by the other countries of UK, although may change in the near future with the establishment of Health Data Research UK (HDR-UK) the vision to improve population health, address health inequalities, and to drive efficient service provision. [31] Another large cohort study, the Million Women Study, found NHS hospital admission data to agree with primary care records, [10] This study only consisted of women, and also like UK Biobank, participants were younger.

The study in the sub-population of the EPIC-Norfolk cohort with GP records has highlighted several key issues. Even though previous work has shown that practices are confident in identifying dementia cases, [9] the complexity of 'missingness' is well recognised. [32] If we had relied on the initial data extract from practices, we would have missed almost half the dementia cases. These cases were only ascertained via the confirmation step which involved a more detailed interrogation of the medical records by the research team. This may be due to lack of technical capacity or capability and could be

For peer review only - http://bmjopen.bmj.com/site/about/guidelines.xhtml

a further reason for under-ascertainment. This reveals the potential scale of missed outcomes where known cases are not reflected in extraction of routine records, influencing studies using databases such as CPRD. [33] These findings highlight the importance of extending further training and knowledge of dementia coding in primary care.

It is clear from the characteristics and lower dementia incidence that the sub-population was different from the overall cohort with regards to age, education, and social class and therefore assuming generalisability to wider populations requires appropriate caveats. Although, there were no specific criteria set in approaching practices, the practices that responded were mainly city practices, and the patient-base in these practices was younger, more educated and of higher social class. It is likely that the more research-active practices responded. This bias is clearly reflected in the dementia rates, which was 10% in the EPIC-Norfolk cohort overall, compared to 4% in the sub-set from the nine GP practices that finally took part.

Whether secondary care data add any further to what can be found in GP record could not be explored in our small study. There were 10 additional participants with a record of dementia in cases from secondary care records, although we were unable to confirm these diagnoses as we could not access their records to check on the detailed information. One additional participant had no indication of dementia or dementia related condition in the GP records despite a HES record of dementia. This may be a more widespread issue and should be explored in future work involving a larger and wider range of practices.

Databases such as CPRD are extremely powerful data sources, but rely on how dementia is coded in primary care. [5] Furthermore, they are not representative of all practices in the UK based on geography and size. [32] Our findings reveal the potential scale of biases influencing studies using larger primary care databases. They also highlight the importance of extending further training and knowledge of dementia coding in primary care. HES and mortality also rely on data provided by practices. Our findings show the extent to which practice selection impact on dementia case ascertainment and thus on measured rates. Therefore, these data should be used and interpreted with caution.

Recently there have been changes to coding within primary care with the introduction of new codes SNOMED CT. [34] This new coding system will replace Read codes and eventually also be used in secondary care providing clarity and consistency. However, the implementation of this will take

For peer review only - http://bmjopen.bmj.com/site/about/guidelines.xhtml

several years and the impact of this is unknown. There is also potential in the mental health data that includes other service-related codes that could be utilised for further insight into the level of cognitive impairment in the community. There are several administrative codes that relate to low, moderate and severe cognitive impairment. This information could be used to supplement the diagnostic information and could be useful in ascertaining milder forms of impairment and dementia.

This study, provides confidence, that in UK, identification of cases via record linkage with hospital admission, mortality and primary care data is sufficient for epidemiologic analyses of risk factors of dementia. The reliability of these data for incidence and prevalence rates is more challenging due to variability in ascertainment and diagnostic criteria which may differ over time and in different populations. In summary, it is important to note that different data sources provide different information. Using a single data source would clearly underestimate dementia outcomes, and so drawing from multiple sources is the best approach to maximize dementia ascertainment from routinely collected health records [3,15] and to enhance generalisability. [15] Researchers must be fully aware of the strengths and limitations of the data sources they use, identifying the potential sources of bias [35] and be transparent in reporting on how these reflect on the accuracy of their findings.

Acknowledgments:

The authors would like to thank all study participants, general practitioners and the EPIC Norfolk study team for their contribution to this work. We would also like to thank Nichola Dalzell who played a huge part in the data collection phase of the study.

Funding

The EPIC-Norfolk study (DOI 10.22025/2019.10.105.00004) has received funding from the Medical Research Council (MR/N003284/1 MC-UU_12015/1 and MC_UU_00006/1), Cancer Research UK (C864/A14136) and NIHR https://www.nihr.ac.uk (Ref: NF-SI-0616-10090 to [CB]).

Data availability

Data are available under a Data Transfer Agreement to any bona fide researcher. Although the dataset is anonymized, the breadth of the data included and the multiplicity of variables in the analysis file as primary variables or confounding factors means that provision of the dataset to other researchers without a Data Transfer Agreement would constitute a risk. The contact for data request is: E-mail: EPIC-Norfolk@mrcepid.cam.ac.uk.

For peer review only - http://bmjopen.bmj.com/site/about/guidelines.xhtml

For peer review only

For peer review only - http://bmjopen.bmj.com/site/about/guidelines.xhtml

Contributorship Statement

SAH drafted the manuscript and undertook analysis of the data. SAH was the lead applicant for acquiring the NHS Digital data for health and mortality endpoints, including the mental health dataset, designed and conducted the case ascertainment, carried out the mapping of dementia codes and data cleaning.

RL created the dataset, carried out the data linkage and provided the graphics for Figure 3.

KTK, NW and CB are principal investigators who contributed to the conception and study design and guidance on the presentation and interpretation of these data. All authors contributed to data analysis and interpretation of the data.

Competing Interests: None

For peer review only

References

1 Casey JA, Schwartz BS, Stewart WF, *et al.* Using Electronic Health Records for Population Health Research: A Review of Methods and Applications. *Annu Rev Public Health* 2016;**37**:61–81. doi:10.1146/annurev-publhealth-032315-021353

2 Global Burden of Diseases (GBD) 2016 Dementia Collaborators. Global, regional, and national burden of Alzheimer's disease and other dementias, 1990-2016 a systematic analysis for the Global Burden of Disease Study 2016. *Lancet Neurol* 2019;**18**:88–106. doi:10.1016/S1474-4422(18)30403-4

3 Sibbett RA, Russ TC, Deary IJ, *et al.* Dementia ascertainment using existing data in UK longitudinal and cohort studies: a systematic review of methodology. *BMC Psychiatry* 2017;**17**:239. doi:10.1186/s12888-017-1401-4

4 Aldus CF, Arthur A, Dennington-Price A, *et al.* Undiagnosed dementia in primary care: a record linkage study. *Heal Serv Deliv Res* 2020;**8**:1–108. doi:10.3310/hsdr08200

5 Russell P, Banerjee S, Watt J, *et al.* Improving the identification of people with dementia in primary care: evaluation of the impact of primary care dementia coding guidance on identified prevalence. *BMJ Open* 2013;**3**:e004023. doi:10.1136/bmjopen-2013-004023

6 Sommerlad A, Perera G, Singh-Manoux A, *et al.* Accuracy of general hospital dementia diagnoses in England: Sensitivity, specificity, and predictors of diagnostic accuracy 2008–2016. *Alzheimer's Dement* 2018;**14**:933–43. doi:10.1016/j.jalz.2018.02.012

7 Perera G, Broadbent M, Callard F, *et al.* Cohort profile of the South London and Maudsley NHS Foundation Trust Biomedical Research Centre (SLaM BRC) Case Register: current status and recent enhancement of an Electronic Mental Health Record-derived data resource. *BMJ Open* 2016;**6**:e008721. doi:10.1136/bmjopen-2015-008721

8 Mukadam N, Livingston G, Rantell K, *et al.* Diagnostic rates and treatment of dementia before and after launch of a national dementia policy: An observational study using English national databases. *BMJ Open* 2014;**4**:1–8. doi:10.1136/bmjopen-2013-004119

9 Fox M, Fox C, Cruickshank W, *et al.* Understanding the dementia diagnosis gap in Norfolk and Suffolk: A survey of general practitioners. *Qual Prim Care* 2014;**22**:101–7.http://primarycare.imedpub.com/understanding-the-dementia-diagnosis-gap-in-norfolk-and-suffolk-a-survey-of-general-practitioners.pdf

10 Brown A, Kirichek O, Balkwill A, *et al.* Comparison of dementia recorded in routinely collected hospital admission data in England with dementia recorded in primary care. *Emerg Themes Epidemiol* 2016;**13**:1–9. doi:10.1186/s12982-016-0053-z

11 Wilkinson T, Ly A, Schnier C, *et al.* Identifying dementia cases with routinely collected health data: A systematic review. *Alzheimer's Dement* 2018;**14**:1038–51. doi:10.1016/J.JALZ.2018.02.016

12 Romero JP, Benito-León J, Louis ED, *et al.* Under reporting of dementia deaths on death certificates: A systematic review of population-based cohort studies. *J Alzheimer's Dis* 2014;**41**:213–21. doi:10.3233/JAD-132765

13 Brayne C, Fox C. Dementia Screening in Primary Care. *Public Health* 2007;**298**:2409–11.

14 Weuve J, Proust-Lima C, Power MC, *et al.* Guidelines for reporting methodological challenges and evaluating potential bias in dementia research. 2015;**11**:1098–109. doi:10.1016/j.jalz.2015.06.1885

For peer review only - http://bmjopen.bmj.com/site/about/guidelines.xhtml

15 Davis KAS, Mueller C, Ashworth M, *et al.* What gets recorded, counts: dementia recording in primary care compared with a specialist database. *Age Ageing* 2021;**50**:2206–13. doi:10.1093/ageing/afab164

16 NHS England » Update on Quality Outcomes Framework changes for 2021/22. https://www.england.nhs.uk/publication/update-on-quality-outcomes-framework-changes-for-2021-22/ (accessed 15 Oct 2021).

17 Day N, Oakes S, Luben R, *et al.* EPIC-Norfolk: study design and characteristics of the cohort. European Prospective Investigation of Cancer. *Br J Cancer* 1999;**80**:95–103.

18 Hayat SA, Luben R, Keevil VL, *et al.* Cohort profile: A prospective cohort study of objective physical and cognitive capability and visual health in an ageing population of men and women in Norfolk (EPIC-Norfolk 3). *Int J Epidemiol* 2014;**43**:1063–72. doi:10.1093/ije/dyt086

19 Luben R, Hayat S, Wareham N, *et al.* Predicting admissions and time spent in hospital over a decade in a population-based record linkage study: the EPIC-Norfolk cohort. *BMJ Open* 2016;**6**. doi:10.1136/bmjopen-2015-009461

20 British Household Panel Survey, waves 1-17 (1991-2008) User Documentation: Rural-Urban Indicator (URIND) Rural-Urban Indicator. 2008. http://doc.ukdataservice.ac.uk/doc/6032/mrdoc/pdf/6032userguide.pdf

21 Gao L, Calloway R, Zhao E, *et al.* Accuracy of death certification of dementia in population-based samples of older people: Analysis over time. *Age Ageing* 2018;**47**:589–94. doi:10.1093/ageing/afy068

22 Crowther GJE, Bennett MI, Holmes JD. How well are the diagnosis and symptoms of dementia recorded in older patients admitted to hospital? *Age Ageing* 2017;**46**:112–8. doi:10.1093/ageing/afw169

23 Wilkinson T, Schnier C, Bush K, *et al.* Identifying dementia outcomes in UK Biobank: a validation study of primary care, hospital admissions and mortality data. *Eur J Epidemiol* 2019;**34**:557–65. doi:10.1007/s10654-019-00499-1

24 Ford E, Greenslade N, Paudyal P, *et al.* Predicting dementia from primary care records: A systematic review and meta-analysis. Published Online First: 2018. doi:10.1371/journal.pone.0194735

25 Matthews FE, Brayne C, Lowe J, *et al.* Epidemiological pathology of dementia: Attributable-risks at death in the medical research council cognitive function and ageing study. *PLoS Med* 2009;**6**. doi:10.1371/journal.pmed.1000180

26 Bennett DA. Mixed pathologies and neural reserve: Implications of complexity for Alzheimer disease drug discovery. *PLoS Med* 2017;**14**. doi:10.1371/journal.pmed.1002256

27 Ritchie CW, Terrera GM, Quinn TJ. Dementia trials and dementia tribulations: methodological and analytical challenges in dementia research. *Alzheimers Res Ther* 2015;**7**:31. doi:10.1186/s13195-015-0113-6

28 Brayne C, Gao L, Dewey M, *et al.* Dementia before death in ageing societies - The promise of prevention and the reality. *PLoS Med* 2006;**3**:1922–30. doi:10.1371/journal.pmed.0030397

29 Connolly A, Gaehl E, Martin H, *et al.* Underdiagnosis of dementia in primary care: Variations in the observed prevalence and comparisons to the expected prevalence. *Aging Ment Health* 2011;**15**:978–84. doi:10.1080/13607863.2011.596805

30 Ford E, Sheppard J, Oliver S, *et al.* Automated detection of patients with dementia whose symptoms have been identified in primary care but have no formal diagnosis: A retrospective

For peer review only - http://bmjopen.bmj.com/site/about/guidelines.xhtml

case-control study using electronic primary care records. *BMJ Open* 2021;**11**. doi:10.1136/bmjopen-2020-039248

31 Ford E, Boyd A, Bowles JKF, *et al.* Our data, our society, our health: A vision for inclusive and transparent health data science in the United Kingdom and beyond. *Learn Heal Syst* 2019;**3**:e10191. doi:10.1002/lrh2.10191

32 Herrett E, Gallagher AM, Bhaskaran K, *et al.* Data Resource Profile: Clinical Practice Research Datalink (CPRD). *Int J Epidemiol* 2015;**44**:827–36. doi:10.1093/ije/dyv098

33 Donegan K, Fox N, Black N, *et al.* Trends in diagnosis and treatment for people with dementia in the UK from 2005 to 2015: a longitudinal retrospective cohort study. *Lancet Public Heal* 2017;**2**:e149–56. doi:10.1016/S2468-2667(17)30031-2

34 NHS Digital. SNOMED CT: A clinical vocabulary for electronic health records. https://digital.nhs.uk/services/terminology-and-classifications/snomed-ct

35 Bohensky MA, Jolley D, Sundararajan V, *et al.* Data Linkage: A powerful research tool with potential problems. *BMC Health Serv Res* 2010;**10**:1–7. doi:10.1186/1472-6963-10-346

For peer review only

For peer review only - http://bmjopen.bmj.com/site/about/guidelines.xhtml

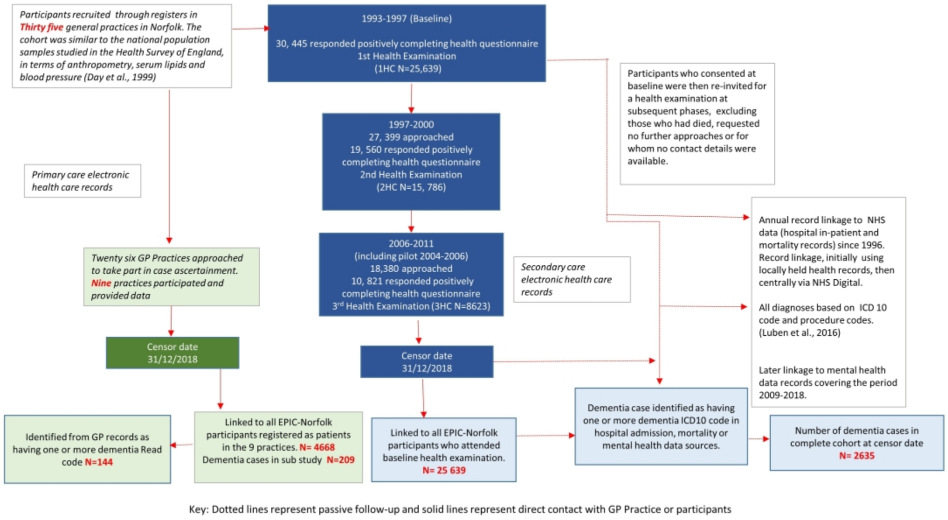

Figure 1: Flow diagram of selection of EPIC-Norfolk participants for record linkage

338x190mm (300 x 300 DPI)

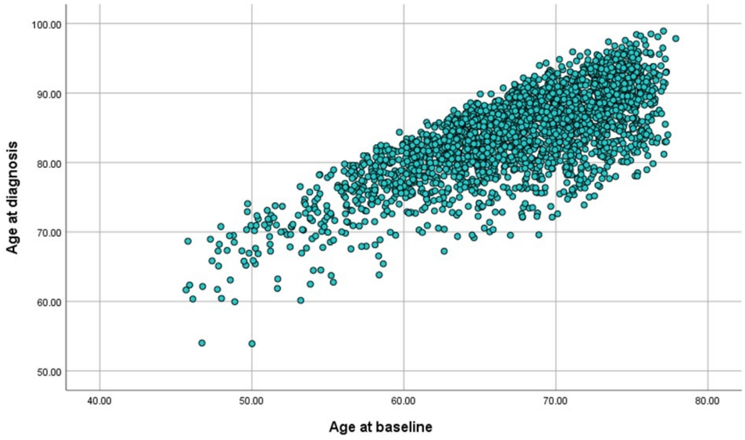

Scatter plot of age of diagnosis by age at baseline in the EPIC-Norfolk cohort

338x190mm (96 x 96 DPI)

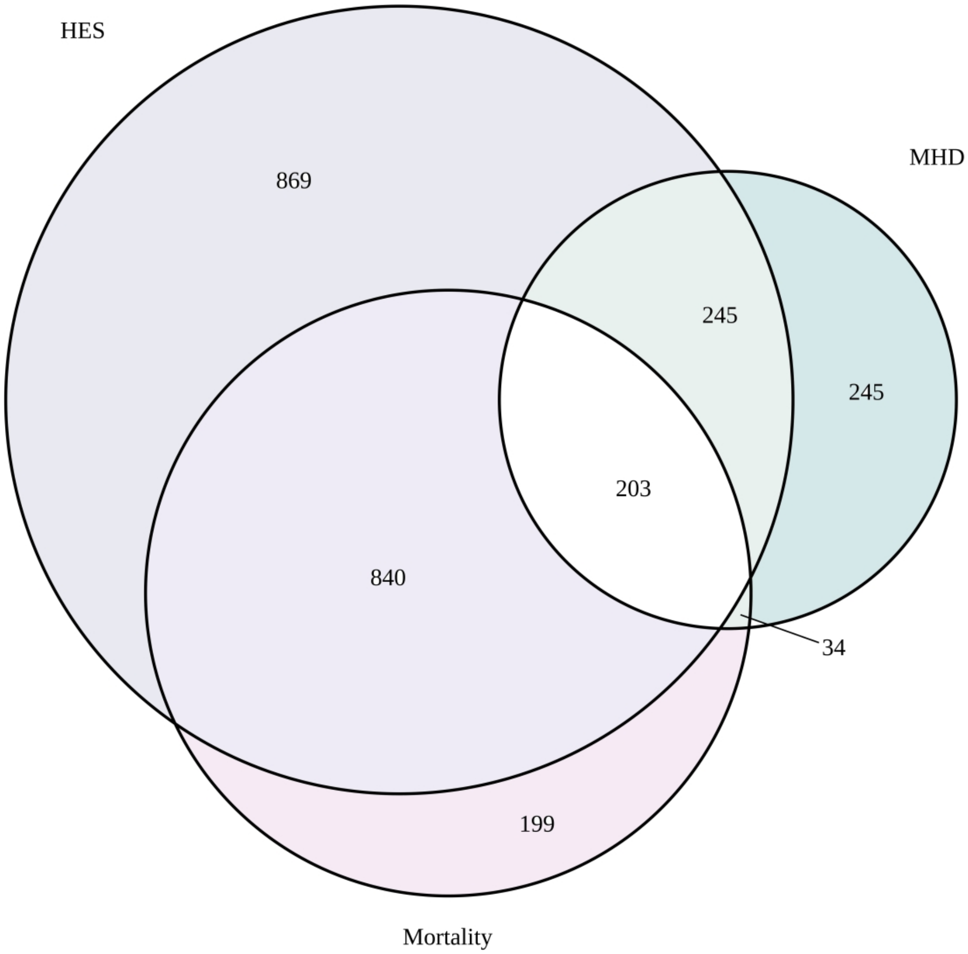

Distribution of cases (N=2635) across the three main data sources from NHS Digital followed up until 31st December 2018

987x987mm (144 x 144 DPI)

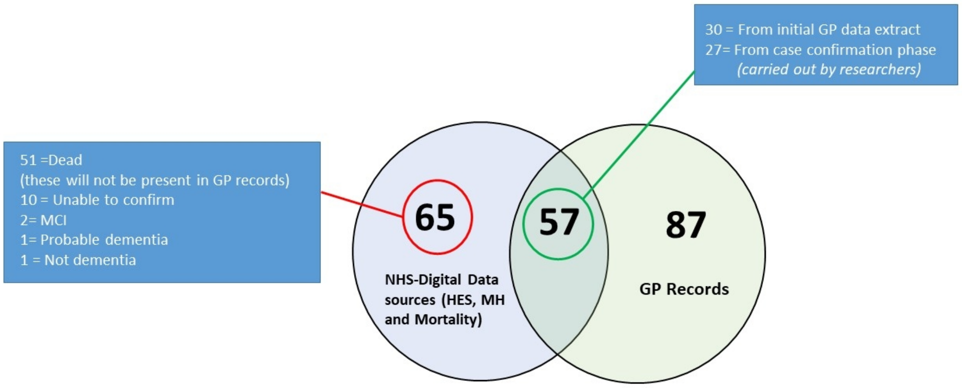

Figure 4: Distribution of cases (N=209) identified from primary and secondary care in sub-population of EPIC-Norfolk participants (N=4668).

338x190mm (96 x 96 DPI)

Supplementary Information

Title: Evaluation of routinely collected records for dementia outcomes in United Kingdom (UK): a longitudinal study.

Authors: Shabina A Hayat, Robert Luben, Kay-Tee Khaw, Nick Wareham, Carol Brayne.

Methods

Dementia ascertainment and diagnostic codes in hospital, death records and mental healthcare data

Individuals were followed from the date of consent, until the earliest date of a dementia diagnosis or date of death in or after 1996 when formal follow up by health records began in EPIC-Norfolk. To maximise the outcome data, we included any-cause dementia, the sub types of dementia were not analysed separately. Participants with no reports of death or dementia were censored on 31st December 2018. Dementia from HES records, death certificate or the MHDS was defined as any of the ICD-10 codes as listed in Supplementary Table S1.

Dementia case ascertainment through GP records

The protocol was based on a 'clean up' exercise [1] recommended by clinical commissioning groups (NHS bodies that commission services for defined populations) to improve dementia diagnosis in general practices. [2] We employed Read codes (Supplementary Table S1) identified as most commonly used for a dementia diagnoses. [1] Twenty-six collaborating GP practices were invited to participate. Those practices that agreed to take part in this exercise were provided with a detailed protocol and a list of patients on their register who were also EPIC-Norfolk participants. Each practice ran searches to generate lists of these respondents to establish which had a dementia diagnosis code, or were prescribed dementia defining medication, Donepezil, Galantamine, Rivastigmine or Memantine as identified by the relevant Read code (Supplementary Table S1).

Data were returned via a secure transfer system. Practices were also asked to complete a simple questionnaire. The full protocol and sample questionnaire are available on request. Practices were advised to take advantage of this exercise by comparing results of the search against their Quality and Outcomes Framework (QOF) [3] dementia register to check for discrepancies to improve their dementia diagnosis rates. [1] QOF for dementia coding in primary care financially incentivises each recorded dementia diagnosis.

For peer review only - http://bmjopen.bmj.com/site/about/guidelines.xhtml

In the second phase, cases identified from GP records were cross-checked with the NHS Digital data, and the level of agreement between the data sources was examined. GP records of individuals with a dementia diagnosis in secondary care but missing from the initial GP data extract were further reviewed by researchers in the third and final confirmation phase of this study. Individual records were reviewed by the researchers for any dementia read or ICD code or mention of dementia as free text. Initial case ascertainment from GPs was conducted between 2016-2017 and NHS Digital data were up to 31st March 2018, with case confirmation in GP records up to 20th December 2018, allowing few months after the follow-up period for the NHS Digital (secondary care) data.

Co-variates included in analyses

Education (the highest level attained) and social class were obtained from the baseline questionnaire. Education was categorised into three groups (i) No qualification (not completing school up to the age of 16), (ii) Completion of school up to the age of 16 or up to the age of 18 and finally (iii) those obtaining an education to graduate level (those who obtained a degree or equivalent) or above.

Social class was classified according to the Registrar General's occupation-based classification scheme into five main categories. [4] Social class I consists of professionals, class II includes managerial and technical occupations, class III is subdivided into non-manual and manual skilled workers (III non-manual and III manual), class IV consists of partly skilled workers, and class V comprises unskilled manual workers.

For peer review only

For peer review only - http://bmjopen.bmj.com/site/about/guidelines.xhtml

Table S1: Read and ICD 10 codes used in EPIC-Norfolk to search for 'Definite' dementia cases

Read codes	ctv3_codes	Read Code Description	ICD Code	ICD Description
**Eu00**	**Eu00**	Dementia in Alzheimer's disease	**F00**	Dementia in Alzheimer's disease
			**G30**	Alzheimer's disease
**Eu000**		Dementia in Alzheimer's disease with early onset	**F00.0**	Dementia in Alzheimer's disease with early onset
			**G30.0**	
**Eu001**	**X0030**	Dementia in Alzheimer's disease with late onset	**F00.1**	Dementia in Alzheimer disease with late onset
			**G30.1**	
	**XaIKC**	Alzheimer's disease with late onset		
**Eu002**	**Eu002**	Dementia in Alzheimer's disease, atypical or mixed type	**F00.2**	Dementia in Alzheimer disease, atypical or mixed type
			**G30.8**	Other Alzheimer's disease
**F110**		Dementia in Alzheimer's disease, unspecified	**F00.9**	Dementia in Alzheimer's disease, unspecified
			**G30.9**	
**Eu01**	**XE1Xs**	Vascular dementia	**F01**	Vascular dementia
			**F01.0**	Vascular dementia of acute onset
**Eu011**	**Xa0IH**	Multi-infarct dementia	**F01.1**	Multi-infarct dementia
**Eu012**	**X003T**	Subcortical vascular dementia	**F01.2**	Subcortical vascular dementia
**Eu01y**	**Eu01y**	Other vascular dementia	**F01.8**	Other vascular dementia
**Eu01z**	**Eu01z**	Vascular dementia, unspecified	**F01.9**	Vascular dementia, unspecified
			**F02**	Dementia in other diseases classified elsewhere
**Eu020/F111**	**X0034/F111**	Picks Disease (Read)/Frontotemporal dementia includes Picks Disease and progressive isolated aphasia(CTv3)	**F02.0**	Dementia in Pick's disease
			**F02.1**	Dementia in Creutzfeldt-Jakob disease
			**F02.2**	Dementia in Huntington's disease
**Eu023**	**Eu023**	Dementia in Parkinson's disease	**F02.3**	Dementia in Parkinson's disease
			**F02.8**	Dementia in other specified diseases classified elsewhere

For peer review only - http://bmjopen.bmj.com/site/about/guidelines.xhtml

Table S1: Continued

**Eu02y**	**X0034**	Dementia in other diseases specified elsewhere (Read)/Frontotemporal dementia includes Pick's Disease and progressive isolated aphasia	**G31**	Other degenerative diseases of nervous system, not elsewhere classified
			**G31.0**	Frontotemporal dementia
**Eu025 /F116**	**X003A**<br><br>**XaKyY**	Lewy body dementia<br><br>Lewy body dementia	**G31.8**	Other specified degenerative diseases of nervous system<br>Grey-matter degeneration [Alpers]<br>Lewy body(ies)(dementia)(disease)<br>Subacute necrotizing encephalopathy [Leigh]
**Eu02z**	**XE1Z6**	Unspecified dementia	**F03**	Unspecified dementia
			**F05.1**	Delirium superimposed on dementia
	**X002w**	Dementia		
	**X00R2**	Senile dementia		
**E00..**	**E00..**	Senile/presenile dementia		
**1461**	**1461**	History of dementia		
**Eu107**<br>**Eu10711**<br>**E012**<br>**E0120**	**Xa25J**	Alcoholic dementia	**F10.7**	Residual and late-onset psychotic disorder: Includes Alcoholic dementia NOS<br>Chronic alcoholic brain syndrome<br>Dementia and other milder forms of persisting impairment of cognitive functions

review only

Anti-Dementia Medications

- Donezepil (Aricept®Aricept Evess®) Read Code: dy1
- Galantamine (Reminyl®, Reminyl® XL) Read Code: dy3…
- Rivastigimine (Exelon) Read Code: dy2…
- Memantine hydrochloride Read Code: dB1..

For peer review only - http://bmjopen.bmj.com/site/about/guidelines.xhtml

Table S2: Comparison of socio-demographic characteristics between sub-population (N=4668) and rest of EPIC-Norfolk cohort.

	Cohort		GP Sub-cohort		P-Value
	N=25 777*		N=4668		
Socio-demographic					
**Mean (SD)**					
**Age at Baseline**	59.9	(9.6)	56.2	(8.0)	<0.001
**Age at dementia diagnosis**	83.7	(6.6)	84.4	(6.6)	0.7
**Sex, % women (n)**	54.6	(14 063)	57.4	(2681)	<0.001
**Education, % (n)**					
No qualifications	39.8	(10 240)	33.1	(1546)	
O/ A level Standard	48.6	(12 510)	51.6	(2407)	<0.001
Graduate Level	11.6	(2992)	15.2	(711)	
**Social Class, % (n)**					
Professional	6.4	(1616)	8.5	(388)	
Managerial	35.2	(8833)	35.5	(1627)	
Skilled Non-Manual	16.7	(4199)	15.2	(697)	<0.001
Skilled Manual	23.7	(5948)	23.2	(1062)	
Semi-Skilled	14.1	(3545)	13.0	(597)	
Non-Skilled	3.8	(945)	4.6	(211)	
Dementia, % (N)	10.1	(2609)	4.4	(209)	<0.001

* Using full cohort as the sub population was based on this denominator and not 25,639 from baseline

For peer review only

For peer review only - http://bmjopen.bmj.com/site/about/guidelines.xhtml

Table S3: Summary of responses from 8 of the collaborating Practices providing GP data.

Response from Questionnaire sent to GP Practice	Number
Used both Read Code and Medication names in searches	7
Included additional dementia codes not listed in the 'Clean up exercise' protocol	4
Information available in records as coded and free text data (free text not provided for this study)	6 (0 Practices used free text alone)
Practices experiencing difficulty with using dementia Read Codes	6=No 1=Yes<br>1-No response
Does the Practice carry out In-house cognitive testing (Yes)	7
Reason for patient cognitive assessment/referral to memory clinic	7=Patient concern<br>6= GP Judgement<br>6= Opportunistic testing
How is a confirmed dementia diagnosis made	8= Following secondary referral<br>4= including GP judgement
How is information of a dementia diagnosis received by the Practice	7= Free text (As a letter)<br>1= Free text plus ICD code
How is information of a dementia diagnosis entered onto the local GP database	7= Read Code<br>1= (Free text, ICD and Read code)
Were all cases identified from searches already included on QOF* register	3=Yes<br>5=No
Was there a discrepancy between QOF* register and Read Code search	5=Yes<br>3=No

* Quality and Outcomes Framework

Dementia from HES records, death certificate or the MHDS was defined as any of the ICD-10 codes as listed in Supplementary Table S1.

References

1 Russell P, Banerjee S, Watt J, *et al.* Improving the identification of people with dementia in primary care: evaluation of the impact of primary care dementia coding guidance on identified prevalence. *BMJ Open* 2013;**3**:e004023. doi:10.1136/bmjopen-2013-004023

2 London Clinical Networks. "Coding clean-up " Exercise Guidance to GPs to improve dementia coding , and raise diagnosis rates: A step by step approach. London, UK:

3 Mukadam N, Livingston G, Rantell K, *et al.* Diagnostic rates and treatment of dementia before and after launch of a national dementia policy: An observational study using English national databases. *BMJ Open* 2014;**4**:1–8. doi:10.1136/bmjopen-2013-004119

4 Shohaimi S, Welch A, Bingham S, *et al.* Residential area deprivation predicts fruit and vegetable consumption independently of individual educational level and occupational social class: a cross sectional population study in the Norfolk cohort of the European Prospective Investigation into Canc. *J Epidemiol Community Health* 2004;**58**:686–91. doi:10.1136/jech.2003.008490

For peer review only - http://bmjopen.bmj.com/site/about/guidelines.xhtml

The RECORD statement – checklist of items, extended from the STROBE statement, that should be reported in observational studies using routinely collected health data.

	Item No.	STROBE items	Location in manuscript where items are reported	RECORD items	Location in manuscript where items are reported
**Title and abstract**					
	1	(a) Indicate the study's design with a commonly used term in the title or the abstract (b) Provide in the abstract an informative and balanced summary of what was done and what was found		RECORD 1.1: The type of data used should be specified in the title or abstract. When possible, the name of the databases used should be included.<br><br>RECORD 1.2: If applicable, the geographic region and timeframe within which the study took place should be reported in the title or abstract.<br><br>RECORD 1.3: If linkage between databases was conducted for the study, this should be clearly stated in the title or abstract.	Title and abstract<br><br>Title and abstract<br><br>Title and abstract
**Introduction**					
Background rationale	2	Explain the scientific background and rationale for the investigation being reported			Pages 3-4
Objectives	3	State specific objectives, including any prespecified hypotheses			Page 4
**Methods**					
Study Design	4	Present key elements of study design early in the paper			Page 5-6 Supplementary Information Page 1
Setting	5	Describe the setting, locations, and relevant dates, including periods of recruitment, exposure, follow-up, and data collection			Pages 5-6 Supplementary Information Page 1

For peer review only

Participants	6	*(a) Cohort study* - Give the eligibility criteria, and the sources and methods of selection of participants. Describe methods of follow-up<br>*Case-control study* - Give the eligibility criteria, and the sources and methods of case ascertainment and control selection. Give the rationale for the choice of cases and controls<br>*Cross-sectional study* - Give the eligibility criteria, and the sources and methods of selection of participants<br><br>*(b) Cohort study* - For matched studies, give matching criteria and number of exposed and unexposed<br>*Case-control study* - For matched studies, give matching criteria and the number of controls per case		RECORD 6.1: The methods of study population selection (such as codes or algorithms used to identify subjects) should be listed in detail. If this is not possible, an explanation should be provided.<br><br>RECORD 6.2: Any validation studies of the codes or algorithms used to select the population should be referenced. If validation was conducted for this study and not published elsewhere, detailed methods and results should be provided.<br><br>RECORD 6.3: If the study involved linkage of databases, consider use of a flow diagram or other graphical display to demonstrate the data linkage process, including the number of individuals with linked data at each stage.	Page 5-6<br>`Figure 1<br><br>Page 6<br>Figure 1
Variables	7	Clearly define all outcomes, exposures, predictors, potential confounders, and effect modifiers. Give diagnostic criteria, if applicable.		RECORD 7.1: A complete list of codes and algorithms used to classify exposures, outcomes, confounders, and effect modifiers should be provided. If these cannot be reported, an explanation should be provided.	Supplementary Information Page 1 and Table S1/<br><br>Page 8
Data sources/ measurement	8	For each variable of interest, give sources of data and details of methods of assessment (measurement).<br>Describe comparability of assessment methods if there is more than one group			Supplementary Information Pages1-2<br><br>Pages 5-6

For peer review only

Bias	9	Describe any efforts to address potential sources of bias			Supplementary Information Page 2
Study size	10	Explain how the study size was arrived at			Page 5 Figure 1
Quantitative variables	11	Explain how quantitative variables were handled in the analyses. If applicable, describe which groupings were chosen, and why			Supplementary Information Page 2<br><br>Page 6
Statistical methods	12	(a) Describe all statistical methods, including those used to control for confounding<br>(b) Describe any methods used to examine subgroups and interactions<br>(c) Explain how missing data were addressed<br>(d) *Cohort study* - If applicable, explain how loss to follow-up was addressed<br>*Case-control study* - If applicable, explain how matching of cases and controls was addressed<br>*Cross-sectional study* - If applicable, describe analytical methods taking account of sampling strategy<br>(e) Describe any sensitivity analyses			Supplementary Information Page 2<br><br>Page 6<br><br>Supplementary Information Page 2<br><br>Page 6
Data access and cleaning methods		..		RECORD 12.1: Authors should describe the extent to which the investigators had access to the database population used to create the study population.	Supplementary Information Page 2<br><br>Page 6

For peer review only

				RECORD 12.2: Authors should provide information on the data cleaning methods used in the study.	
Linkage		..		RECORD 12.3: State whether the study included person-level, institutional-level, or other data linkage across two or more databases. The methods of linkage and methods of linkage quality evaluation should be provided.	Supplementary Information Page 2<br><br>Page 6
**Results**					
Participants	13	(a) Report the numbers of individuals at each stage of the study (*e.g.*, numbers potentially eligible, examined for eligibility, confirmed eligible, included in the study, completing follow-up, and analysed)<br>(b) Give reasons for non-participation at each stage.<br>(c) Consider use of a flow diagram		RECORD 13.1: Describe in detail the selection of the persons included in the study (*i.e.,* study population selection) including filtering based on data quality, data availability and linkage. The selection of included persons can be described in the text and/or by means of the study flow diagram.	Figure 1<br>Pages 7-8<br>Supplementary Table S2
Descriptive data	14	(a) Give characteristics of study participants (*e.g.*, demographic, clinical, social) and information on exposures and potential confounders<br>(b) Indicate the number of participants with missing data for each variable of interest<br>(c) *Cohort study* - summarise follow-up time (*e.g.*, average and total amount)			Tables 1 and 2 and Table S2
Outcome data	15	*Cohort study* - Report numbers of outcome events or summary measures over time<br>*Case-control study* - Report numbers in each exposure			Pages 7-12

For peer review only - http://bmjopen.bmj.com/site/about/guidelines.xhtml

		category, or summary measures of exposure<br>*Cross-sectional study* - Report numbers of outcome events or summary measures			
Main results	16	(a) Give unadjusted estimates and, if applicable, confounder-adjusted estimates and their precision (e.g., 95% confidence interval). Make clear which confounders were adjusted for and why they were included<br>(b) Report category boundaries when continuous variables were categorized<br>(c) If relevant, consider translating estimates of relative risk into absolute risk for a meaningful time period			Table 3<br>Figures 3 and 4
Other analyses	17	Report other analyses done—e.g., analyses of subgroups and interactions, and sensitivity analyses			Pages 11-12
**Discussion**					
Key results	18	Summarise key results with reference to study objectives			Page 13
Limitations	19	Discuss limitations of the study, taking into account sources of potential bias or imprecision. Discuss both direction and magnitude of any potential bias		RECORD 19.1: Discuss the implications of using data that were not created or collected to answer the specific research question(s). Include discussion of misclassification bias, unmeasured confounding, missing data, and changing eligibility over time, as they pertain to the study being reported.	Page 13
Interpretation	20	Give a cautious overall interpretation of results considering objectives,			Pages 13-17

For peer review only - http://bmjopen.bmj.com/site/about/guidelines.xhtml

		limitations, multiplicity of analyses, results from similar studies, and other relevant evidence			
Generalisability	21	Discuss the generalisability (external validity) of the study results			Page 16
**Other Information**					
Funding	22	Give the source of funding and the role of the funders for the present study and, if applicable, for the original study on which the present article is based			Page 17
Accessibility of protocol, raw data, and programming code		..		RECORD 22.1: Authors should provide information on how to access any supplemental information such as the study protocol, raw data, or programming code.	Page 17

*Reference: Benchimol EI, Smeeth L, Guttmann A, Harron K, Moher D, Petersen I, Sørensen HT, von Elm E, Langan SM, the RECORD Working Committee. The REporting of studies Conducted using Observational Routinely-collected health Data (RECORD) Statement. *PLoS Medicine* 2015; in press.

*Checklist is protected under Creative Commons Attribution (CC BY) license.

For peer review only

BMJ Open

Evaluation of routinely collected records for dementia outcomes in United Kingdom: a prospective cohort study.

Journal:	*BMJ Open*
Manuscript ID	bmjopen-2022-060931.R2
Article Type:	Original research
Date Submitted by the Author:	26-May-2022
Complete List of Authors:	Hayat, Shabina; University of Cambridge, Department of Psychiatry<br>Luben, Robert ; NIHR Moorfields Biomedical Research Centre; MRC Epidemiology Unit<br>Khaw, Kay-Tee; University of Cambridge; MRC Epidemiology Unit<br>Wareham, Nicholas; MRC Epidemiology Unit<br>Brayne, Carol; University of Cambridge, Department of Psychiatry
**Primary Subject Heading**:	Epidemiology
Secondary Subject Heading:	Public health
Keywords:	Dementia < NEUROLOGY, PUBLIC HEALTH, EPIDEMIOLOGY

SCHOLARONE™
Manuscripts

BMJ

I, the Submitting Author has the right to grant and does grant on behalf of all authors of the Work (as defined in the below author licence), an exclusive licence and/or a non-exclusive licence for contributions from authors who are: i) UK Crown employees; ii) where BMJ has agreed a CC-BY licence shall apply, and/or iii) in accordance with the terms applicable for US Federal Government officers or employees acting as part of their official duties; on a worldwide, perpetual, irrevocable, royalty-free basis to BMJ Publishing Group Ltd ("BMJ") its licensees and where the relevant Journal is co-owned by BMJ to the co-owners of the Journal, to publish the Work in this journal and any other BMJ products and to exploit all rights, as set out in our licence.

The Submitting Author accepts and understands that any supply made under these terms is made by BMJ to the Submitting Author unless you are acting as an employee on behalf of your employer or a postgraduate student of an affiliated institution which is paying any applicable article publishing charge ("APC") for Open Access articles. Where the Submitting Author wishes to make the Work available on an Open Access basis (and intends to pay the relevant APC), the terms of reuse of such Open Access shall be governed by a Creative Commons licence – details of these licences and which Creative Commons licence will apply to this Work are set out in our licence referred to above.

Other than as permitted in any relevant BMJ Author's Self Archiving Policies, I confirm this Work has not been accepted for publication elsewhere, is not being considered for publication elsewhere and does not duplicate material already published. I confirm all authors consent to publication of this Work and authorise the granting of this licence.

For peer review only

Evaluation of routinely collected records for dementia outcomes in United Kingdom:

a prospective cohort study.

Authors: Shabina A Hayat,[1,2] Robert Luben, [3] Kay-Tee Khaw, [4] Nick Wareham,[44]Carol Brayne. [1]

1. Department of Psychiatry, Cambridge Public Health, University of Cambridge, Cambridge, UK
2. Department of Behavioural Science and Health, Institute of Epidemiology and Health Care, University College London, UK
3. NIHR Biomedical Research Centre at Moorfields Eye Hospital & UCL Institute of Ophthalmology, London, UK
4. MRC Epidemiology Unit, University of Cambridge, Cambridge, UK

Corresponding author:

Dr Shabina Hayat (Sah63@medschl.cam.ac.uk)
Department of Psychiatry, Cambridge Public Health
University of Cambridge,
Cambridge, UK

Current address:
Department of Behavioural Science and Health
Institute of Epidemiology and Health Care, University College London
Alternative e mail: Shabina.hayat@ucl.ac.uk

Word Count:

Abstract: 284 words

Main Text: 4026 words

For peer review only

For peer review only - http://bmjopen.bmj.com/site/about/guidelines.xhtml

Abstract

Objectives: To evaluate the characteristics of individuals recorded as having a dementia diagnosis in different routinely collected records and to examine the extent of overlap of dementia coding across data sources. Also, to present comparisons of secondary and primary care records providing value for researchers using routinely collected records for dementia outcome capture.

Study Design: A prospective cohort study

Setting and Participants: A cohort of 25,639 men and women in Norfolk, aged 40-79 years at recruitment (1993-1997) followed until 2018 linked to routinely collected to identify dementia cases. Data sources include mortality from death certification and National Health Service (NHS) hospital or secondary care records. Primary care records for a subset of the cohort were also reviewed.

Primary outcome measure: Diagnosis of Dementia (any-cause)

Results: Over two thousand participants (n= 2635 individuals) were found to have a dementia diagnosis recorded in one or more of the data sources examined. Limited concordance was observed across the secondary care data sources. We also observed discrepancies with primary care records for the sub-set and report on potential linkage-related selection bias

Conclusions: Use of different types of record linkage from varying parts of the UK's health system reveals differences in recorded dementia diagnosis, indicating that dementia can be identified to varying extents in different parts of the NHS system. However, there is considerable variation, and limited overlap in those identified. We present potential selection biases that might occur depending on whether cause of death, or primary and secondary care data sources are used. With the expansion of using routinely collected health data, researchers must be aware of these potential biases and inaccuracies, reporting carefully on the likely extent of limitations and challenges of the data sources they use.

Strengths and Limitations

- This study has virtually complete follow-up of the cohort for over 25 years
- Exploring the complexity of dementia ascertainment in primary and secondary care settings
- An in-depth examination and new insight into the national mental healthcare dataset
- Using medical records, a less sensitive approach for dementia diagnosis

For peer review only - http://bmjopen.bmj.com/site/about/guidelines.xhtml

Introduction

Routinely collected health records are powerful tools that have been increasingly used for research in the last decade. [1] Epidemiological studies depend on accurate, reliable, and relevant information from data sources. However, there is substantial heterogeneity in the methods for case-ascertainment in dementia literature, [2] with studies applying different criteria, with no 'gold standard'. [3] Accuracy of medical records, of whatever type, tends to be assumed in the literature. Compared with population based surveys, dementia is still known to be underreported in primary health care records, with individuals having manifest signs of dementia but no record of a dementia diagnosis, although this 'gap' is being closed more recently. [4] Dementia ascertainment is complex in primary and secondary care settings [5,6] as well on death certification. [7] There are many influences on whether dementia is likely to be recorded in these settings. Variations in dementia reporting have been noted across regions in UK [8,9].Data sources are prone to inconsistency, misclassification and are influenced by change in dementia practice and policy, [8] such as those summarised in Box 1 below.

In the UK, the National Health Service (NHS) holds data on all hospital admissions by condition, allowing dementia ascertainment from routine data sources. To date, mortality and Hospital Episode Statistics (HES) data have been the main sources of health records for epidemiological studies. [3,10,11] These data are coded using the International Classification of Diseases (ICD) which are mainly diagnostic codes. Using these sources alone seriously underestimates the number of dementia cases, as most dementia patients will not require hospitalisation for their dementia nor will they have dementia as a cause of death recorded on their death certificate. [10,12]

General practitioners (GP) maintain primary care records, which include information, not only for diagnoses, but also for administrative purposes, including details of specialist referrals such as to memory clinics. Diagnosis of dementia is usually initiated in a primary care setting, based on patient symptoms or caregivers' concerns. [13] GP records, in theory, should be a more complete source for dementia case ascertainment. The coding system used in primary care uses Read Codes, is separate from the ICD system used in secondary care and death registration. Codes for dementia, are numerous, complex and prone to coding error and misclassification. [5] Another data source for dementia, which seems to have had less use in research, is the national mental healthcare dataset (MHDS) which contain record-level data about individuals in contact with mental health services. The use of multiple systems across different settings and unfamiliar codes adds to the confusion of

For peer review only - http://bmjopen.bmj.com/site/about/guidelines.xhtml

dementia case ascertainment. [5] The impact of incomplete or inaccurate recording of dementia can affect analysis and interpretation of results. [14]. Furthermore, databases are influenced by the point data are collected in the patient care pathway [11] and may not be representative of the full population of people living with dementia, thus affecting generalisability of findings. [15]

The objective of this study was to describe the procedures for a better understanding of the characteristics of individuals recorded as having a dementia diagnosis in different health care records and examine the extent of overlap of dementia coding across data sources. We present novel insight into the MHDS and compare secondary and primary care records providing value for others relying on these types of data sources for outcome capture.

For peer review only

For peer review only - http://bmjopen.bmj.com/site/about/guidelines.xhtml

Box 1: Changes in policy for public and practitioners which may have increased awareness of dementia and recording of cases in health records

2006: Introduction of dementia to Quality and Outcomes Framework (QOF) registers. QOF is an incentivising primary care scheme that rewards GP Practices for the provision of quality care and helps to standardise improvements in the delivery of primary medical services. [16] Participation in QOF is voluntary.

2009: The 2009 National Dementia Strategy was introduced to increase the public and professional awareness and understanding of dementia. Also, the dementia prevalence indicator to the Quality Outcomes Framework was introduced in primary care

2011: Changes to coding practice. For mortality coding, deaths previously coded with an underlying cause of unspecified cerebrovascular disease were reclassified as vascular dementia

2012: The Dementia Challenge was launched in March 2012 by Prime Minister, David Cameron. This programme superseded the national strategy from 2009- focus was on three main areas: bringing about improvements in health and care, creating dementia friendly communities and improving research

2013: The Dementia Commissioning for Quality and Innovation (CQUIN) was introduced (to encourage practitioners to identify those who potentially have dementia in a secondary care setting, most particularly applied to those over 75 years with unplanned hospital admissions

2014: Mortality coding revised - dementia coded as the underlying cause of death

2015: Prime Minister launched his Challenge on Dementia 2020, which set out to build on the achievements of the Prime Minister’s Challenge on Dementia 2012-2015

Methods

Study Population and data collection

Participants were recruited to the European Prospective Investigation of Cancer (EPIC) in Norfolk. Details of this study have been previously described. [17] Briefly, 30,445 men and women (40–79 years) residents of Norfolk, England, were recruited to the study via their general practices. Of these 25,639 attended the baseline health examination between 1993 and 1997. Subsequent follow-ups have involved self-report of health and lifestyle postal questionnaires and further health examinations. [18] Virtually complete follow-up for cohort for mortality and hospital admissions in EPIC-Norfolk has been established via linkage to routinely collected National Health Service (NHS) databases in England (Hospital Episode Statistics, (HES) and for mortality data for all participants using their unique NHS number and date of birth.

For peer review only - http://bmjopen.bmj.com/site/about/guidelines.xhtml

Dementia ascertainment and diagnostic codes in hospital and death records and mental healthcare data

Data linkage to the EPIC-Norfolk cohort for NHS and mortality data was carried out by NHS Digital, a statutory body in England, permitted to receive identifiable patient data for linkage. The linked hospital records contain coded diagnostic information for all inpatient and day-case admissions. [19] We also obtained national mental healthcare data (MHDS), which contain record-level data about individuals in contact with mental health services including memory clinics.

Participants with incident dementia were defined as those free of dementia at the time of enrolment to the study and then identified with a dementia diagnosis recorded in medical records subsequently. For a subset, we compared the NHS Digital datasets of secondary care and mortality to GP records. GP records for individuals with a dementia diagnosis in secondary care but missing from data provided by practices were further reviewed by researchers in the third and final confirmation phase for any indication of a dementia code or mention in free text. Further details of the protocol and dementia codes provided in the Supplementary Information and Supplementary Table S1. QOF for dementia coding in primary care financially incentivises each recorded dementia diagnosis. Figure 1 is a diagrammatic representation of selection of participants and record linkage in the cohort.

Figure 1: Flow diagram of selection of EPIC-Norfolk participants for record linkage

Figure 1: HERE

Ethics

EPIC-Norfolk has the permissions (Section 251 and where possible, explicit signed consent given by participants attending health examinations) to follow up through medical record linkage. Procedures for this study have been approved by NHS Research Ethics Committee (NRES REC Ref: 98CN01). The Regulatory approvals from the Confidentiality Advisory group (CAG) can be found at:

Section 251 Application number 059; https://www.hra.nhs.uk/planning-and-improving-research/application-summaries/confidentiality-advisory-group-registers/).

For the GP dementia case ascertainment and validation sub-study – we also obtained approval from Norfolk & Suffolk Primary & Community Care Research Office for access to GP Practices.

Patient and public involvement

The EPIC-Norfolk study actively promotes greater participant involvement in research. The EPIC-Norfolk Participant Advisory Panel (EPAP) is a consultative forum providing participant perspective on

For peer review only - http://bmjopen.bmj.com/site/about/guidelines.xhtml

how research is prioritised, planned, conducted, communicated, and used. EPAP is viewed as a partnership between EPIC-Norfolk researchers and participants. Further details on EPAP can be found here:
https://www.epic-norfolk.org.uk/for-participants/epap/

Statistical Analyses

Definite dementia cases were defined as having one or more of the ICD-10, or (for GP records) Read code, for dementia. Age and sex-specific incidence rates for all-cause dementia and mortality rates were calculated for the entire cohort. The number and proportion of participants with a diagnosis of dementia from the three NHS Digital data sources separately and combined were examined. We also examined differences in socio-demographic characteristics (age at recruitment, sex, education, and social class) of dementia cases across the three NHS Digital datasets. Details on co-variates are provided in the Supplementary Information. We used Cox proportional-hazards models to compare the association of socio-demographic factors and risk of a dementia diagnosis across data sources, with mutual adjustment of co-variates in the model. Statistical analyses were performed using SPSS version 25.0 (IBM Corp., Armonk, NY, USA).

Results

There were 2635 cases of dementia identified from the cohort of 25,639 individuals at the censor date of 31st December 2018 after 25.8 years of follow-up. The youngest age of entry to the study at baseline was just below 40 and the oldest age of the participant at the censor date was 101 years. Out of the 2298 individuals with data on age of diagnosis, the minimum age of diagnosis was 54 years and maximum was 99 years. Figure 2 shows the relationship between age and dementia diagnosis from mid to later life. Table 1 shows the sex and age-specific cumulative incidence of dementia and deaths in the cohort. Increasing age was associated with increasing rates of dementia and death. This table reflects the higher mortality in men which results in higher absolute numbers and of dementia cases in women.

Figure 2: Scatter plot of age of diagnosis by age at baseline in the EPIC-Norfolk cohort

Figure 2: HERE

For peer review only - http://bmjopen.bmj.com/site/about/guidelines.xhtml

Table 1: Age and sex specific proportions of dementia and death in EPIC-Norfolk from 1996 until 31st December 2018 using all 3 secondary care data sources provided by NHS Digital for each age group by gender at baseline.

Age Band at baseline	Median age	Freq (N)	% with dementia recorded at any time in follow-up (N)	% Died (N)	P-Value
**Men (N=11 607)**					
<=59 Years	51.6	5915	3.2 (190)	17.4 (1028)	
60-64 Years	62.5	1848	12.3 (228)	46.7 (863)	
65-69 Years	67.5	1890	15.6 (294)	72.0 (1361)	<0.001
70-74 Years	72.5	1579	17.3 (273)	89.9(1420)	
>75 years	75.7	375	14.9 (56)	95.7 (359)	
**Women (N=14032)**					
<=59 Years	51.3	7656	2.9 (222)	11.9 (909)	
60-64 Years	62.6	2118	14.5 (308)	36.9 (782)	
65-69 Years	67.4	2103	22.6 (475)	55.4 (1166)	<0.001
70-74 Years	72.4	1766	27.0 (477)	80.4 (1419)	
>75 years	75.8	389	28.8 (112)	90.7 (353)	
**ALL (N=25 639)**					
<=59 Years	51.5	13571	3.0 (412)	14.3 (1937)	
60-64 Years	62.6	3966	13.5 (536)	41.5 (1645)	
65-69 Years	67.5	3993	19.3 (769)	63.3 (2527)	<0.001
70-74 Years	72.5	3345	22.8 (750)	84.7 (2839)	
>75 years	75.7	764	22.0 (168)	93.2 712)	

P values by Chi sq. for proportion of individuals with dementia in each age category. Results shown for men and women separately and combined (ALL)

On comparing characteristics across the different data sources, there were no major differences in terms of sex and socio-demographic profiles (Table 2). Those with dementia recorded from the mortality dataset were slightly older and in MHDS were younger. Most recorded diagnoses of dementia were found in HES, followed by mortality records, with the least number identified from the MHDS. The distribution of dementia diagnoses across the three NHS Digital data sources is shown in Figure 3. This figure shows very little overlap. Where there was concordance in cases for the MHDS and HES data sources (N=448), 71% (319) had a date of diagnosis in the MH dataset before the date of diagnosis in HES, (median (IQR) 0.7 years (0.3, 1.7 years) with 26% (116) with an earlier date in HES and (median (IQR) 0.6 years (0.2, 1.9 years). Only 3% (13) had the same date recorded in both.

For peer review only - http://bmjopen.bmj.com/site/about/guidelines.xhtml

For peer review only

Associations for socio -demographic factors and the presence of a recorded diagnosis of dementia, were similar across the three data sources (Table 3). Age was a stronger predictor in the mortality data and weakest in the mental health data. Having qualifications was associated with a lower risk of future recorded diagnosis of dementia, this was observed for mortality and HES data but not for the MHDS.

Figure 3: Distribution of cases (N=2635) across the three main data sources from NHS Digital followed up until 31st December 2018

Figure 3: HERE

For peer review only

Table 2: Comparison of characteristics of 'definite dementia' cases identified from the three data sources separately and combined.

	All 3 data sources HES/MHDS/Mortality		HES		MHDS		Mortality		P-value
	Definite dementia N=2635		Definite dementia N=2157		Definite dementia N=727		Definite dementia N=1276		
**Socio-demographic Mean (SD)**									
**Age at Baseline**	66.7	(6.5)	66.9	(6.3)	64.1	(6.5)	67.9	(5.9)	<0.001
**Age at Diagnosis**	83.8	(6.5)	83.7	(6.5)	82.7	(6.8)	84.6	(6.4)	<0.001
**Sex, % women (n)**	60.5	(1594)	59.8	(1290)	59.7	(434)	60.5	(772)	0.9
**Education, % (n)**									
No qualifications	48.4	(1274)	49.1	(1057)	45.3	(329)	50.4	(641)	
O/ A level Standard	42.6	(1120)	41.9	(903)	45.3	(329)	41.4	(527)	0.5
Graduate Level	9.0	(238)	9.0	(194)	9.4	(68)	8.2	(105)	
**Social Class, % (n)**									
Professional	6.3	(162)	6.3	(132)	5.6	(42)	6.0	(74)	
Managerial	33.9	(866)	33.6	(702)	32.0	(240)	34.0	(421)	
Skilled Non-Manual	20.5	(523)	20.5	(428)	21.1	(158)	21.7	(268)	
Skilled Manual	21.3	(544)	21.2	(442)	21.4	(160)	20.5	(253)	0.7
Semi-Skilled	14.2	(362)	14.1	(295)	17.2	(129)	13.6	(168)	
Non-Skilled	3.8	(98)	4.2	(88)	2.7	(20)	4.3	(53)	

Abbreviations: HES, Hospital Episode Statistics, MHDS, Mental Health Dataset, N, number; O/A, Ordinary or Advanced level; SD, Standard deviation

For peer review only - http://bmjopen.bmj.com/site/about/guidelines.xhtml

Table 3: Association of sociodemographic factors and dementia in the EPIC-Norfolk Cohort in the three data sources combined and individually, mutually adjusted for age, sex, education, and social class.

	**All 3 data sources (Dementia=2555)**			**HES only (Dementia=2087)**			**MHDS only (Dementia=715)**			**Mortality only (Dementia=1229)**		
Factor	**HR**	**95% CI**	**P-Value**	**HR**	**95% CI**	**P-Value**	**HR**	**95% CI**	**P-Value**	**HR**	**95% CI**	**P-Value**
Age per 5 years	2.19	(2.13, 2.26)	<0.001	2.23	(2.16, 2.31)	<0.001	1.80	(1.71, 1.89)	<0.001	2.57	(2.46, 2.69)	<0.001
Sex (Men)	0.98	(0.91, 1.06)	0.6	1.01	(0.92, 1.10)	0.9	1.03	(0.88, 1.20)	0.7	1.08	(0.96, 1.21)	0.2
Education (Any qualifications)	0.87	(0.80, 0.95)	0.002	0.87	(0.79, 0.95)	0.002	0.91	(0.78, 1.07)	0.2	0.84	(0.74, 0.94)	0.003
Social Class (Non-manual)	0.99	(0.91, 1.08)	0.8	0.98	(0.89, 1.07)	0.7	0.93	(0.79, 1.09)	0.4	1.03	(0.91, 1.16)	0.7

Abbreviations: CI, Confidence Interval; HES, Hospital Episode Statistics, HR, Hazard ratio; MHDS, Mental Health Dataset

For peer review only

The MHDS consisted of mainly administrative data such as mental health reviews, care programmes and pathways that include contacts with mental health care professionals (both in hospitals and in outpatient memory clinics and the community) as well as diagnostics and treatment codes. The service-level breakdown of the mental health data was not applicable here, as there was little additional diagnostic information. The latest release in 2017-2018 appeared to be the most complex, covering mental healthcare more comprehensively and containing diagnostic ICD10 codes that had been limited in the previous years.

For the sub-study involving validation with GP records, out of the 26 practices that were contacted, 14 agreed to participate, 6 declined and 6 did not respond. Of the 14 that agreed, 8 practices completed the questionnaire and 9 provided data. There were no criteria for selecting a practice for this validation, other than they were practices that were collaborating with the study. There was a mix of rural and city practices, classified as urban or town and fringe areas, [20] although the majority of the practices ultimately submitting data were city-based. Different types of staff carried out the linkage with their records, ranging from practice managers, IT managers, research nurses to a health care assistant.

In this sub-population, 4.4% (209 of the 4668 participants) were dementia cases identified from HES, mental health data, mortality and the GP records compared to 10.1% (2635) dementia cases out of 25,635 participants in the rest of the cohort. The individuals of the sub-study registered with the responding practices were younger, more likely to be women, with higher education and social class when compared to the rest of the cohort (Supplementary Table S2). Of the 209 respondents with a recorded diagnosis of dementia, 57 were found in both secondary and primary care data and 87 were in primary care records only. In this small study, almost all the practices were using Read codes and all reported their dementia cases to be confirmed through secondary referral to memory clinics. Five out of the eight practices reported that some dementia cases were not previously identified and so were missing on their QOF registers. The summarised responses to the questionnaire from GPs are given in Supplementary Information (Table S3). Participants who were prescribed one of the four dementia drugs (N=57), also had a Read code of a definite dementia diagnosis. There were no cases identified from drugs alone.

There were 65 participants who had a definite dementia diagnosis in the secondary care records, but not in their GP records. For most of these (51 cases), the reason for absence from GP systems was because the participant had died, and the dementia diagnoses had come from mortality records. For

For peer review only - http://bmjopen.bmj.com/site/about/guidelines.xhtml

the remaining (N=14), 3 participants had Mild Cognitive Impairment (MCI) or probable dementia and for 10 participants, we were unable to confirm diagnosis. This could be because they had died recently, or had moved to another practice, in which case their records would no longer be available to the GP. One patient had no indication of any dementia or dementia related condition in their record. Of the 57 cases that were in both the secondary and primary data sources, 30 cases were obtained from the initial GP data extract, with 27 missed by the practices. These cases were ascertained with further review of individual records by the researchers and would have been missed if the extra confirmation phase had not been a part of the study design. Distribution of dementia cases across primary and secondary care datasets are shown in Figure 4.

Figure 4: Distribution of cases (N=209) identified from primary and secondary care in sub-population of EPIC-Norfolk participants (N=4668).

Figure 4: HERE

For peer review only

Discussion

Medical records allow virtually complete follow-up for dementia research, however there are limitations in terms of accuracy, completeness and underestimation of cases across data sources. [3,6,11,21,22] This study confirms that, although there is some concordance for dementia across data sources, this is rather limited. Hospital records yielded the greatest number of cases, followed by death certification with the least number identified from the MHDS.

Limitations and strengths

As in other studies, [3,10,23] we interpreted the absence of a dementia diagnosis code as absence of the dementia; this is clearly not the case. Even though using medical records is less sensitive to an in-study algorithmic approach to dementia diagnosis, specificity in all these data sources is likely to be high, as a clinical diagnosis, particularly in primary care records is usually made after referral to a specialist. [24] Another weakness is that we did not inspect medical records for the entire cohort, and so likely to have missed cases, reducing sensitivity. Furthermore, we would have missed milder cases as we only included definite dementia diagnosis, other than when confirming cases, where we did search for milder impairment.

To maximise our outcome and minimising risk of misclassification, we did not analyse types of dementia separately. Misclassification is common, and the emphasis on clinically diagnosed Alzheimer's disease (AD) in the majority of individuals hides the fact that most people with dementia, namely those aged 80 and above have mixed pathologies in their brains. Those who die without dementia also have increasing expression of AD pathologies [25,26]. Treating dementia as a single entity at a population level is appropriate as many risk factors are shared. Misclassification may have some impact on associations for younger onset dementias, where the level of importance of this will depend on the purpose of the study. Using a broader classification also improves external validity. [27] Under-ascertainment is inevitable and should be considered when making prevalence estimations.

Another limitation is that we did not account for the competing risk of death, which will be high in this ageing population. However, the proportion of deaths by age in men and women were presented. Individuals reaching the end of life with other comorbidities may die before any diagnosis of dementia. [28] To account for the competing risk for death, death must be a discrete event from dementia, and given that we included dementia from mortality records, this overlap does not allow for competing risk of death to be estimated here. However, in the Cox regression models, these individuals would have been censored, and once censored, these individuals are no longer at risk.

For peer review only - http://bmjopen.bmj.com/site/about/guidelines.xhtml

Using simple socio-demographic factors to examine associations with future risk of a definite dementia diagnosis, we demonstrate small differences, across the main two data sources, HES and mortality, with more difference observed in the MHDS. The age differences observed across the three data sources is as anticipated, as younger individuals with memory concerns are more likely to be referred to memory clinics compared to older frailer individuals who are more likely to be identified through hospitals.

The lower risk of dementia diagnosis for those with qualifications observed for mortality and HES data but not for the MHDS, could be due the smaller numbers in the MHDS, or reflecting that there was no difference in terms of education as to who accesses mental health services. For the socio-demographic factors examined here, we had few missing data, however for more complex factors where there is likely to be more missing data, the potential for these differences to be greater. Such factors and unrecorded dementia cases in a data source could yield different study results. Studies have highlighted regional variation in rates of diagnosis and reliability of existing data. [3] We have demonstrated that this heterogeneity exists even in a single geographical region.

We were also able to draw on the relatively new MHDS from NHS Digital, as yet not widely used in research or described in detail. Where there was concordance in cases for the MHDS and HES data, we found that for most cases, the date of diagnosis was earlier in MHDS by just a few months. This is as expected, as the MHDS will be from referrals made from GP, where diagnosis of dementia is usually initiated by family or individuals themselves. [13]. All activity relating to patients receiving care for a suspected or diagnosed mental health, learning disability, or neurodevelopmental conditions is within scope of this dataset. This is a further source of clinical and operational data in the NHS that can be used for purposes other than direct patient care. Although the MHDS covers mental healthcare more comprehensively than the other sources, it is limited as most of the clinical information is not coded, but recorded in text, thus, not lending itself so easily large-scale analyses. [7]

We found MHDS to be complex, with information on individuals (from referral to final discharge) on contact with secondary mental health services. Each subsequent annual release of the mental health data was wider in scope than the previous version. The data in the final year of follow-up gave the greatest number of dementia cases by ICD code. The sharp increase in diagnosis codes shows how important understanding the policy and practice context is to assess the influences and potential biases inherent in use of routine data sources. Additional reasons include changes in coding practices in mental health services, or the way these data are extracted to include diagnoses as well as service

For peer review only - http://bmjopen.bmj.com/site/about/guidelines.xhtml

codes. The MHDS has far greater potential to provide more complete estimates of diagnosed dementia in the population in the UK. This dataset will need further reviewing for future work.

It is currently not possible to examine primary care records via databases such as the Clinical Practice Research Datalink (CPRD) for this cohort. As we had to rely on GP practices to extract the data, we were restricted, for practical reasons, to link to a subset only. The protocol was time and labour intensive. We did not observe the same level of concordance with hospital records as shown in other larger cohort studies. [10,23] Some of this discrepancy was due to patients having died as GP records for people who die are moved swiftly to NHS archives. Due to financial incentivisation, there has been an increase in dementia diagnosis in primary care, although concerns of overdiagnosis have been raised and should be monitored carefully. [4] Diagnosis of dementia is usually initiated in a primary care setting and could be considered to be the most complete single source of dementia case ascertainment, but underdiagnosis still exists [15,29,30] for various reasons including reluctance of GPs to diagnose dementia. [30]

As with the UK Biobank study, [23] we also found significant proportion of dementia cases in primary care records, that had not been found in hospital or mortality records. In EPIC-Norfolk, this figure was 42% (87 out of the 209 dementia cases identified) compared to 52% reported in UK Biobank. The lower proportion in our study reflects the older age of our cohort, and so more likely to appear in hospital and mortality records than the participants from UK Biobank. UK Biobank included participants from Scotland where data quality is high and centralisation of data, linkable health service datasets make data more accessible to research. This position is not currently shared by the other countries of UK, although may change in the near future with the establishment of Health Data Research UK (HDR-UK) the vision to improve population health, address health inequalities, and to drive efficient service provision. [31] Another large cohort study, the Million Women Study, found NHS hospital admission data to agree with primary care records, [10] This study only consisted of women, and also like UK Biobank, participants were younger.

The study in the sub-population of the EPIC-Norfolk cohort with GP records has highlighted several key issues. Even though previous work has shown that practices are confident in identifying dementia cases, [9] the complexity of 'missingness' is well recognised. [32] If we had relied on the initial data extract from practices, we would have missed almost half the dementia cases. These cases were only ascertained via the confirmation step which involved a more detailed interrogation of the medical records by the research team. This may be due to lack of technical capacity or capability and could be

For peer review only - http://bmjopen.bmj.com/site/about/guidelines.xhtml

a further reason for under-ascertainment. This reveals the potential scale of missed outcomes where known cases are not reflected in extraction of routine records, influencing studies using databases such as CPRD. [33] These findings highlight the importance of extending further training and knowledge of dementia coding in primary care.

It is clear from the characteristics and lower dementia incidence that the sub-population was different from the overall cohort with regards to age, education, and social class and therefore assuming generalisability to wider populations requires appropriate caveats. Although, there were no specific criteria set in approaching practices, the practices that responded were mainly city practices, and the patient-base in these practices was younger, more educated and of higher social class. It is likely that the more research-active practices responded. This bias is clearly reflected in the dementia rates, which was 10% in the EPIC-Norfolk cohort overall, compared to 4% in the sub-set from the nine GP practices that finally took part.

Whether secondary care data add any further to what can be found in GP record could not be explored in our small study. There were 10 additional participants with a record of dementia in cases from secondary care records, although we were unable to confirm these diagnoses as we could not access their records to check on the detailed information. One additional participant had no indication of dementia or dementia related condition in the GP records despite a HES record of dementia. This may be a more widespread issue and should be explored in future work involving a larger and wider range of practices.

Databases such as CPRD are extremely powerful data sources, but rely on how dementia is coded in primary care. [5] Furthermore, they are not representative of all practices in the UK based on geography and size. [32] Our findings reveal the potential scale of biases influencing studies using larger primary care databases. They also highlight the importance of extending further training and knowledge of dementia coding in primary care. HES and mortality also rely on data provided by practices. Our findings show the extent to which practice selection impact on dementia case ascertainment and thus on measured rates. Therefore, these data should be used and interpreted with caution.

Recently there have been changes to coding within primary care with the introduction of new codes SNOMED CT. [34] This new coding system will replace Read codes and eventually also be used in secondary care providing clarity and consistency. However, the implementation of this will take

For peer review only - http://bmjopen.bmj.com/site/about/guidelines.xhtml

For peer review only

several years and the impact of this is unknown. There is also potential in the mental health data that includes other service-related codes that could be utilised for further insight into the level of cognitive impairment in the community. There are several administrative codes that relate to low, moderate and severe cognitive impairment. This information could be used to supplement the diagnostic information and could be useful in ascertaining milder forms of impairment and dementia.

This study provides confidence that identification of cases via record linkage with hospital admission, mortality and primary care data is sufficient for epidemiologic analyses of risk factors of dementia. The reliability of these data for incidence and prevalence rates is more challenging due to variability in ascertainment and diagnostic criteria which may differ over time and in different populations. In summary, it is important to note that different data sources provide different information. Using a single data source would clearly underestimate dementia outcomes, and so drawing from multiple sources is the best approach to maximize dementia ascertainment from routinely collected health records [3,15] and to enhance generalisability. [15] Researchers must be fully aware of the strengths and limitations of the data sources they use, identifying the potential sources of bias [35] and be transparent in reporting on how these reflect on the accuracy of their findings.

Acknowledgments:

The authors would like to thank all study participants, general practitioners and the EPIC Norfolk study team for their contribution to this work. We would also like to thank Nichola Dalzell who played a huge part in the data collection phase of the study.

Funding

The EPIC-Norfolk study (DOI 10.22025/2019.10.105.00004) has received funding from the Medical Research Council (MR/N003284/1 MC-UU_12015/1 and MC_UU_00006/1), Cancer Research UK (C864/A14136) and NIHR https://www.nihr.ac.uk (Ref: NF-SI-0616-10090 to [CB]).

Data availability

Data are available under a Data Transfer Agreement to any bona fide researcher. Although the dataset is anonymized, the breadth of the data included and the multiplicity of variables in the analysis file as primary variables or confounding factors means that provision of the dataset to other researchers without a Data Transfer Agreement would constitute a risk. The contact for data request is: E-mail: EPIC-Norfolk@mrcepid.cam.ac.uk.

For peer review only - http://bmjopen.bmj.com/site/about/guidelines.xhtml

For peer review only

Contributorship Statement

SAH drafted the manuscript and undertook analysis of the data. SAH was the lead applicant for acquiring the NHS Digital data for health and mortality endpoints, including the mental health dataset, designed and conducted the case ascertainment, carried out the mapping of dementia codes and data cleaning.

RL created the dataset, carried out the data linkage and provided the graphics for Figure 3.

KTK, NW and CB are principal investigators who contributed to the conception and study design and guidance on the presentation and interpretation of these data. All authors contributed to data analysis and interpretation of the data.

Competing Interests: None

For peer review only

For peer review only - http://bmjopen.bmj.com/site/about/guidelines.xhtml

References

1 Casey JA, Schwartz BS, Stewart WF, *et al.* Using Electronic Health Records for Population Health Research: A Review of Methods and Applications. *Annu Rev Public Health* 2016;**37**:61–81. doi:10.1146/annurev-publhealth-032315-021353

2 Global Burden of Diseases (GBD) 2016 Dementia Collaborators. Global, regional, and national burden of Alzheimer's disease and other dementias, 1990-2016 a systematic analysis for the Global Burden of Disease Study 2016. *Lancet Neurol* 2019;**18**:88–106. doi:10.1016/S1474-4422(18)30403-4

3 Sibbett RA, Russ TC, Deary IJ, *et al.* Dementia ascertainment using existing data in UK longitudinal and cohort studies: a systematic review of methodology. *BMC Psychiatry* 2017;**17**:239. doi:10.1186/s12888-017-1401-4

4 Aldus CF, Arthur A, Dennington-Price A, *et al.* Undiagnosed dementia in primary care: a record linkage study. *Heal Serv Deliv Res* 2020;**8**:1–108. doi:10.3310/hsdr08200

5 Russell P, Banerjee S, Watt J, *et al.* Improving the identification of people with dementia in primary care: evaluation of the impact of primary care dementia coding guidance on identified prevalence. *BMJ Open* 2013;**3**:e004023. doi:10.1136/bmjopen-2013-004023

6 Sommerlad A, Perera G, Singh-Manoux A, *et al.* Accuracy of general hospital dementia diagnoses in England: Sensitivity, specificity, and predictors of diagnostic accuracy 2008–2016. *Alzheimer's Dement* 2018;**14**:933–43. doi:10.1016/j.jalz.2018.02.012

7 Perera G, Broadbent M, Callard F, *et al.* Cohort profile of the South London and Maudsley NHS Foundation Trust Biomedical Research Centre (SLaM BRC) Case Register: current status and recent enhancement of an Electronic Mental Health Record-derived data resource. *BMJ Open* 2016;**6**:e008721. doi:10.1136/bmjopen-2015-008721

8 Mukadam N, Livingston G, Rantell K, *et al.* Diagnostic rates and treatment of dementia before and after launch of a national dementia policy: An observational study using English national databases. *BMJ Open* 2014;**4**:1–8. doi:10.1136/bmjopen-2013-004119

9 Fox M, Fox C, Cruickshank W, *et al.* Understanding the dementia diagnosis gap in Norfolk and Suffolk: A survey of general practitioners. *Qual Prim Care* 2014;**22**:101–7.http://primarycare.imedpub.com/understanding-the-dementia-diagnosis-gap-in-norfolk-and-suffolk-a-survey-of-general-practitioners.pdf

10 Brown A, Kirichek O, Balkwill A, *et al.* Comparison of dementia recorded in routinely collected hospital admission data in England with dementia recorded in primary care. *Emerg Themes Epidemiol* 2016;**13**:1–9. doi:10.1186/s12982-016-0053-z

11 Wilkinson T, Ly A, Schnier C, *et al.* Identifying dementia cases with routinely collected health data: A systematic review. *Alzheimer's Dement* 2018;**14**:1038–51. doi:10.1016/J.JALZ.2018.02.016

12 Romero JP, Benito-León J, Louis ED, *et al.* Under reporting of dementia deaths on death certificates: A systematic review of population-based cohort studies. *J Alzheimer's Dis* 2014;**41**:213–21. doi:10.3233/JAD-132765

13 Brayne C, Fox C. Dementia Screening in Primary Care. *Public Health* 2007;**298**:2409–11.

14 Weuve J, Proust-Lima C, Power MC, *et al.* Guidelines for reporting methodological challenges and evaluating potential bias in dementia research. 2015;**11**:1098–109. doi:10.1016/j.jalz.2015.06.1885

For peer review only - http://bmjopen.bmj.com/site/about/guidelines.xhtml

15 Davis KAS, Mueller C, Ashworth M, *et al.* What gets recorded, counts: dementia recording in primary care compared with a specialist database. *Age Ageing* 2021;**50**:2206–13. doi:10.1093/ageing/afab164

16 NHS England » Update on Quality Outcomes Framework changes for 2021/22. https://www.england.nhs.uk/publication/update-on-quality-outcomes-framework-changes-for-2021-22/ (accessed 15 Oct 2021).

17 Day N, Oakes S, Luben R, *et al.* EPIC-Norfolk: study design and characteristics of the cohort. European Prospective Investigation of Cancer. *Br J Cancer* 1999;**80**:95–103.

18 Hayat SA, Luben R, Keevil VL, *et al.* Cohort profile: A prospective cohort study of objective physical and cognitive capability and visual health in an ageing population of men and women in Norfolk (EPIC-Norfolk 3). *Int J Epidemiol* 2014;**43**:1063–72. doi:10.1093/ije/dyt086

19 Luben R, Hayat S, Wareham N, *et al.* Predicting admissions and time spent in hospital over a decade in a population-based record linkage study: the EPIC-Norfolk cohort. *BMJ Open* 2016;**6**. doi:10.1136/bmjopen-2015-009461

20 British Household Panel Survey, waves 1-17 (1991-2008) User Documentation: Rural-Urban Indicator (URIND) Rural-Urban Indicator. 2008. http://doc.ukdataservice.ac.uk/doc/6032/mrdoc/pdf/6032userguide.pdf

21 Gao L, Calloway R, Zhao E, *et al.* Accuracy of death certification of dementia in population-based samples of older people: Analysis over time. *Age Ageing* 2018;**47**:589–94. doi:10.1093/ageing/afy068

22 Crowther GJE, Bennett MI, Holmes JD. How well are the diagnosis and symptoms of dementia recorded in older patients admitted to hospital? *Age Ageing* 2017;**46**:112–8. doi:10.1093/ageing/afw169

23 Wilkinson T, Schnier C, Bush K, *et al.* Identifying dementia outcomes in UK Biobank: a validation study of primary care, hospital admissions and mortality data. *Eur J Epidemiol* 2019;**34**:557–65. doi:10.1007/s10654-019-00499-1

24 Ford E, Greenslade N, Paudyal P, *et al.* Predicting dementia from primary care records: A systematic review and meta-analysis. Published Online First: 2018. doi:10.1371/journal.pone.0194735

25 Matthews FE, Brayne C, Lowe J, *et al.* Epidemiological pathology of dementia: Attributable-risks at death in the medical research council cognitive function and ageing study. *PLoS Med* 2009;**6**. doi:10.1371/journal.pmed.1000180

26 Bennett DA. Mixed pathologies and neural reserve: Implications of complexity for Alzheimer disease drug discovery. *PLoS Med* 2017;**14**. doi:10.1371/journal.pmed.1002256

27 Ritchie CW, Terrera GM, Quinn TJ. Dementia trials and dementia tribulations: methodological and analytical challenges in dementia research. *Alzheimers Res Ther* 2015;**7**:31. doi:10.1186/s13195-015-0113-6

28 Brayne C, Gao L, Dewey M, *et al.* Dementia before death in ageing societies - The promise of prevention and the reality. *PLoS Med* 2006;**3**:1922–30. doi:10.1371/journal.pmed.0030397

29 Connolly A, Gaehl E, Martin H, *et al.* Underdiagnosis of dementia in primary care: Variations in the observed prevalence and comparisons to the expected prevalence. *Aging Ment Health* 2011;**15**:978–84. doi:10.1080/13607863.2011.596805

30 Ford E, Sheppard J, Oliver S, *et al.* Automated detection of patients with dementia whose symptoms have been identified in primary care but have no formal diagnosis: A retrospective

For peer review only - http://bmjopen.bmj.com/site/about/guidelines.xhtml

For peer review only

case-control study using electronic primary care records. *BMJ Open* 2021;**11**. doi:10.1136/bmjopen-2020-039248

31 Ford E, Boyd A, Bowles JKF, *et al.* Our data, our society, our health: A vision for inclusive and transparent health data science in the United Kingdom and beyond. *Learn Heal Syst* 2019;**3**:e10191. doi:10.1002/lrh2.10191

32 Herrett E, Gallagher AM, Bhaskaran K, *et al.* Data Resource Profile: Clinical Practice Research Datalink (CPRD). *Int J Epidemiol* 2015;**44**:827–36. doi:10.1093/ije/dyv098

33 Donegan K, Fox N, Black N, *et al.* Trends in diagnosis and treatment for people with dementia in the UK from 2005 to 2015: a longitudinal retrospective cohort study. *Lancet Public Heal* 2017;**2**:e149–56. doi:10.1016/S2468-2667(17)30031-2

34 NHS Digital. SNOMED CT: A clinical vocabulary for electronic health records. https://digital.nhs.uk/services/terminology-and-classifications/snomed-ct

35 Bohensky MA, Jolley D, Sundararajan V, *et al.* Data Linkage: A powerful research tool with potential problems. *BMC Health Serv Res* 2010;**10**:1–7. doi:10.1186/1472-6963-10-346

For peer review only

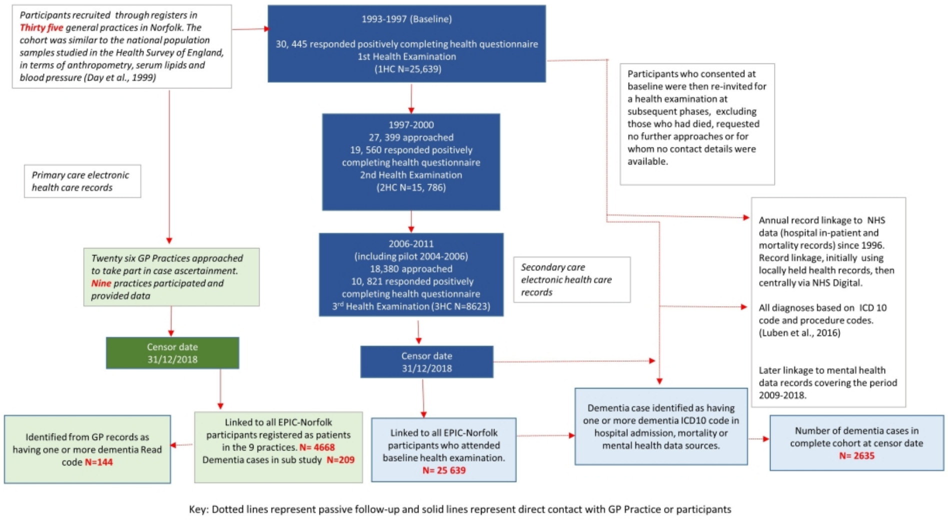

Figure 1: Flow diagram of selection of EPIC-Norfolk participants for record linkage

338x190mm (300 x 300 DPI)

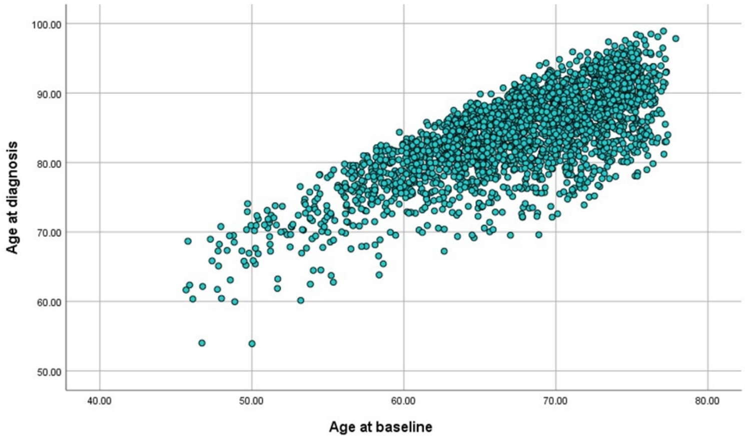

Scatter plot of age of diagnosis by age at baseline in the EPIC-Norfolk cohort

338x190mm (96 x 96 DPI)

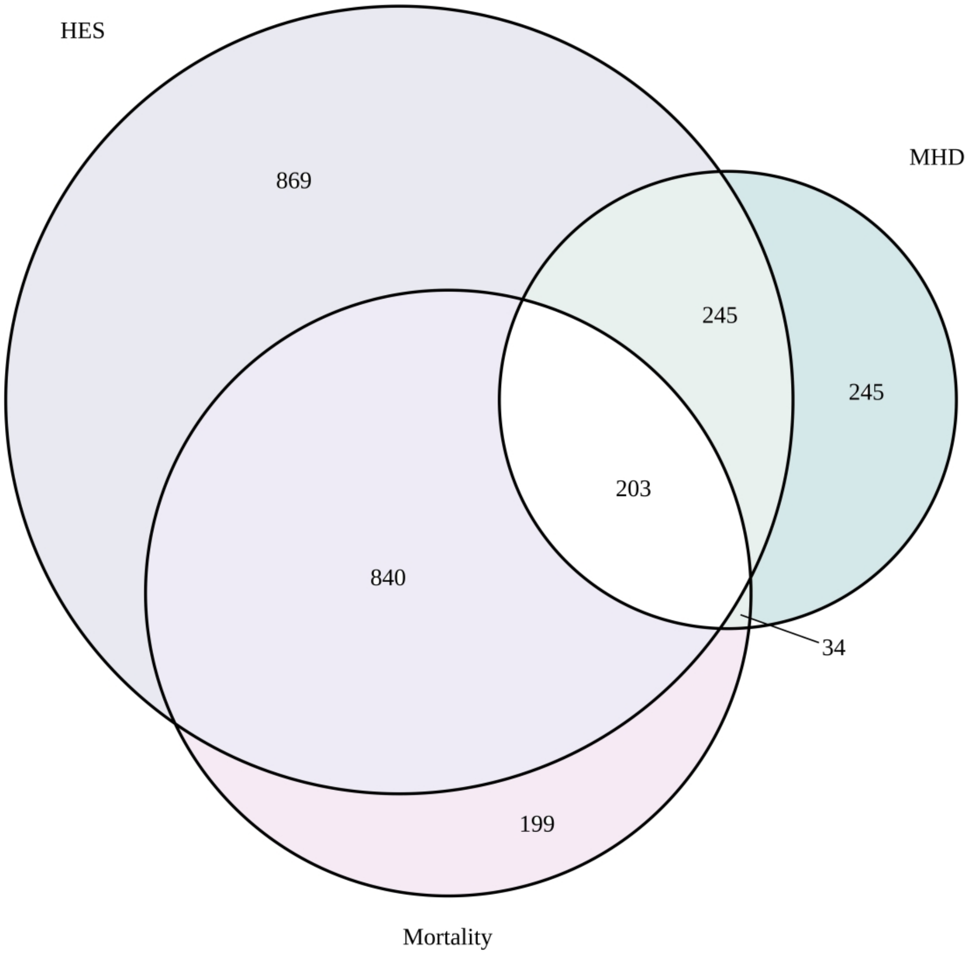

Distribution of cases (N=2635) across the three main data sources from NHS Digital followed up until 31st December 2018

987x987mm (144 x 144 DPI)

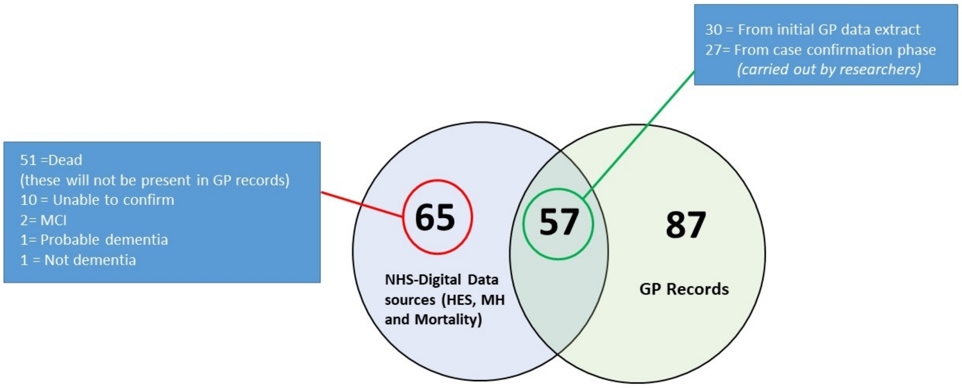

Figure 4: Distribution of cases (N=209) identified from primary and secondary care in sub-population of EPIC-Norfolk participants (N=4668).

338x190mm (96 x 96 DPI)

Supplementary Information

Title: Evaluation of routinely collected records for dementia outcomes in United Kingdom (UK): a longitudinal study.

Authors: Shabina A Hayat, Robert Luben, Kay-Tee Khaw, Nick Wareham, Carol Brayne.

Methods

Dementia ascertainment and diagnostic codes in hospital, death records and mental healthcare data

Individuals were followed from the date of consent, until the earliest date of a dementia diagnosis or date of death in or after 1996 when formal follow up by health records began in EPIC-Norfolk. To maximise the outcome data, we included any-cause dementia, the sub types of dementia were not analysed separately. Participants with no reports of death or dementia were censored on 31st December 2018. Dementia from HES records, death certificate or the MHDS was defined as any of the ICD-10 codes as listed in Supplementary Table S1.

Dementia case ascertainment through GP records

The protocol was based on a 'clean up' exercise [1] recommended by clinical commissioning groups (NHS bodies that commission services for defined populations) to improve dementia diagnosis in general practices. [2] We employed Read codes (Supplementary Table S1) identified as most commonly used for a dementia diagnoses. [1] Twenty-six collaborating GP practices were invited to participate. Those practices that agreed to take part in this exercise were provided with a detailed protocol and a list of patients on their register who were also EPIC-Norfolk participants. Each practice ran searches to generate lists of these respondents to establish which had a dementia diagnosis code, or were prescribed dementia defining medication, Donepezil, Galantamine, Rivastigmine or Memantine as identified by the relevant Read code (Supplementary Table S1).

Data were returned via a secure transfer system. Practices were also asked to complete a simple questionnaire. The full protocol and sample questionnaire are available on request. Practices were advised to take advantage of this exercise by comparing results of the search against their Quality and Outcomes Framework (QOF) [3] dementia register to check for discrepancies to improve their dementia diagnosis rates. [1] QOF for dementia coding in primary care financially incentivises each recorded dementia diagnosis.

For peer review only - http://bmjopen.bmj.com/site/about/guidelines.xhtml

In the second phase, cases identified from GP records were cross-checked with the NHS Digital data, and the level of agreement between the data sources was examined. GP records of individuals with a dementia diagnosis in secondary care but missing from the initial GP data extract were further reviewed by researchers in the third and final confirmation phase of this study. Individual records were reviewed by the researchers for any dementia read or ICD code or mention of dementia as free text. Initial case ascertainment from GPs was conducted between 2016-2017 and NHS Digital data were up to 31st March 2018, with case confirmation in GP records up to 20th December 2018, allowing few months after the follow-up period for the NHS Digital (secondary care) data.

Co-variates included in analyses

Education (the highest level attained) and social class were obtained from the baseline questionnaire. Education was categorised into three groups (i) No qualification (not completing school up to the age of 16), (ii) Completion of school up to the age of 16 or up to the age of 18 and finally (iii) those obtaining an education to graduate level (those who obtained a degree or equivalent) or above.

Social class was classified according to the Registrar General's occupation-based classification scheme into five main categories. [4] Social class I consists of professionals, class II includes managerial and technical occupations, class III is subdivided into non-manual and manual skilled workers (III non-manual and III manual), class IV consists of partly skilled workers, and class V comprises unskilled manual workers.

For peer review only

Table S1: Read and ICD 10 codes used in EPIC-Norfolk to search for 'Definite' dementia cases

Read codes	ctv3_codes	Read Code Description	ICD Code	ICD Description
**Eu00**	**Eu00**	Dementia in Alzheimer's disease	**F00**	Dementia in Alzheimer's disease
			**G30**	Alzheimer's disease
**Eu000**		Dementia in Alzheimer's disease with early onset	**F00.0**	Dementia in Alzheimer's disease with early onset
			**G30.0**	
**Eu001**	**X0030**	Dementia in Alzheimer's disease with late onset	**F00.1**	Dementia in Alzheimer disease with late onset
			**G30.1**	
	**XaIKC**	Alzheimer's disease with late onset		
**Eu002**	**Eu002**	Dementia in Alzheimer's disease, atypical or mixed type	**F00.2**	Dementia in Alzheimer disease, atypical or mixed type
			**G30.8**	Other Alzheimer's disease
**F110**		Dementia in Alzheimer's disease, unspecified	**F00.9**	Dementia in Alzheimer's disease, unspecified
			**G30.9**	
**Eu01**	**XE1Xs**	Vascular dementia	**F01**	Vascular dementia
			**F01.0**	Vascular dementia of acute onset
**Eu011**	**Xa0IH**	Multi-infarct dementia	**F01.1**	Multi-infarct dementia
**Eu012**	**X003T**	Subcortical vascular dementia	**F01.2**	Subcortical vascular dementia
**Eu01y**	**Eu01y**	Other vascular dementia	**F01.8**	Other vascular dementia
**Eu01z**	**Eu01z**	Vascular dementia, unspecified	**F01.9**	Vascular dementia, unspecified
			**F02**	Dementia in other diseases classified elsewhere
**Eu020/F111**	**X0034/F111**	Picks Disease (Read)/Frontotemporal dementia includes Picks Disease and progressive isolated aphasia(CTv3)	**F02.0**	Dementia in Pick's disease
			**F02.1**	Dementia in Creutzfeldt-Jakob disease
			**F02.2**	Dementia in Huntington's disease
**Eu023**	**Eu023**	Dementia in Parkinson's disease	**F02.3**	Dementia in Parkinson's disease
			**F02.8**	Dementia in other specified diseases classified elsewhere

For peer review only - http://bmjopen.bmj.com/site/about/guidelines.xhtml

Table S1: Continued

**Eu02y**	**X0034**	Dementia in other diseases specified elsewhere (Read)/Frontotemporal dementia includes Pick's Disease and progressive isolated aphasia	**G31**	Other degenerative diseases of nervous system, not elsewhere classified
			**G31.0**	Frontotemporal dementia
**Eu025 /F116**	**X003A**<br><br>**XaKyY**	Lewy body dementia<br><br>Lewy body dementia	**G31.8**	Other specified degenerative diseases of nervous system<br>Grey-matter degeneration [Alpers]<br>Lewy body(ies)(dementia)(disease)<br>Subacute necrotizing encephalopathy [Leigh]
**Eu02z**	**XE1Z6**	Unspecified dementia	**F03**	Unspecified dementia
			**F05.1**	Delirium superimposed on dementia
	**X002w**	Dementia		
	**X00R2**	Senile dementia		
**E00..**	**E00..**	Senile/presenile dementia		
**1461**	**1461**	History of dementia		
**Eu107**<br>**Eu10711**<br>**E012**<br>**E0120**	**Xa25J**	Alcoholic dementia	**F10.7**	Residual and late-onset psychotic disorder: Includes Alcoholic dementia NOS<br>Chronic alcoholic brain syndrome<br>Dementia and other milder forms of persisting impairment of cognitive functions

Anti-Dementia Medications

- Donezepil (Aricept®Aricept Evess®) Read Code: dy1
- Galantamine (Reminyl®, Reminyl® XL) Read Code: dy3…
- Rivastigimine (Exelon) Read Code: dy2…
- Memantine hydrochloride Read Code: dB1..

review only

For peer review only - http://bmjopen.bmj.com/site/about/guidelines.xhtml

Table S2: Comparison of socio-demographic characteristics between sub-population (N=4668) and rest of EPIC-Norfolk cohort.

	**Cohort**		**GP Sub-cohort**		**P-Value**
	**N=25 777***		**N=4668**		
Socio-demographic					
**Mean (SD)**					
**Age at Baseline**	59.9	(9.6)	56.2	(8.0)	<0.001
**Age at dementia diagnosis**	83.7	(6.6)	84.4	(6.6)	0.7
**Sex, % women (n)**	54.6	(14 063)	57.4	(2681)	<0.001
**Education, % (n)**					
No qualifications	39.8	(10 240)	33.1	(1546)	
O/ A level Standard	48.6	(12 510)	51.6	(2407)	<0.001
Graduate Level	11.6	(2992)	15.2	(711)	
**Social Class, % (n)**					
Professional	6.4	(1616)	8.5	(388)	
Managerial	35.2	(8833)	35.5	(1627)	
Skilled Non-Manual	16.7	(4199)	15.2	(697)	<0.001
Skilled Manual	23.7	(5948)	23.2	(1062)	
Semi-Skilled	14.1	(3545)	13.0	(597)	
Non-Skilled	3.8	(945)	4.6	(211)	
Dementia, % (N)	10.1	(2609)	4.4	(209)	<0.001

* Using full cohort as the sub population was based on this denominator and not 25,639 from baseline

For peer review only

For peer review only - http://bmjopen.bmj.com/site/about/guidelines.xhtml

Table S3: Summary of responses from 8 of the collaborating Practices providing GP data.

Response from Questionnaire sent to GP Practice	Number
Used both Read Code and Medication names in searches	7
Included additional dementia codes not listed in the 'Clean up exercise' protocol	4
Information available in records as coded and free text data (free text not provided for this study)	6 (0 Practices used free text alone)
Practices experiencing difficulty with using dementia Read Codes	6=No 1=Yes<br>1-No response
Does the Practice carry out In-house cognitive testing (Yes)	7
Reason for patient cognitive assessment/referral to memory clinic	7=Patient concern<br>6= GP Judgement<br>6= Opportunistic testing
How is a confirmed dementia diagnosis made	8= Following secondary referral<br>4= including GP judgement
How is information of a dementia diagnosis received by the Practice	7= Free text (As a letter)<br>1= Free text plus ICD code
How is information of a dementia diagnosis entered onto the local GP database	7= Read Code<br>1= (Free text, ICD and Read code)
Were all cases identified from searches already included on QOF* register	3=Yes<br>5=No
Was there a discrepancy between QOF* register and Read Code search	5=Yes<br>3=No

* Quality and Outcomes Framework

Dementia from HES records, death certificate or the MHDS was defined as any of the ICD-10 codes as listed in Supplementary Table S1.

References

1 Russell P, Banerjee S, Watt J, *et al.* Improving the identification of people with dementia in primary care: evaluation of the impact of primary care dementia coding guidance on identified prevalence. *BMJ Open* 2013;**3**:e004023. doi:10.1136/bmjopen-2013-004023

2 London Clinical Networks. "Coding clean-up " Exercise Guidance to GPs to improve dementia coding , and raise diagnosis rates: A step by step approach. London, UK:

3 Mukadam N, Livingston G, Rantell K, *et al.* Diagnostic rates and treatment of dementia before and after launch of a national dementia policy: An observational study using English national databases. *BMJ Open* 2014;**4**:1–8. doi:10.1136/bmjopen-2013-004119

4 Shohaimi S, Welch A, Bingham S, *et al.* Residential area deprivation predicts fruit and vegetable consumption independently of individual educational level and occupational social class: a cross sectional population study in the Norfolk cohort of the European Prospective Investigation into Canc. *J Epidemiol Community Health* 2004;**58**:686–91. doi:10.1136/jech.2003.008490

For peer review only

The RECORD statement – checklist of items, extended from the STROBE statement, that should be reported in observational studies using routinely collected health data.

	Item No.	STROBE items	Location in manuscript where items are reported	RECORD items	Location in manuscript where items are reported
**Title and abstract**					
	1	(a) Indicate the study's design with a commonly used term in the title or the abstract (b) Provide in the abstract an informative and balanced summary of what was done and what was found		RECORD 1.1: The type of data used should be specified in the title or abstract. When possible, the name of the databases used should be included.<br><br>RECORD 1.2: If applicable, the geographic region and timeframe within which the study took place should be reported in the title or abstract.<br><br>RECORD 1.3: If linkage between databases was conducted for the study, this should be clearly stated in the title or abstract.	Title and abstract<br><br>Title and abstract<br><br>Title and abstract
**Introduction**					
Background rationale	2	Explain the scientific background and rationale for the investigation being reported			Pages 3-4
Objectives	3	State specific objectives, including any prespecified hypotheses			Page 4
**Methods**					
Study Design	4	Present key elements of study design early in the paper			Page 5-6 Supplementary Information Page 1
Setting	5	Describe the setting, locations, and relevant dates, including periods of recruitment, exposure, follow-up, and data collection			Pages 5-6 Supplementary Information Page 1

For peer review only

Participants	6	*(a) Cohort study* - Give the eligibility criteria, and the sources and methods of selection of participants. Describe methods of follow-up<br>*Case-control study* - Give the eligibility criteria, and the sources and methods of case ascertainment and control selection. Give the rationale for the choice of cases and controls<br>*Cross-sectional study* - Give the eligibility criteria, and the sources and methods of selection of participants<br><br>*(b) Cohort study* - For matched studies, give matching criteria and number of exposed and unexposed<br>*Case-control study* - For matched studies, give matching criteria and the number of controls per case		RECORD 6.1: The methods of study population selection (such as codes or algorithms used to identify subjects) should be listed in detail. If this is not possible, an explanation should be provided.<br><br>RECORD 6.2: Any validation studies of the codes or algorithms used to select the population should be referenced. If validation was conducted for this study and not published elsewhere, detailed methods and results should be provided.<br><br>RECORD 6.3: If the study involved linkage of databases, consider use of a flow diagram or other graphical display to demonstrate the data linkage process, including the number of individuals with linked data at each stage.	Page 5-6<br>`Figure 1<br><br>Page 6<br>Figure 1
Variables	7	Clearly define all outcomes, exposures, predictors, potential confounders, and effect modifiers. Give diagnostic criteria, if applicable.		RECORD 7.1: A complete list of codes and algorithms used to classify exposures, outcomes, confounders, and effect modifiers should be provided. If these cannot be reported, an explanation should be provided.	Supplementary Information Page 1 and Table S1/<br><br>Page 8
Data sources/ measurement	8	For each variable of interest, give sources of data and details of methods of assessment (measurement).<br>Describe comparability of assessment methods if there is more than one group			Supplementary Information Pages1-2<br><br>Pages 5-6

For peer review only

Bias	9	Describe any efforts to address potential sources of bias			Supplementary Information Page 2
Study size	10	Explain how the study size was arrived at			Page 5 Figure 1
Quantitative variables	11	Explain how quantitative variables were handled in the analyses. If applicable, describe which groupings were chosen, and why			Supplementary Information Page 2<br><br>Page 6
Statistical methods	12	(a) Describe all statistical methods, including those used to control for confounding<br>(b) Describe any methods used to examine subgroups and interactions<br>(c) Explain how missing data were addressed<br>(d) *Cohort study* - If applicable, explain how loss to follow-up was addressed<br>*Case-control study* - If applicable, explain how matching of cases and controls was addressed<br>*Cross-sectional study* - If applicable, describe analytical methods taking account of sampling strategy<br>(e) Describe any sensitivity analyses			Supplementary Information Page 2<br><br>Page 6<br><br>Supplementary Information Page 2<br><br>Page 6
Data access and cleaning methods		..		RECORD 12.1: Authors should describe the extent to which the investigators had access to the database population used to create the study population.	Supplementary Information Page 2<br><br>Page 6

For peer review only

				RECORD 12.2: Authors should provide information on the data cleaning methods used in the study.	
Linkage		..		RECORD 12.3: State whether the study included person-level, institutional-level, or other data linkage across two or more databases. The methods of linkage and methods of linkage quality evaluation should be provided.	Supplementary Information Page 2<br><br>Page 6
**Results**					
Participants	13	(a) Report the numbers of individuals at each stage of the study (*e.g.*, numbers potentially eligible, examined for eligibility, confirmed eligible, included in the study, completing follow-up, and analysed)<br>(b) Give reasons for non-participation at each stage.<br>(c) Consider use of a flow diagram		RECORD 13.1: Describe in detail the selection of the persons included in the study (*i.e.,* study population selection) including filtering based on data quality, data availability and linkage. The selection of included persons can be described in the text and/or by means of the study flow diagram.	Figure 1<br>Pages 7-8<br>Supplementary Table S2
Descriptive data	14	(a) Give characteristics of study participants (*e.g.*, demographic, clinical, social) and information on exposures and potential confounders<br>(b) Indicate the number of participants with missing data for each variable of interest<br>(c) *Cohort study* - summarise follow-up time (*e.g.*, average and total amount)			Tables 1 and 2 and Table S2
Outcome data	15	*Cohort study* - Report numbers of outcome events or summary measures over time<br>*Case-control study* - Report numbers in each exposure			Pages 7-12

For peer review only

		category, or summary measures of exposure<br>*Cross-sectional study* - Report numbers of outcome events or summary measures			
Main results	16	(a) Give unadjusted estimates and, if applicable, confounder-adjusted estimates and their precision (e.g., 95% confidence interval). Make clear which confounders were adjusted for and why they were included<br>(b) Report category boundaries when continuous variables were categorized<br>(c) If relevant, consider translating estimates of relative risk into absolute risk for a meaningful time period			Table 3<br>Figures 3 and 4
Other analyses	17	Report other analyses done—e.g., analyses of subgroups and interactions, and sensitivity analyses			Pages 11-12
**Discussion**					
Key results	18	Summarise key results with reference to study objectives			Page 13
Limitations	19	Discuss limitations of the study, taking into account sources of potential bias or imprecision. Discuss both direction and magnitude of any potential bias		RECORD 19.1: Discuss the implications of using data that were not created or collected to answer the specific research question(s). Include discussion of misclassification bias, unmeasured confounding, missing data, and changing eligibility over time, as they pertain to the study being reported.	Page 13
Interpretation	20	Give a cautious overall interpretation of results considering objectives,			Pages 13-17

For peer review only

		limitations, multiplicity of analyses, results from similar studies, and other relevant evidence			
Generalisability	21	Discuss the generalisability (external validity) of the study results			Page 16
**Other Information**					
Funding	22	Give the source of funding and the role of the funders for the present study and, if applicable, for the original study on which the present article is based			Page 17
Accessibility of protocol, raw data, and programming code		..		RECORD 22.1: Authors should provide information on how to access any supplemental information such as the study protocol, raw data, or programming code.	Page 17

*Reference: Benchimol EI, Smeeth L, Guttmann A, Harron K, Moher D, Petersen I, Sørensen HT, von Elm E, Langan SM, the RECORD Working Committee. The REporting of studies Conducted using Observational Routinely-collected health Data (RECORD) Statement. *PLoS Medicine* 2015; in press.

*Checklist is protected under Creative Commons Attribution (CC BY) license.

For peer review only
